# New 1,2,3-Triazole/1,2,4-triazole Hybrids as Aromatase Inhibitors: Design, Synthesis, and Apoptotic Antiproliferative Activity

**DOI:** 10.3390/molecules28207092

**Published:** 2023-10-14

**Authors:** Mohamed T-E Maghraby, Tahani Mazyad Almutairi, Stefan Bräse, Ola I. A. Salem, Bahaa G. M. Youssif, Mahmoud M. Sheha

**Affiliations:** 1 Department of Pharmaceutical Organic Chemistry, Faculty of Pharmacy, Assiut University, Assiut 71526, Egypt; tag_84@yahoo.com (M.T.-E.M.); olasalem69@gmail.com (O.I.A.S.); 2 Department of Pharmaceutical Chemistry, Faculty of Pharmacy, New Valley University, New Valley 72511, Egypt; 3 Department of Chemistry, College of Science, King Saud University, Riyadh 11451, Saudi Arabia; talmutari1@ksu.edu.sa; 4 Institute of Biological and Chemical Systems, IBCS-FMS, Karlsruhe Institute of Technology, 76131 Karlsruhe, Germany; 5 Department of Medicinal Chemistry, Faculty of Pharmacy, Assiut University, Assiut 71526, Egypt; sheha@sphinx.edu.eg; 6 Department of Pharmaceutical Chemistry, Faculty of Pharmacy, Sphinx University, New-Assiut 71684, Egypt

**Keywords:** 1,2,3-triazole, 1,2,4-triazole, anticancer, aromatase, viability

## Abstract

A novel series of 1,2,3-triazole/1,2,4-triazole hybrids 5a, 5b, and 6a–i was designed and synthesized as antiproliferative agents targeting aromatase enzymes. The antiproliferative activity of the new hybrids against four cancer cells was studied using Erlotinib as a control. Compounds 6a and 6b demonstrated the highest antiproliferative activity among these hybrids, with GI50 values of 40 nM and 35 nM, respectively. Compound 6b was the most potent derivative, with a GI50 of 35 nM, comparable to Erlotinib’s GI50 of 33 nM. Compound 6b inhibited all cancer cell lines with comparable efficacy to Erlotinib. Compounds 5a, 5b, and 6a–i were tested for inhibitory action against aromatase as a potential target for their antiproliferative activity. Results revealed that compounds 6a and 6b were the most potent aromatase inhibitors, with IC50 values of 0.12 ± 0.01 µM and 0.09 ± 0.01 µM, respectively, being more potent than the reference Ketoconazole (IC50 = 2.6 ± 0.20 µM) but less potent than Letrozole (IC50 = 0.002 ± 0.0002). These findings indicated that compounds 6a and 6b had significant aromatase inhibitory action and are potential antiproliferative candidates. The findings were further linked to molecular docking investigations, which gave models of strong interactions with the aromatase domain for inhibitors with high binding scores.

## 1. Introduction

**Figure 1:**
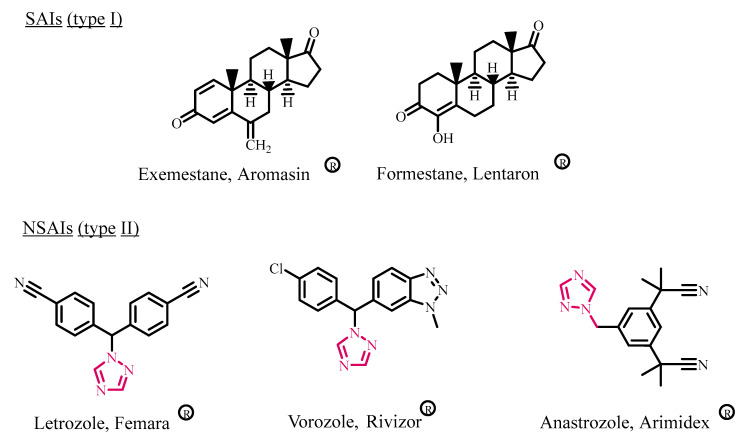
Structures of SAIs (type **I**) and NSAIs (type **II**) aromatase inhibitors.

High estrogen levels have been linked to cancer cell proliferation, recurrence, and metastasis in estrogen-dependent breast malignancies. One of the most effective breast cancer management strategies is reducing estrogen levels by preventing its manufacturing [1,2,3]. Aromatase (CYP19) is a rate-limiting enzyme in the cytochrome P450 family that catalyzes the production of estrogens from androgens. As a result, aromatase inhibitors (AIs) have become one of the most utilized medication classes for treating estrogen-dependent cancer [4,5,6,7]. AIs are classified into two types on the basis of their mechanisms of action: (i) steroidal AIs (such as formestane and exemestane) that inhibit the aromatase enzyme irreversibly, and (ii) nonsteroidal AIs (such as Letrozole, anastrozole, and vorozole) whose inhibitory effects are reversible (Figure 1) [8,9,10].

Although currently available aromatase inhibitors (both steroidal and nonsteroidal types) have demonstrated successful clinical outcomes, long-term use can result in acquired drug resistance as well as significant side effects such as musculoskeletal pain, bone loss (osteoporosis), broken bones, and cardiovascular disease [11,12]. As a result, the development of novel aromatase inhibitors is still required to provide a more suitable alternative medicine of choice.

Azoles are aromatic heterocycles with five members that contain a nitrogen atom and at least one other hetero (non-carbon) element (e.g., nitrogen, sulfur, or oxygen). Because of its broad biological importance, azole is an important structural class of heterocycles [13,14,15]. Extensive molecular docking experiments have revealed that the azole ring connects with iron in the core of the heme moiety present in the active site of aromatase [16,17]. As a result, this azole family has proven to be an effective AI. Anastrozole, vorozole, and Letrozole (Figure 1), for example, have been shown to decrease aromatase activity by 93%, 98%, and 99.9%, respectively [18,19].

**Figure 2:**
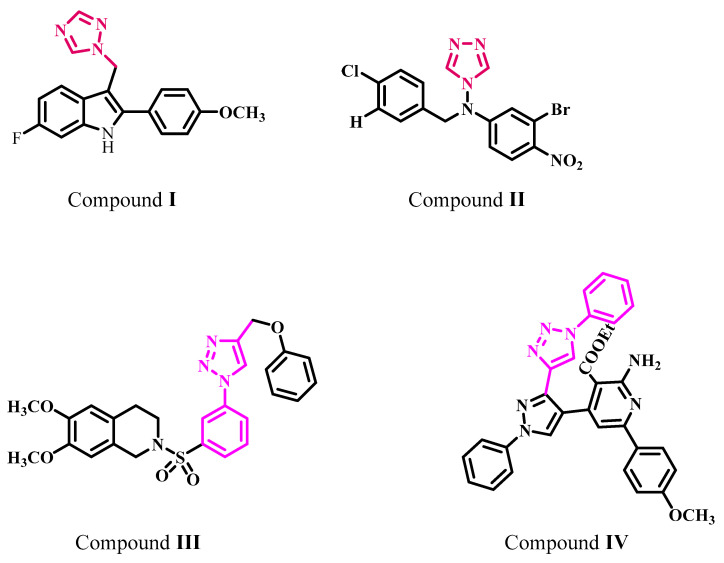
Structures of compounds **I–IV**.

Kang et al. described the synthesis, cytotoxicity, and aromatase inhibitory action of a 2-phenyl indole scaffold replaced with an azole ring at the 3-position [20]. Compound **I** (Figure 2) with a triazole ring had the most promising anti-aromatase action, with an IC_50_ value of 0.014 µM compared with the reference letrozole (IC_50_ = 0.049 µM). The potency was reduced when the triazole ring of compound I was replaced with an imidazole ring. The biological results showed that indole derivatives with a 1,2,4 triazole ring inhibit aromatase more effectively than imidazole-containing derivatives, which could be related to the particular orientation and polarity of triazole in the aromatase binding pocket.

Songa et al. synthesized 4-N-nitrophenyl substituted amino-4*H*-1,2,4-triazole analogs and tested their aromatase inhibitory activity [21]. Compound **II**, Figure 2, was the most effective AI, with an IC_50_ value of 9.02 nM. This investigation showed that substituting EWG at the *para*-position on the benzyl ring had a greater influence on increasing activity than substituting EWG at the *meta*-position.

In another study, Pingaew et al. reported a variety of 1,4-disubstituted-1,2,3-triazoles having a sulfonamide moiety and investigated their aromatase inhibitory efficacies [22]. Analogue **III** (Figure 2) has the most effective aromatase inhibitory activity, with an IC50 value of 0.2 M. Structure activity relationship (Structure activity relationship, SAP) shows that the lipophilic character of the methoxy group improves the binding affinities of the newly produced compounds with aromatase.

Moreover, El-Naggar et al. developed a novel series of 1,2,3 triazole compounds found to be strong aromatase inhibitors [23]. Compared with the reference medication letrozole (IC_50_ = 2.8 nM), compound **IV** (Figure 2) demonstrated significant aromatase inhibition efficacy with an IC_50_ value of 24 nM. Physical parameters such as solubility and the molecular mass of various groups were shown to play important roles in the inhibitory potency of synthesized derivatives.

**Figure 3:**
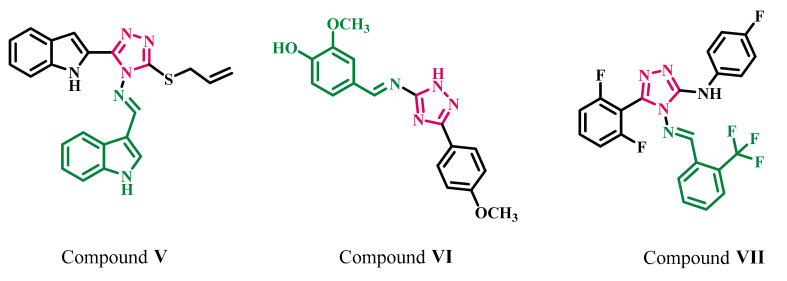
Structures of Schiff bases derived from 1,2,4-triazoles **V**–**VII**.

On the other hand, Schiff bases, distinguished by an azomethine group (-C=N-), have gained prominence because of various pharmacological actions, including anticancer activity [24,25,26]. Many investigations on 1,2,4-triazole-Schiff bases have been identified in terms of anticancer activity, demonstrating that this core has substantial biological activity as anticancer medicines. Nafie et al. [27], for example, reported on novel alkylated indolyl-triazole Schiff bases for breast cancer treatment. Compound **V** (Figure 3) showed promising antiproliferative activity against the breast cancer (MCF-7) cell line with an IC_50_ value of 1.18 µM when compared with Sorafenib (IC_50_ = 2.13 µM). El-Sherief et al. [25] also reported novel 1,2,4-triazole/Schiff base hybrids with EGFR and BRAF inhibitory properties. For example, derivative **VI** (Figure 3) displayed substantial anticancer activity against a panel of four cancer cell lines, with IC_50_ ranging from 1.3 to 2.6 µM compared with Erlotinib (IC_50_ range from 0.02 to 0.04 µM).

Additionally, Kumar et al. [28] have revealed the antiproliferative efficacy of some novel fluorinated Schiff bases generated from 1,2,4-triazoles. Compound **VII** (Figure 3) displayed good antiproliferative activity, with 73%, 77%, and 69% growth inhibitory activity at 10 µM against Colo-205, MDA-MB 231, and IMR-32 cell lines, respectively.

**Figure 4:**
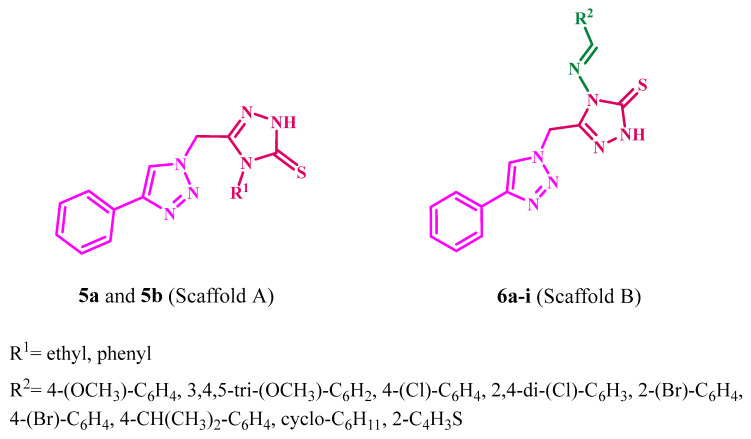
Structures of new hybrids **5a**, **5b**, and **6a**–**i**.

Motivated by the observations shown above, we present the synthesis of a novel series of 1,2,3-triazole/1,2,4-triazole hybrids **5a**, **5b**, and **6a**–**i** (Figure 4) as aromatase inhibitors. The new hybrids’ structures were validated using ^1^H NMR, ^13^C NMR, and elemental microanalysis. Using an MTT assay, all the synthesized compounds were tested for antiproliferative efficacy against four human cancer cell lines. The aromatase inhibitory assay assessed the antiproliferative mechanism of novel hybrids. Molecular docking explored the most active derivatives’ binding processes and interactions within the target enzyme’s active site.

## 2. Results and Discussion

### 2.1. Chemistry

**Scheme 1:**
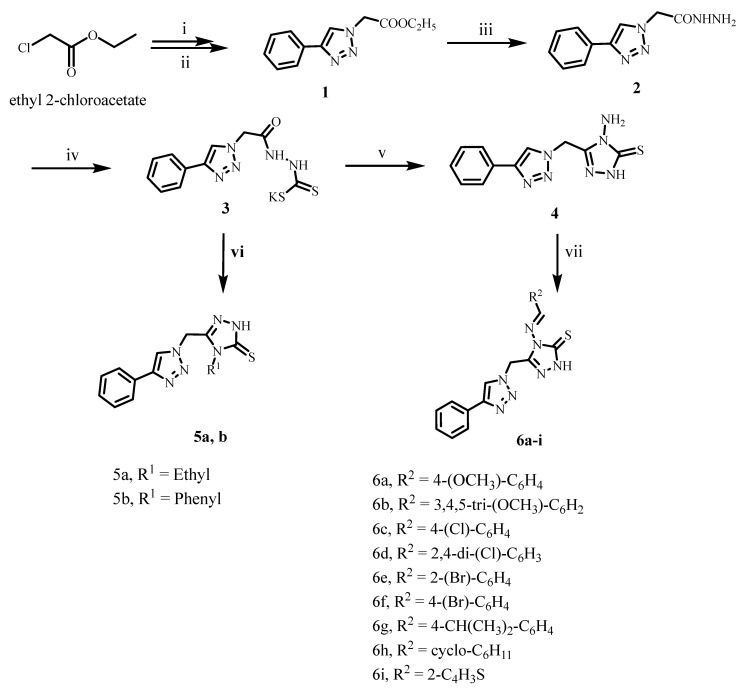
Synthesis of target compounds **5a**, **5b**, and **6a**–**i**.

The synthesis of target compounds **5a**, **5b**, and **6a**–**i** is depicted in Scheme 1. Compound **1** was synthesized using the click reaction. First, ethyl 2-azidoacetate was synthesized by reacting ethyl 2-chloroacetate with two equivalents of sodium azide in acetone and heating at reflux for 24 h. The liquid product obtained following ethyl acetate extraction was subjected to the second reaction step with a combination of phenylacetylene, CuSO_4_·5H_2_O, and sodium ascorbate in THF: H_2_O (1:1 *v/v*). After 24 h of stirring at room temperature, ethyl 2-(4-phenyl-1*H*-1,2,3-triazol-1-yl)acetate 7 was obtained in 96% yield [29]. Acetohydrazide derivative **2** was produced in 90% yield by reacting compound **1** with an excess of 99% hydrazine hydrate in absolute ethanol [29].

Compounds **3** and **4** were prepared using a process previously reported [30]. Potassium carbodithioate salt **3** was synthesized by reacting acetohydrazide **2** with carbon disulfide in ethanol in the presence of potassium hydroxide. The reaction was finished after 16 h of stirring at room temperature. The suspension of potassium carbodithioate salt **3** (without further purification) and hydrazine hydrate 99% (two equivalents) was then heated at reflux with stirring for 2 h. The resultant 3*H*-1,2,4-triazole-3-thione derivative **4** was recrystallized from ethanol in high yield (91%) after being acidified with hydrochloric acid to pH 7–8. The proposed mechanism of heterocyclization of potassium carbodithioate salt **3** with hydrazine hydrate was accomplished by Ji et al. [31]. The salt **3** underwent ring closure with an excess of hydrazine hydrate to give the key intermediate 4-amino-1,2,4-triazole-3-thione **4** (See Supplementary Material).

**Reagents and reactions conditions:** (**i**) NaN_3_, acetone, reflux, 24 h, 89%; (**ii**) phenyl acetylene, Na ascorbate, CuSO_4_, THF:H_2_O (1:1), RT, 24 h, 96%; (**iii**) NH_2_NH_2_·H_2_O, Ethanol, reflux 6 h, 90%; (**iv**) CS_2_, KOH, Ethanol, RT 16 h; (**v**) NH_2_NH_2_.H_2_O, reflux 2 h, 91%; (**vi**) R^1^-N=C=S, *n*-butanol, reflux 20–24 h, 84–86%; (**vii**) R-CHO, glacial acetic acid, Ethanol, reflux, 12–15 h, 78–85%.

The novel synthesized compounds **5a** and **5b** were obtained in 84% and 86% yields, respectively, through the reaction of potassium carbodithioate **3** and the appropriate substituted isothiocyanate (1.1 equivalents) in *n*-butanol. The ^1^H NMR spectrum of compound **5a** in DMSO-*d6* (400 MHz, δ *ppm*) revealed a triplet signal (3H) at 0.96 *ppm* and a quartet signal (2H) at 3.95 *ppm*, both of which are characteristic of the ethyl group with coupling constant (J) equal to 7.10 Hz. A singlet (2H) at 5.84 *ppm* was also ascribed to the methylene linker. Furthermore, the spectrum revealed a multiplet signal (6H) at 7.34–7.87 *ppm*, corresponding to five aromatic protons overlapping with the proton of the triazole ring. Finally, the spectra revealed a singlet signal (1H) at 8.64 *ppm* that could be exchanged with D_2_O corresponding to NH. The ^13^C NMR spectrum (100 MHz) of compound **5a** revealed two signals at 13.25 and 22.38 *ppm*, corresponding to ethyl group carbons. Furthermore, seven signals between 122.00 and 146.89 ppm were compatible with aromatic, 1,2,3-triazole, and 1,2,4-triazole-3-thione ring carbons. Furthermore, a signal at 167.31 ppm was matched with C=S, a characteristic signal for the 1,2,4-triazole-3-thione ring. Finally, the EI-MS spectrum of compound **5a** revealed the molecular ion peak *M^.+^* at *m*/*z* (286.57; 63%), corresponding to molecular weight (M.F., C_13_H_14_N_6_S).

**Scheme 2:**
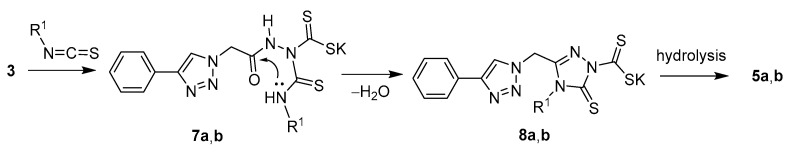
Proposed mechanism for the formation of compounds **5a** and **5b**.

Scheme 2 depicts a proposed mechanism for the formation of compounds **5a** and **5b**. The reaction begins with the nucleophilic addition of **3** on the appropriate isothiocyanate to generate **7a**,**b**. In the subsequent step, these intermediates undergo cyclocondensation, constructing triazoles **8a**,**b**, from which hydrolysis finally affords **5a**,**b** as isolated products.

On the other hand, the novel synthesized compounds **6a**–**i** were produced in 77–85% yields through the reaction of an equivalent amount of 4-amino-5-((4-phenyl-1*H*-1,2,3-triazol-1-yl)methyl)-2,4-dihydro-3*H*-1,2,4-triazole-3-thione **4** with the appropriate aryl aldehyde, cyclohexane carbaldehyde, or thiophene-2-carbaldehyde in the presence of a catalytic amount of glacial acetic acid in absolute ethanol. The structures of new hybrids **6a**–**i** were confirmed by spectroscopy (^1^H NMR, ^13^C NMR, EI-Mass) and elemental analyses. For example, the ^1^H NMR spectrum of compound **6b** in DMSO-d_6_ (400 MHz, δ *ppm*) showed a singlet signal (3H) at δ: 3.64 *ppm* assigned to the OCH_3_ group with the appearance of another singlet signal (6H) at δ: 3.77 *ppm*, which attributed to another two equivalent OCH_3_ groups. Additionally, the spectrum showed a singlet signal (2H) at δ: 5.45 *ppm* assigned for methylene linker, and the multiplet signal (8H) at δ: 6.87–7.27 *ppm* corresponded to aromatic rings and CH=N protons. Also, the spectrum displayed a characteristic singlet signal (1H) at δ: 8.17 *ppm* corresponding to triazole H. Finally, the appearance of a singlet (1H) at δ: 10.39 *ppm,* which was exchangeable with D_2_O, was assigned to NH. All demonstrate the formation of novel 1,2,3-triazole/1,2,4-triazole **6a**–**i** derivatives.

### 2.2. Biology

#### 2.2.1. Cell Viability Assay

To investigate the effect of compounds **5a**, **5b**, and **6a**–**i** on normal cell lines, a cell viability assay was performed on the MCF-10A (human mammary gland epithelial) cell line [32,33]. This study used 50 µM of the investigated compound for four days before assessing cell viability. As shown in Table 1, compounds **5a**, **5b**, and **6a**–**i** have no toxic effect and have greater than 87% cell viability.

#### 2.2.2. Antiproliferative Assay

**Figure 5:**
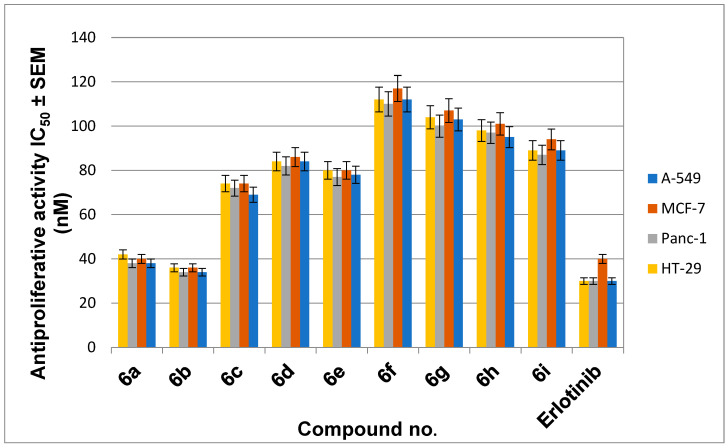
Antiproliferative activity of compounds **6a**–**i**.

The newly synthesized compounds **5a**, **5b**, and **6a**–**i** were tested for antiproliferative activity against four different types of cancer cells: A-549 (epithelial cancer cell line), MCF-7 (breast cancer cell line), Panc-1 (pancreas cancer cell line), and HT-29 (colon cancer cell line) using Erlotinib as the reference drug [34,35]. The results of calculating the IC_50_ of each compound are shown in Table 1, Figure 5.

Compounds **5a** (R^1^ = ethyl, Scaffold A) and **5b** (R^1^ = phenyl, Scaffold A) were the least potent derivatives with GI_50_ values greater than 120 nM among the newly synthesized compounds, demonstrating that the 1,2,4-triazole-2-thione moiety is not tolerated for the antiproliferative actions of this class of organic compounds (Table 1).

In general, compounds **6a**–**i** of Scaffold B demonstrated promising antiproliferative activity against the four cancer cell lines tested, with GI_50_ ranging from 35 nM to 113 nM versus the reference Erlotinib, which had a GI_50_ of 33 nM. Compounds **6a** and **6b** had the highest antiproliferative activity, with GI_50_ values of 35 nM and 40 nM, respectively. Compound **6b** (Ar = 3,4,5-tri-OMe-Ph, Scaffold B) was the most potent derivative, with a GI_50_ of 35 nM, and it was equipotent with the reference Erlotinib, which had a GI_50_ of 33 nM. Compound **6b** suppressed all cancer cell lines with comparable efficacy to Erlotinib. Compound **6a** (Ar = 4-OMe-Ph, Scaffold B) ranked second in activity, with GI_50_ values of 40 nM, 1.2-fold less potent than the reference Erlotinib against the four cancer cell lines tested. These findings demonstrated the importance of the number of methoxy groups in Scaffold B compounds’ antiproliferative activity, with the tri-methoxy derivative **6b** outperforming the methoxy derivative **6a**.

The 4-chloro-substituted derivative **6c** (Ar = 4-Cl-Ph, Scaffold B) was 1.8-fold less potent than compound **6a**. In contrast, compounds **6d** (Ar = 2, 4-di-Cl-Ph, Scaffold B), **6e** (Ar = 2-Br-Ph, Scaffold B), and **6f** (Ar = 4-Br-Ph, Scaffold B) showed moderate or weak antiproliferative action with GI_50_ values of 84 nM, 79 nM, and 113 nM, respectively, indicating the importance of the nature and number of halogen atom substitutions on the benzylidene ring of compounds **6a**–**i**, with activity increasing in the order: 2-Br > 4-OCH_3_ > 2,4-di-Cl > 4-Br.

Compounds **6h** (Ar = cyclohexyl, Scaffold B) and **6i** (Ar = thiophene, Scaffold B) demonstrated weak antiproliferative activity with GI_50_ values of 98 nM and 90 nM being 2.8-fold and 2.6-fold less potent than **6b**, indicating the significance of tri-methoxy phenyl moiety for the antiproliferative action and that Phenyl > thiophene > cyclohexyl moiety in activity (Table 1).

#### 2.2.3. Aromatase Inhibitory Assay

**Figure 6:**
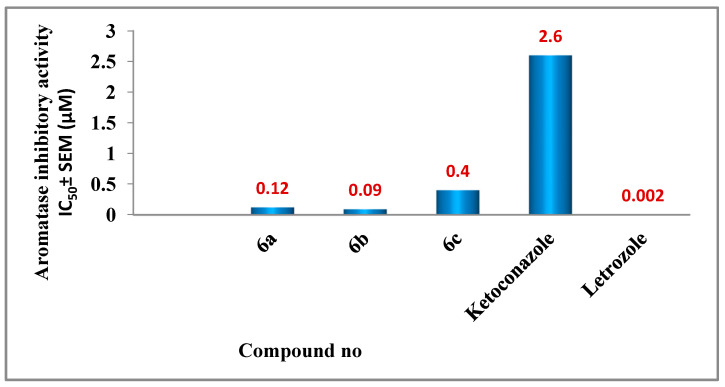
Aromatase inhibitory activity of compounds **6a**–**c**.

Compounds **5a, 5b**, and **6a**–**i** were tested for inhibitory action against aromatase as a potential target for their antiproliferative activity, using Ketoconazole and Letrozole as reference drugs [36]. Results are presented as IC_50_ values in Table 2 and Figure 6. The results of this assay are consistent with those of the antiproliferative assay, in which the most potent antiproliferative derivatives, compounds **6a** (Ar = 4-OCH_3_-Ph, Scaffold B) and **6b** (Ar = 3,4,5-tri-OCH_3_-Ph, Scaffold B), were the most potent aromatase inhibitors, with IC_50_ values of 0.12 ± 0.01 µM and 0.09 ± 0.01 µM, respectively, being more potent than the reference Ketoconazole (IC_50_ = 2.6 ± 0.20 µM) but less potent than Letrozole (IC_50_ = 0.002 ± 0.0002 µM).

Compound **6c** (Ar =4-Cl-Ph, Scaffold B) showed moderate anti-aromatase activity with an IC_50_ value of 0.40 ± 0.02 µM, whereas compounds **5a**, **5b**, and **6d**–**i** showed weak aromatase inhibitory activity with an IC_50_ value greater than 12.5 µM. These findings demonstrated that compounds **6a** and **6b** have potent aromatase inhibitory activity and are potential antiproliferative candidates.

#### 2.2.4. Apoptosis Markers Assays

One method of treating cancer is to regulate or stop the uncontrolled proliferation of cancer cells. Using the natural dying process of the cell is a highly successful strategy. Targeting apoptosis is effective for many types of cancer because apoptosis evasion is a feature of cancer and is not specific to the etiology or type of cancer. Many anticancer medications target different phases of both the intrinsic and extrinsic pathways [37,38,39]. The ability of compounds **6a**–**c**, the most effective derivatives in all in vitro investigations, to initiate the apoptosis cascade and disclose their proapoptotic potential was investigated.

##### Caspase-3 Activation Assay

Caspases are required for apoptosis induction and maintenance. Caspase-3 is a vital caspase that cleaves numerous cell proteins, resulting in apoptosis [40,41]. Compounds **6a**–**c** were tested as caspase-3 activators against the human epithelial (A-594) cancer cell line [42], with Table 3 displaying the results.

Compounds **6a** and **6b** showed promising caspase-3 protein overexpression levels of 494 ± 4 and 525 ± 5 pg/mL, respectively. They elevated the protein caspase-3 in the A-594 cancer cell line by nearly eightfold when compared with untreated control cells. Compounds **6a** and **6b** were more active than control staurosporine, which had a caspase-3 overexpression level of 465 ± 4 pg/mL. Compound **6c** was the least derivative, with a caspase-3 overexpression of 325 ± 3 pg/mL, and even less active than the reference staurosporine. These findings suggested that compounds **6a** and **6b** have apoptotic potential, which could explain their antiproliferative action.

##### Caspase-8, Bax, Bcl-2 Levels Assays

The effects of compounds **6a** and **6b** on the levels of caspase-8, Bax, and the anti-apoptotic protein Bacl-2 against the A-594 cancel cell line were investigated further using staurosporine as a control. The outcomes are listed in Table 3.

Results from Table 3 revealed that compound **6b** (2.30 ng/mL) was detected to have the highest level of caspase-8 overexpression, which was followed by compound **6a** (2.05 ng/mL) and reference staurosporine (1.85 ng/mL). The tested **6a** and **6b** compounds increased caspase-8 levels by 23 and 25 times, respectively, in comparison with the untreated control cell.

Moreover, compounds **6a** and **6b** increased Bax induction 33- and 35-fold (295 pg/mL and 320 pg/mL, respectively) compared with untreated A-594 cancer cells, outpacing staurosporine (288 pg/mL, a 32-fold induction). Finally, when compared to staurosporine, derivatives **6a** and **6b** induced an equal downregulation of anti-apoptotic Bcl-2 protein levels in the A-594 cell line. These findings imply that compounds **6a** and **6b** are apoptosis inducers since they activate caspase-3, 8, and Bax and downregulate the anti-apoptotic Bcl-2.

### 2.3. Docking and Molecular Modeling Studies

The docking process involves the prediction of the favorable binding configurations between one or more small flexible ligands and a rigid/flexible macromolecular target, which is usually a protein. However, the search for binding modes is usually constrained to a smaller receptor region—the active site. The synthesized compounds were modeled by human placental aromatase cytochrome P450 co-crystallized with androstenedione (PDB code: 3EQM) [43].

**Figure 7:**
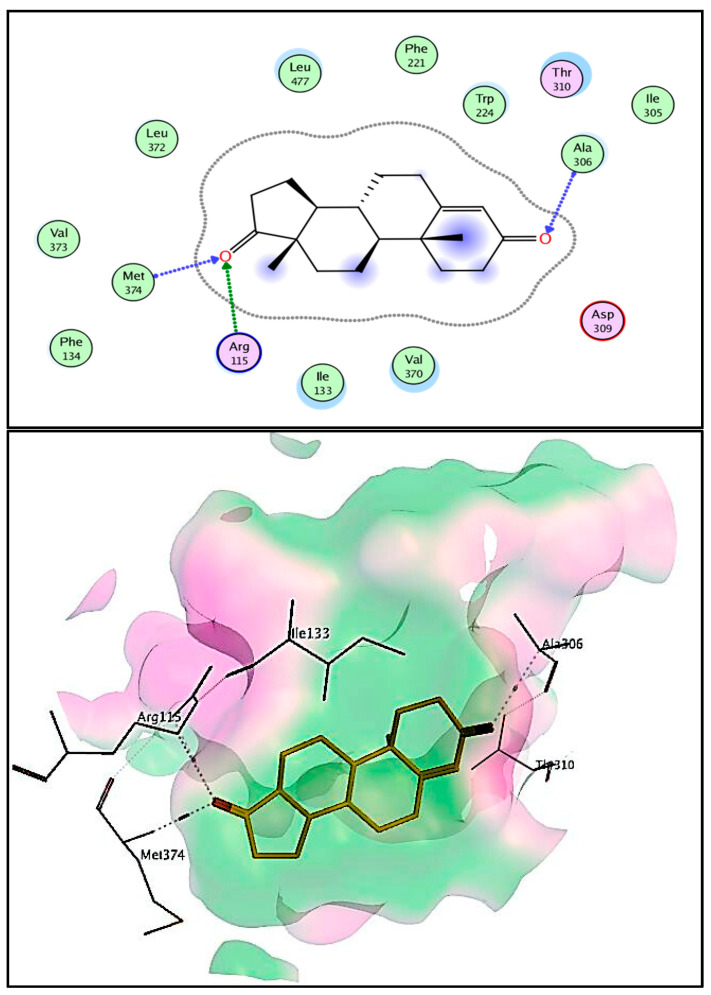
Two- and three-dimensional representation of the interaction of co-crystallized androstenedione with aromatase CP450 domain.

The crystal structure of aromatase cytochrome P450 in a complex with androstenedione drug was obtained from the protein data bank and used for docking studies. Binding modes and interactions of the co-crystallized androstenedione drug with aromatase cytochrome P450 were used to evaluate the proposed compounds’ binding because of binding similarities with the proposed compounds [44]. Androstenedione is a selective aromatase inhibitor with a binding score (−8.32 kcal/mole) that interacted mainly by hydrogen bond interaction of the carbonyl group with Ala306 (3.45 Å), Arg115 (3.44 Å), and Met374 (2.84 Å) as depicted in Figure 7.

Molecular docking simulations of the proposed compounds **6a**–**c** were performed to predict and evaluate the binding affinity of the proposed active compounds with aromatase CP450 enzyme. Furthermore, molecular docking studies help understand the binding mode and interactions between the ligands and amino acid residues in aromatase CP450.

The proposed compounds were constructed in a 2D model using ChemDraw software (Chemdraw 2014.0102, 2014) and then copied into the MOE interface, where the energies of the proposed compounds were minimized to obtain the most stable conformers, which were saved into the database; the latter was used in docking [34].

The X-ray crystallographic structure of aromatase CP450 enzyme co-crystallized with androstenedione (PDB code: 3EQM) was obtained from the protein databank, validated for docking, and prepared for docking. The docking simulations were performed by MOE dock application using Triangle Matcher as placement scheme, rigid receptor as refinement scheme, London ΔG as a scoring function, and GBVI/WSAdG as refinement score. The active site was selected as the pocket where the ligand is present, and docking was performed in the presence of the androstenedione ligand. The binding affinity of the docked molecules was evaluated by Score (S) kcal/mole, hydrogen bonds, and the hydrophobic interactions in the enzyme pocket. The pose of each compound, which reveals the highest binding affinity, is presented in Table 3 with their favorable interactions in the enzyme pocket.

The docked compounds interact with amino acid residues (Ala306, Arg115, Met374, Met311, Ser314, Val373, Arg375, Thr310, Cys437, Ala438, Gly436, Arg435, and Ile133) of androstenedione of aromatase by one or more (up to four) bonds type HB, π-π (hydrophobic), π-H, and H-π in addition to the exposure of fragments to one or more Leu477, Ile133, Arg145, Cys437, Trp224, Phe430, Arg435, Val370, Ala306, Leu152, Met446, and Met311 residues. The observed hydrogen bonding was formed by Ala306, Arg115, Met374, Met311, Ser314, Val373, Arg375, Thr310, Cys437, Ala438, Gly436, Arg435, and Ile133 while π-π staking was formed by Cys437, Val370, Val369, Ala438, Ala306, and Ile133. The most common amino acid residues in ligand binding forces type π-H and H-π are Val370, Cys437, and Ala306.

**Figure 8:**
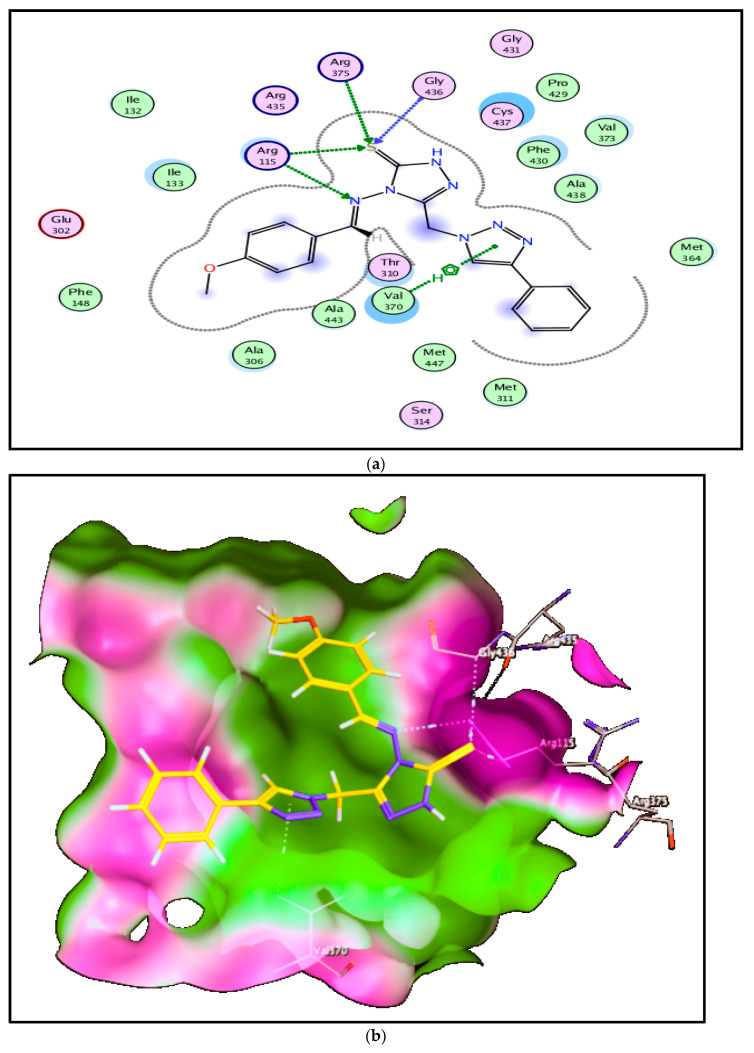
Mode of binding of compound 6a inside aromatase CP450 domain: (**a**) 2D structure and (**b**) 3D structure.

Compound **6a** with a high score (−9.64 kcal/mol) showed hydrogen bonding interactions by acting as hydrogen bond acceptor through heteroatoms S and N of this hybrid with hydroxyl moiety of amino acids Gly436, Arg375, and Arg115 (3.93, 4.49, and 3.79 Å, respectively), in addition to strong hydrophobic interaction between the triazole ring and Val370 amino acid (3.74 Å) (Figure 8).

**Figure 9:**
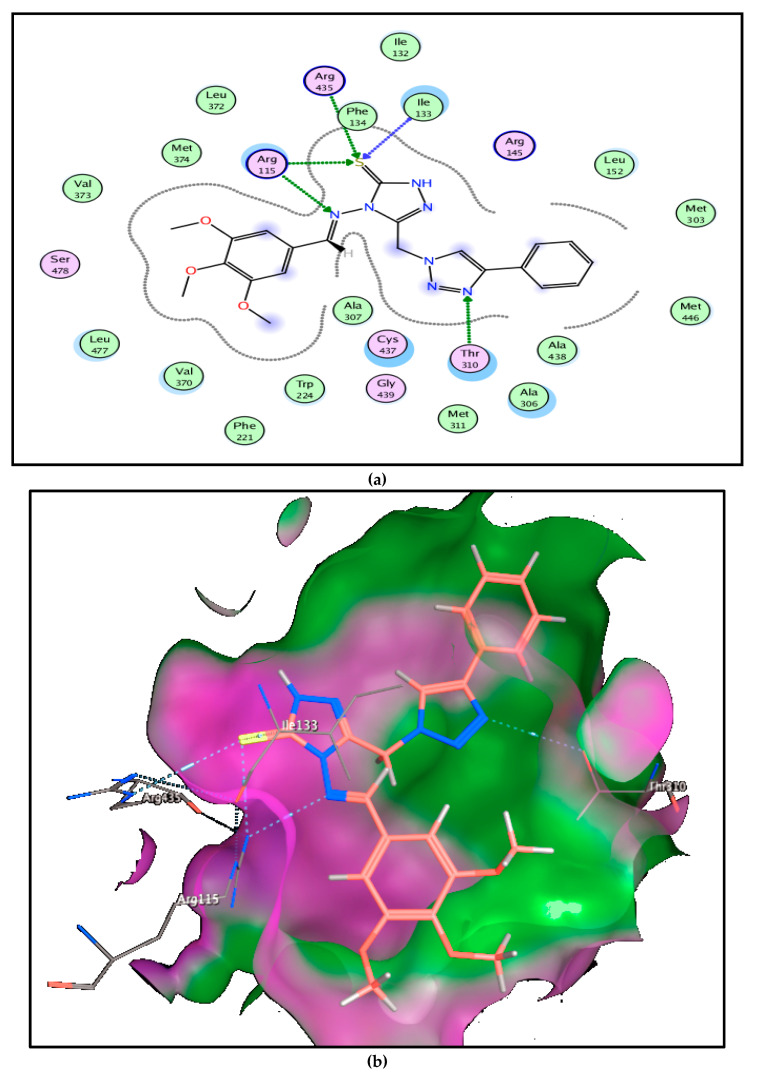
Mode of binding of compound **6b** inside aromatase CP450 domain: (**a**) 2D structure and (**b**) 3D structure.

The most potent aromatase inhibitor compound, **6b**, Figure 9, showed a higher binding score of −9.94 kcal/mol, comparable to the androstenedione binding score (−8.32 kcal/mol). Interaction between **6b** and amino acid residues within the pocket ranges from lipophilic interactions (with Val370, Leu477, Cys437, and Ala306 amino acids) to hydrogen bonding (with Arg115 amino acid), which is the main amino acid binding of androstenedione with aromatase CP450 domain, and this explains higher aromatase inhibitory activity (Table 4).

### 2.4. Structure–Activity Relationship (SAR) Analysis of **6a**–**i**


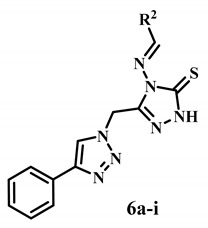


1—The nature, position, and number of atoms of benzylidene ring moiety of compounds **6a**–**i** all have a significant influence on compound activity, with antiproliferative activity increasing in the following order: 2-Br > 4-OCH_3_ > 2,4-di-Cl > 4-Br;

2—The phenyl group of benzylidene moiety substantially impacts antiproliferative activity and Phenyl > Thienyl > Cyclohexyl moiety in activity.

3—The number of methoxy groups on the phenyl ring of the benzylidene moiety appears to be crucial for aromatase inhibitory activity and possible antiproliferative candidates in compounds **6a**–**i**, with the tri-methoxy derivative (**6b**) outperforming methoxy derivative (**6a**).

## 3. Experimental

### 3.1. Chemistry

**General Details**: See Appendix SA.

Compounds **1** and **2** [29], **3** and **4** [30] were prepared according to reported procedure.

#### 3.1.1. General Method for the Synthesis of Compounds **5a** and **5b**

A mixture of potassium 2-(2-(4-phenyl-1*H*-1,2,3-triazol-1-yl)acetyl)hydrazine-1-carbodithioate **3** (10.0 mmol, 3.31 g) and the appropriate substituted isothiocyanate (11.0 mmol) in n-butanol (20 mL) was heated under reflux for 20–24 h. The n-butanol was evaporated at the end of the reaction, and the residue was washed with ice-cold methanol, filtered out, washed in water, and dried. Recrystallization from ethanol enabled solid purification. Compounds **5a** and **5b** were isolated with 84–86% yields.

##### 4-Ethyl-5-((4-phenyl-1H-1,2,3-triazol-1-yl)methyl)-2,4-dihydro-3H-1,2,4-triazole-3-thione (**5a**)

White crystals; yield 84%, mp 288–290 °C; ^1^H NMR (400 MHz, δ ppm DMSO-*d*_6_): 0.96 (t, 3H, *J* = 7.10 Hz, CH_2_CH_3_), 3.95 (q, 2H, *J* = 7.10 Hz, CH_2_CH_3_), 5.84 (s, 2H, CH_2_), 7.34–7.87 (m, 6H, Ar-H and triazole H), 8.64 (s, 1H, NH); ^13^C NMR (100 MHz, δ ppm DMSO*-d*_6_): δ 13.25, 22.38, 44.38 ppm (aliphatic Cs), δ 122.00, 125.29, 128.12, 128.97, 130.37, 146.36, 146.89 ppm (aromatic and triazole rings Cs), and δ 167.31 ppm (C=S); EI-MS (*m*/*z*, %): 286.57 (M^+^, 63%), 265.20 (100% base peak); Elemental analysis for C_13_H_14_N_6_S (286.36): Calculated/Found: 54.53/54.70 (%C), 4.93/5.01 (%H), 29.35/29.48 (%N), and 11.20/11.27 (%S).

##### 4-Phenyl-5-((4-phenyl-1H-1,2,3-triazol-1-yl)methyl)-2,4-dihydro-3H-1,2,4 -triazole-3-thione (**5b**)

Yellow crystals; yield 86%, mp 278–280 °C; ^1^H NMR (400 MHz, δ ppm DMSO-*d*_6_): 5.60 (s, 2H, CH_2_), 7.27–7.70 (m, 11H, Ar-H, and triazole H), 8.20 (s, 1H, NH); ^13^C NMR (100 MHz, δ ppm DMSO*-d*_6_): δ 44.58 ppm (aliphatic C), δ 122.16, 125.27, 127.96, 128.11, 128.98, 129.50, 129.75, 130.35, 132.83, 146.43, 146.99 ppm (aromatic and triazole rings Cs) and δ 168.76 ppm (C=S); EI-MS (*m*/*z*, %): 334.39 (M^+^, 64%), 153.25 (100% base peak); Elemental analysis for C_17_H_14_N_6_S (334.40): Calculated/Found: 61.06/60.88 (%C), 4.22/4.45 (%H), 25.13/25.49 (%N), and 9.59/9.70 (%S).

#### 3.1.2. General Method for the Synthesis of Compounds **6a**–**i**

To a solution of 1,2,4-triazole-3-thione **4** (5.0 mmol) in absolute ethanol (10 mL), appropriate aryl aldehyde/cyclohexane carbaldehyde/thiophene-2-carbaldehyde (5.0 mmol) and 2 drops of glacial acetic acid were added. The reaction mixture was heated under reflux for 12–15 h before being cooled and put into cold water (40 mL). The precipitated products **6a**–**i** were filtered, dried, and recrystallized from ethanol.

##### (E)-4-((4-Methoxybenzylidene)amino)-5-((4-phenyl-1H-1,2,3-triazol-1-yl)methyl)-2,4-dihydro-3H-1,2,4-triazole-3-thione (**6a**)

Brown crystals; yield 83%; mp 250–252 °C; ^1^H NMR (400 MHz, δ ppm DMSO-*d*_6_): 3.74 (s, 3H, OCH_3_), 5.43 (s, 2H, CH_2_), 6.93 (d, 2H, *J* = 8.80 Hz, Ar-H), 6.94–7.49 (m, 6H, Ar-H and triazole H), 7.51 (d, 2H, *J* = 8.80 Hz, Ar-H), 8.19 (s, 1H, CH=N), 10.39 (s, 1H, NH); ^13^C NMR (100 MHz, δ ppm DMSO*-d*_6_): δ 55.31, 60.23 ppm (aliphatic Cs), δ 114.30, 122.16, 124.80, 127.34, 127.99, 132.63, 136.80, 138.64, 144.16, 149.81, 154.74, 164.35 ppm (aromatic and triazole rings Cs, CH=N), and δ 168.76 ppm (C=S); EI-MS (*m*/*z*, %): 391.05 (M^+^, 70%), 233.09 (100% base peak); Elemental analysis for C_19_H_17_N_7_OS (391.45): Calculated/Found: 58.30/58.49 (%C), 4.38/4.51 (%H), 25.05/25.27 (%N), and 8.19/8.23 (%S).

##### (E)-5-((4-Phenyl-1H-1,2,3-triazol-1-yl)methyl)-4-((3,4,5-trimethoxybenz- ylidene)amino)-2,4-dihydro-3H-1,2,4-triazole-3-thione (**6b**)

Yellow crystals; yield 85%; mp 239–241 °C; ^1^H NMR (400 MHz, δ ppm DMSO-*d*_6_): 3.64 (s, 3H, OCH_3_), 3.77 (s, 6H, 2 OCH_3_), 5.45 (s, 2H, CH_2_), 6.87–7.27 (m, 8H, Ar-H and triazole H), 8.17 (s, 1H, CH=N), 10.39 (s, 1H, NH); ^13^C NMR (100 MHz, δ ppm DMSO*-d*_6_): δ 56.01, 60.23, 64.80 ppm (aliphatic Cs), δ 103.68, 122.16, 126.40, 128.80, 130.36, 132.63, 138.64, 144.12, 146.99, 149.70, 153.25, 164.51 ppm (aromatic and triazole rings Cs, CH=N), and δ 168.76 ppm (C=S); EI-MS (*m*/*z*, %): 451.06 (M^+^, 67%), 299.54 (100% base peak); Elemental analysis for C_21_H_21_N_7_O_3_S (451.51): Calculated/Found: 55.86/56.09 (%C), 4.69/4.87 (%H), 21.72/21.98 (%N) and 7.10/7.04 (%S).

##### (E)-4-((4-Chlorobenzylidene)amino)-5-((4-phenyl-1H-1,2,3-triazol-1-yl) methyl)-2,4-dihydro-3H-1,2,4-triazole-3-thione (**6c**)

Brown crystals; yield 80%; mp 235–237 °C; ^1^H NMR (400 MHz, δ ppm DMSO-*d*_6_): 5.44 (s, 2H, CH_2_), 7.15–7.64 (m, 6H, Ar-H and triazole H), 7.46 (d, 2H, *J* = 8.60 Hz, Ar-H), 7.93 (d, 2H, *J* = 8.60 Hz, Ar-H), 8.23 (s, 1H, CH=N), 10.50 (s, 1H, NH); ^13^C NMR (100 MHz, δ ppm DMSO*-d*_6_): δ 60.23 ppm (aliphatic C), δ 114.30, 122.16, 124.80, 127.34, 127.99, 132.63, 136.80, 138.64, 144.16, 149.81, 154.74, 164.35 ppm, (aromatic and triazole rings Cs, CH=N), and δ 168.76 ppm (C=S); EI-MS (*m*/*z*, %): 395.89 (M^+^, 56%), 231.48 (100% base peak); Elemental analysis for C_18_H_14_ClN_7_S (395.87): Calculated/Found: 54.61/54.78 (%C), 3.56/3.70 (%H), 24.77/24.98 (%N), and 8.10/8.23 (%S).

##### (E)-4-((2,4-Dichlorobenzylidene)amino)-5-((4-phenyl-1H-1,2,3-triazol-1-yl)methyl)-2,4-dihydro-3H-1,2,4-triazole-3-thione (**6d**)

Yellow crystals; yield 82%; mp 236–238 °C; ^1^H NMR (400 MHz, δ ppm DMSO-*d*_6_): 5.52 (s, 2H, CH_2_), 7.14–7.92 (m, 9H, Ar-H and triazole H), 8.68 (s, 1H, CH=N), 10.50 (s, 1H, NH); ^13^C NMR (100 MHz, δ ppm DMSO*-d*_6_): δ 60.23 ppm (aliphatic C), δ 121.34, 123.28, 127.67, 127.96, 129.35, 132.63, 133.12, 134.22, 138.90, 142.22, 149.45, 154.79, 164.75 ppm (aromatic and triazole rings Cs, CH=N), and δ 172.40 ppm (C=S); EI-MS (*m*/*z*, %): 430.20 (M^+^, 57%), 171.31 (100% base peak); Elemental analysis for C_18_H_13_Cl_2_N_7_S (430.31): Calculated/Found: 50.24/50.51 (%C), 3.05/3.23 (%H), 22.78/23.05 (%N), and 7.45/7.68 (%S).

##### (E)-4-((2-Bromobenzylidene)amino)-5-((4-phenyl-1H-1,2,3-triazol-1-yl) methyl)-2,4-dihydro-3H-1,2,4-triazole-3-thione (**6e**)

Yellow crystals; yield 77%; mp 241–243 °C; ^1^H NMR (400 MHz, δ ppm DMSO-*d*_6_): 5.51 (s, 2H, CH_2_), 7.27–7.89 (m, 10H, Ar-H, and triazole H), 8.68 (s, 1H, CH=N), 11.07 (s, 1H, NH); ^13^C NMR (100 MHz, δ ppm DMSO*-d*_6_): δ 60.60 ppm (aliphatic C), δ 120.08, 120.71, 122.76, 124.89, 126.74, 127.95, 129.42, 130.85, 133.05, 133.54, 137.38, 142.20, 149.43, 164.58 ppm (aromatic and triazole rings Cs, CH=N), and δ 172.44 ppm (C=S); EI-MS (*m*/*z*, %): 440.26 (M^+^, 56%), 442.37 (M^+^+2, 57.34%), 220.78 (100% base peak); Elemental analysis for C_18_H_14_BrN_7_S (440.32): Calculated/Found: 49.10/49.37 (%C), 3.20/3.28 (%H), 22.27/22.41 (%N), and 7.28/7.29 (%S).

##### (E)-4-((4-Bromobenzylidene)amino)-5-((4-phenyl-1H-1,2,3-triazol-1-yl) methyl)-2,4-dihydro-3H-1,2,4-triazole-3-thione (**6f**)

Brown crystals; yield 79%; mp 247–249 °C; ^1^H NMR (400 MHz, δ ppm DMSO-*d*_6_): 5.51 (s, 2H, CH_2_), 7.14–7.61 (m, 6H, Ar-H and triazole H), 7.60 (d, 2H, *J* = 8.30 Hz, Ar-H), 7.72, (d, 2H, *J* = 8.30 Hz, Ar-H), 8.28 (s, 1H, CH=N), 11.07 (s, 1H, NH); ^13^C NMR (100 MHz, δ ppm DMSO*-d*_6_): δ 63.21 ppm (aliphatic C), δ 120.78, 122.75, 126.60, 128.71, 130.51, 132.20, 134.44, 137.39, 143.13, 149.97, 155.11, 164.96 ppm (aromatic and triazole rings Cs, CH=N), and δ 176.89 ppm (C=S); EI-MS (*m*/*z*, %): 440.17 (M^+^, 72%), 442.22 (M ^+^ +2, 69.94%), 60.61 (100% base peak); Elemental analysis for C_18_H_14_BrN_7_S (440.32): Calculated/Found: 49.10/49.35 (%C), 3.20/3.42 (%H), 22.27/22.43 (%N), and 7.28/7.43 (%S).

##### (E)-4-((4-Isopropylbenzylidene)amino)-5-((4-phenyl-1H-1,2,3-triazol-1-yl) methyl)-2,4-dihydro-3H-1,2,4-triazole-3-thione (**6g**)

Yellow crystals; yield 78%; mp 238–240 °C; ^1^H NMR (400 MHz, δ ppm DMSO-*d*_6_): 1.16 (d, 6H, *J* = 8.00 Hz, 2CH_3_), 5.22- 5.38 (m, 1H, CH), 5.44 (s, 2H, CH_2_), 7.09–7.47 (m, 6H, Ar-H and triazole H), 7.24 (d, 2H, *J* = 8.20 Hz, Ar-H), 7.49 (d, 2H, *J* = 8.20 Hz, A-H), 8.23 (s, 1H, CH=N), 10.50 (s, 1H, NH); ^13^C NMR (100 MHz, δ ppm DMSO*-d*_6_): δ 23.77, 33.39, 60.23 ppm (aliphatic Cs), δ 122.16, 126.56, 126.78, 127.05, 129.15, 130.36, 132.43, 142.22, 149.73, 149.86, 154.75, 164.43 ppm (aromatic and triazole rings Cs, CH=N), and δ 168.76 ppm (C=S); EI-MS (*m*/*z*, %): 403.46 (M^+^, 70%), 44.04 (100% base peak); Elemental analysis for C_21_H_21_N_7_S (403.51): Calculated/Found: 62.51/62.70 (%C), 5.25/5.43 (%H), 24.30/24.57 (%N), and 7.95/8.03 (%S).

##### (E)-4-((Cyclohexylmethylene)amino)-5-((4-phenyl-1H-1,2,3-triazol-1-yl) methyl)-2,4-dihydro-3H-1,2,4-triazole-3-thione (**6h**)

Brown crystals; yield 77%; mp 207–209 °C; ^1^H NMR (400 MHz, δ ppm DMSO-*d*_6_): 1.10–1.68 (m, 10H, C_5_H_10_), 2.09–2.16 (m, 1H, CH), 5.34 (s, 2H, CH_2_), 7.09–7.44 (m, 7H, Ar-H, triazole H and CH=N), 9.87 (s, 1H, NH); ^13^C NMR (100 MHz, δ ppm DMSO*-d*_6_): δ 25.05, 25.59, 29.98, 60.23 ppm (aliphatic Cs), δ 122.16, 128.20, 132.63, 138.64, 144.16, 149.95, 152.25, 154.74, 164.49 ppm (aromatic and triazole rings Cs, CH=N), and δ 168.76 ppm (C=S); EI-MS (*m*/*z*, %): 367.61 (M^+^, 59%), 341.24 (100% base peak); Elemental analysis for C_18_H_21_N_7_S (367.48): Calculated/Found: 58.83/59.05 (%C), 5.76/5.87 (%H), 26.68/26.90 (%N), and 8.73/8.81 (%S).

##### (E)-5-((4-Phenyl-1H-1,2,3-triazol-1-yl)methyl)-4-((thiophen-2-yl-methyl- ene)amino)-2,4-dihydro-3H-1,2,4-triazole-3-thione (**6i**)

Yellow crystals; yield 78%; mp 244–246 °C; ^1^H NMR (400 MHz, δ ppm DMSO-*d*_6_): 15.48 (s, 2H, CH_2_), 7.03–7.57 (m, 9H, Ar-H and triazole H), 8.51 (s, 1H, CH=N), 10.50 (s, 1H, NH); ^13^C NMR (100 MHz, δ ppm DMSO*-d*_6_): δ 60.23 ppm (aliphatic C), δ 121.34, 123.28, 128.08, 129.48, 131.64, 136.21, 139.74, 142.59, 149.87, 155.11, 164.92 ppm (aromatic and triazole rings Cs, CH=N), and δ 172.40 ppm (C=S); EI-MS (*m*/*z*, %): 367.47 (M^+^, 63%), 79.87 (100% base peak); Elemental analysis for C_16_H_13_N_7_S_2_ (367.45): Calculated/Found: 52.30/52.53 (%C), 3.57/3.71 (%H), 26.68/26.85 (%N), and 17.45/17.39 (%S).

### 3.2. Biology

#### 3.2.1. Cell Viability Assay

To explore the effect of **5a**,**b**, and **6a**–**i** hybrids on normal cell lines, a cell viability assay was performed using the MCF-10A (human mammary gland epithelial) cell line [31,32]. See Appendix SA.

#### 3.2.2. Antiproliferative Assay

The antiproliferative effect of hybrids **5a**,**b**, and **6a**–**i** were investigated against four cancer cells [33,34]: A-549 (epithelial cancer cell line), MCF-7 (breast cancer cell line), Panc-1 (pancreas cancer cell line), and HT-29 (colon cancer cell line). The reference drug was Erlotinib. See Appendix SA.

#### 3.2.3. Aromatase Inhibitory Assay

Compounds **5a**, **5b**, and **6a**–**i** were tested for inhibitory action against aromatase as a potential target for their antiproliferative activity [36]. See Appendix SA (Supplementary Material).

## 4. Conclusions

Eleven new 1,2,3-triazole/1,2,4-triazole hybrids (**5a**, **5b**, and **6a**–**i**) were synthesized and tested for antiproliferative efficacy against four cancer cell lines. Compounds **6a** and **6b** were the most effective derivatives against the four cancer cell lines examined, with GI_50_ values comparable to Erlotinib. Compounds **5a**, **5b**, and **6a**–**i** were tested for inhibitory action against aromatase as a potential target for their antiproliferative activity. The results of this assay are consistent with those of the antiproliferative assay, in which the most potent antiproliferative derivatives, compounds **6a** and **6b**, were the most potent aromatase inhibitors, more potent than the reference Ketoconazole but less potent than Letrozole. Docking studies demonstrated that all the compounds tested fit the aromatase CP450 binding site well. Following optimization, the 1,2,3-triazole/1,2,4-triazole hybrids **6a**–**c** are unique prospective targeted anticancer aromatase inhibitors that may contribute efficiently to cancer chemotherapy.

## Figures and Tables

**Table 1:** Antiproliferative activity of tested compounds **5a, 5b**, **6a**–**i**, and **Erlotinib**.

Comp.	Cell Viability %	Antiproliferative Activity IC_50_ ± SEM (nM)				
		A-549	MCF-7	Panc-1	HT-29	Average IC_50_(GI_50_)
**5a**	90	>120	>120	>120	>120	>120
**5b**	92	>120	>120	>120	>120	>120
**6a**	92	38 ± 3	40 ± 4	38 ± 3	42 ± 4	40
**6b**	91	34 ± 3	36 ± 3	34 ± 3	36 ± 3	35
**6c**	87	69 ± 6	74 ± 7	72 ± 6	74 ± 6	72
**6d**	89	84 ± 8	86 ± 8	82 ± 8	84 ± 8	84
**6e**	90	78 ± 7	80 ± 8	77 ± 7	80 ± 7	79
**6f**	92	112 ± 10	117 ± 10	110 ± 10	112 ± 10	113
**6g**	91	103 ± 10	107 ± 10	100 ± 10	104 ± 10	104
**6h**	87	95 ± 9	101 ± 9	97 ± 9	98 ± 9	98
**6i**	92	89 ± 8	94 ± 9	87 ± 8	89 ± 8	90
**Erlotinib**	ND	30 ± 3	40 ± 3	30 ± 3	30 ± 3	33

**Table 2:** Aromatase inhibitory activity of compounds **5a**, **5b**, and **6a**–**i**.

Comp.	IC_50_ ± SEM (µM)
**5a**	>12.5
**5b**	>12.5
**6a**	0.12 ± 0.01
**6b**	0.09 ± 0.01
**6c**	0.40 ± 0.04
**6d**	>12.5
**6e**	>12.5
**6f**	>12.5
**6g**	>12.5
**Ketoconazole**	2.6 ± 0.20
**Letrozole**	0.002 ± 0.0002

**Table 3:** Caspase-3, Caspase-8, Baz, and Bcl2 levels of compounds **6a**–**c**.

Compd. No.	Caspase-3		Caspase-8		Bax		Bcl-2	
	Conc (Pg/mL)	Fold Change	Conc (ng/mL)	Fold Change	Conc (Pg/mL)	Fold Change	Conc (ng/mL)	Fold Reduction
**6a**	495 ± 4	7.5	2.05	23	295	33	0.80	6
**6b**	525 ± 5	8.0	2.30	25	320	35	0.70	7
**6c**	325 ± 3	5.0	ND	ND	ND	ND	ND	ND
**Staurosporine**	465 ± 4	7.0	1.85	21	288	32	1.00	5
**Control**	65	1.0	0.09	1	9	1	5.00	1

**Table 4:** Modeling studies of tested active compounds **6a**–**c** with aromatase CP450 enzyme.

Compd. No.	The Score (kcal/mole)	Types of Interactions		
		Hydrogen Bond (Distance Å)	π–π Interactions (Arene-H or Cation) (Distance Å)	Hydrophobic Interactions
**Androstenedione**	−8.32	C=O …Ala306 (3.45 Å) C=O… Arg115 (3.44 Å) C=O… Met374 (2.84 Å) C=O… Ser314 (2.54 Å) C=O… Met311 (3.45 Å)	----	Thr310, Leu477, Ile133, Val370, Trp224, Cys437, Arg435, Arg145, Phe430
**6a**	−9.46	S…Arg115, Arg375 & Gly436 (3.79, 4.49 & 3.93 Å) N….Arg115 (3.30 Å)	Val370—Triazole ring (3.74 Å)	Ile133, Cys437, Phe430, Ala306, Ala438, Val373
**6b**	−9.94	S…Arg115, Arg435 & Ile133 (3.19, 4.40 & 3.86 Å) N….Arg115 (3.01 Å) N of triazole ring….Thr310 (3.49 Å)	----	Ala306, Leu477, Cys437, Trp224, Val370, Leu152
**6c**	−9.18	S…Thr310 (4.14 Å) N of triazole ring….Arg115 (3.26 Å)	Cys437—Triazole ring (3.81 Å)	Ile133, Phe430, Met311, Ala306, Val373, Ala438, Val370

## Data Availability

Data will be available upon request from the authors.

## Supplementary Materials

The following supporting information can be downloaded at: https://www.mdpi.com/article/10.3390/molecules28207092/s1.

## Sample Availability

Samples are available from the authors.
