# Supplementary material for: New 1,2,3-Triazole/1,2,4-triazole Hybrids as Aromatase Inhibitors: Design, Synthesis, and Apoptotic Antiproliferative Activity
Source: Molecules. 2023 Oct 14;28(20):7092. doi: 10.3390/molecules28207092 (PMC10609154; doi:10.3390/molecules28207092)

Design, synthesis, and antiproliferative action of new 1,2,3-triazole/1,2,4-triazole hybrids as aromatase inhibitors

Mohamed T-E Maghraby[1,2], Tahani Mazyad Almutairi[3], S. Bräse[4*], Ola I. A. Salem[1], Bahaa G. M. Youssif[1*], Mahmoud M. Sheha[5,6]

[1]Department of Pharmaceutical Organic Chemistry, Faculty of Pharmacy, Assiut University, Assiut 71526, Egypt; [2]Department of Pharmaceutical Chemistry, Faculty of Pharmacy, New Valley University, Egypt; [3]Department of Chemistry, College of Science, King Saud University, Riyadh 11451, Saudi Arabia; [4]Institute of Biological and Chemical Systems, IBCS-FMS, Karlsruhe Institute of Technology, 76131 Karlsruhe, Germany; [5]Department of Medicinal Chemistry, Faculty of Pharmacy, Assiut University, Assiut; [6]Department of Pharmaceutical Chemistry, Faculty of Pharmacy, Sphinx University, Assiut, Egypt.

**To whom correspondence should be addressed:*

Bahaa G. M. Youssif, Ph.D. Pharmaceutical Organic Chemistry Department, Faculty of Pharmacy, Assiut University, Assiut 71526, Egypt.

Tel.: (002)-01098294419

E-mail address: bgyoussif2@gmail.com, bahaa.youssif@pharm.aun.edu.eg

Stefan Bräse

Institute of Biological and Chemical Systems, IBCS-FMS, Karlsruhe Institute of Technology, 76131 Karlsruhe, Germany. E-mail: braese@kit.edu

A. ^{1}H NMR, ^{13}C NMR and EI-mass of compound 5a:

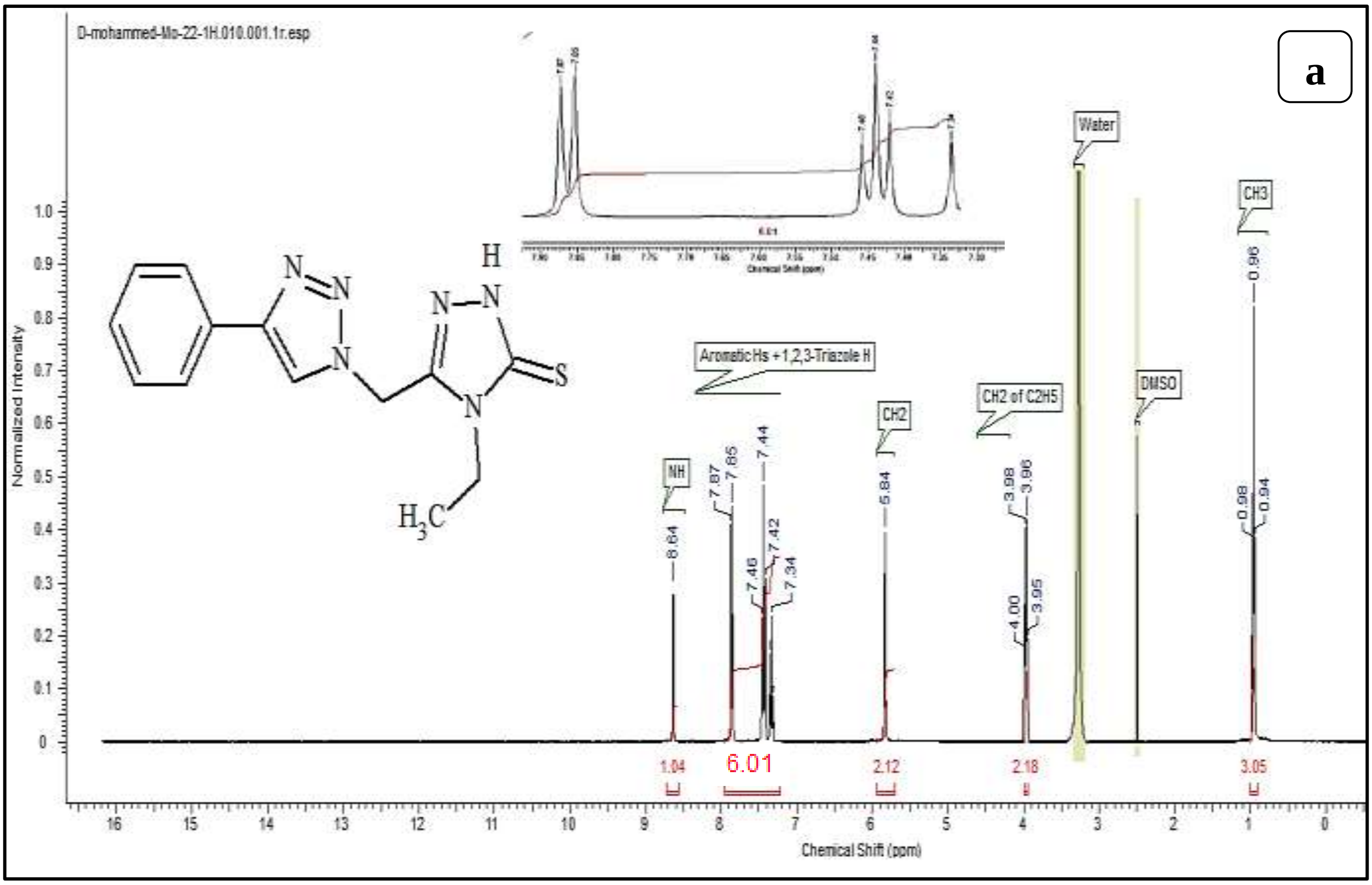

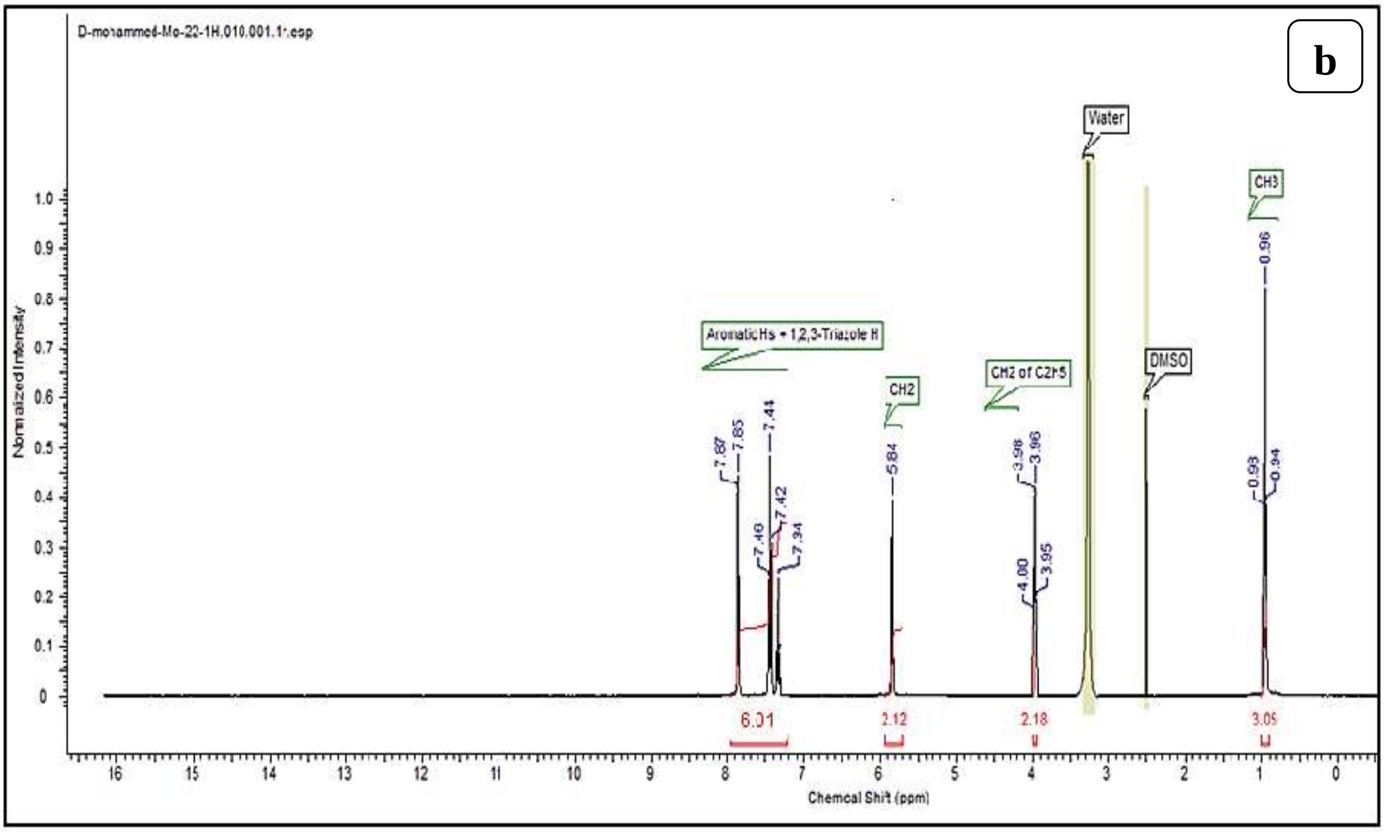

Figure S1: ^{1}H NMR spectrum (400 MHz) of compound **5a** in DMSO-d_6:

(a) before D_2O, (b) after D_2O.

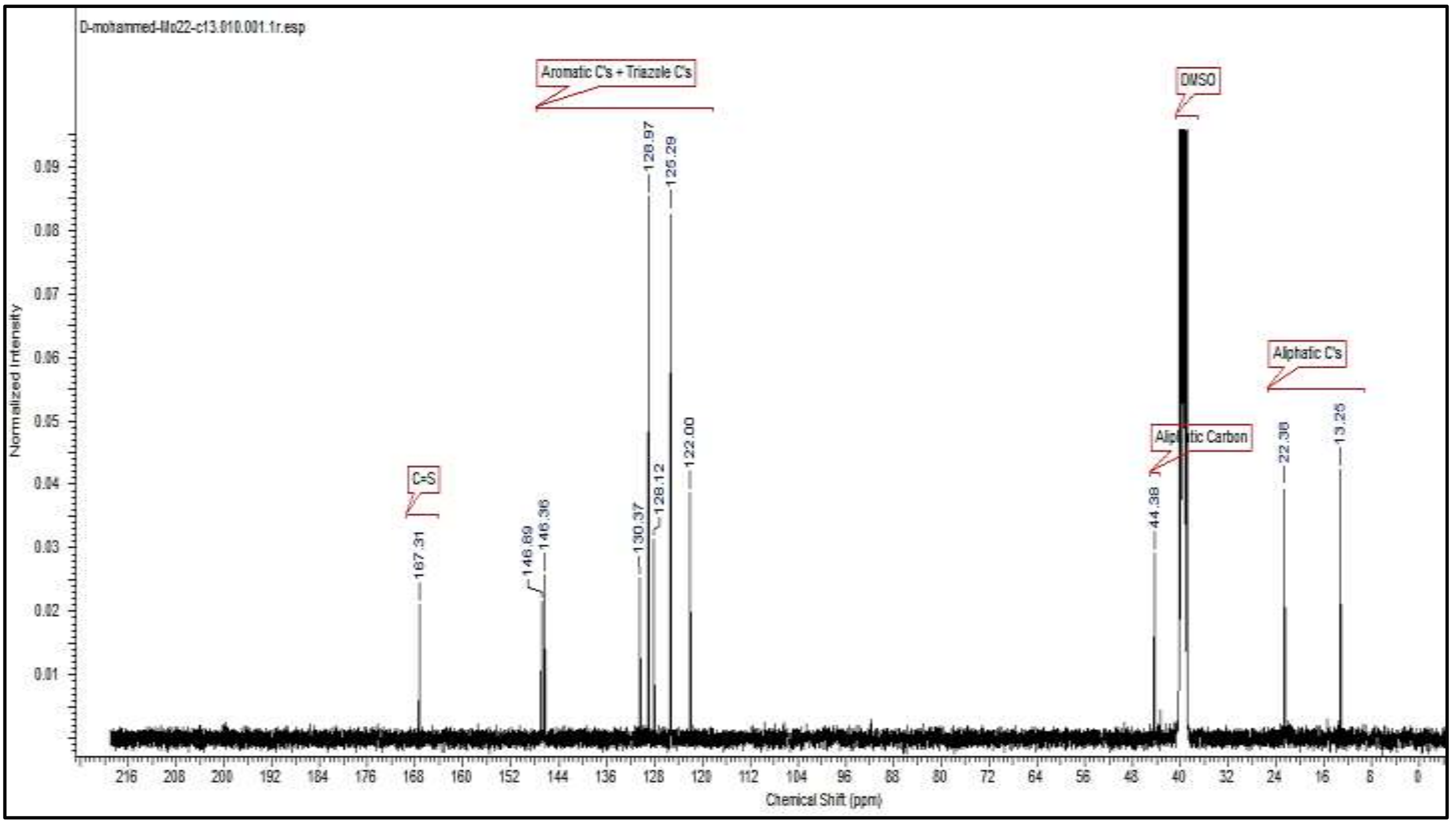

Figure S2: ^{13}C NMR spectrum (100 MHz) of compound **5a** in DMSO-d_6.

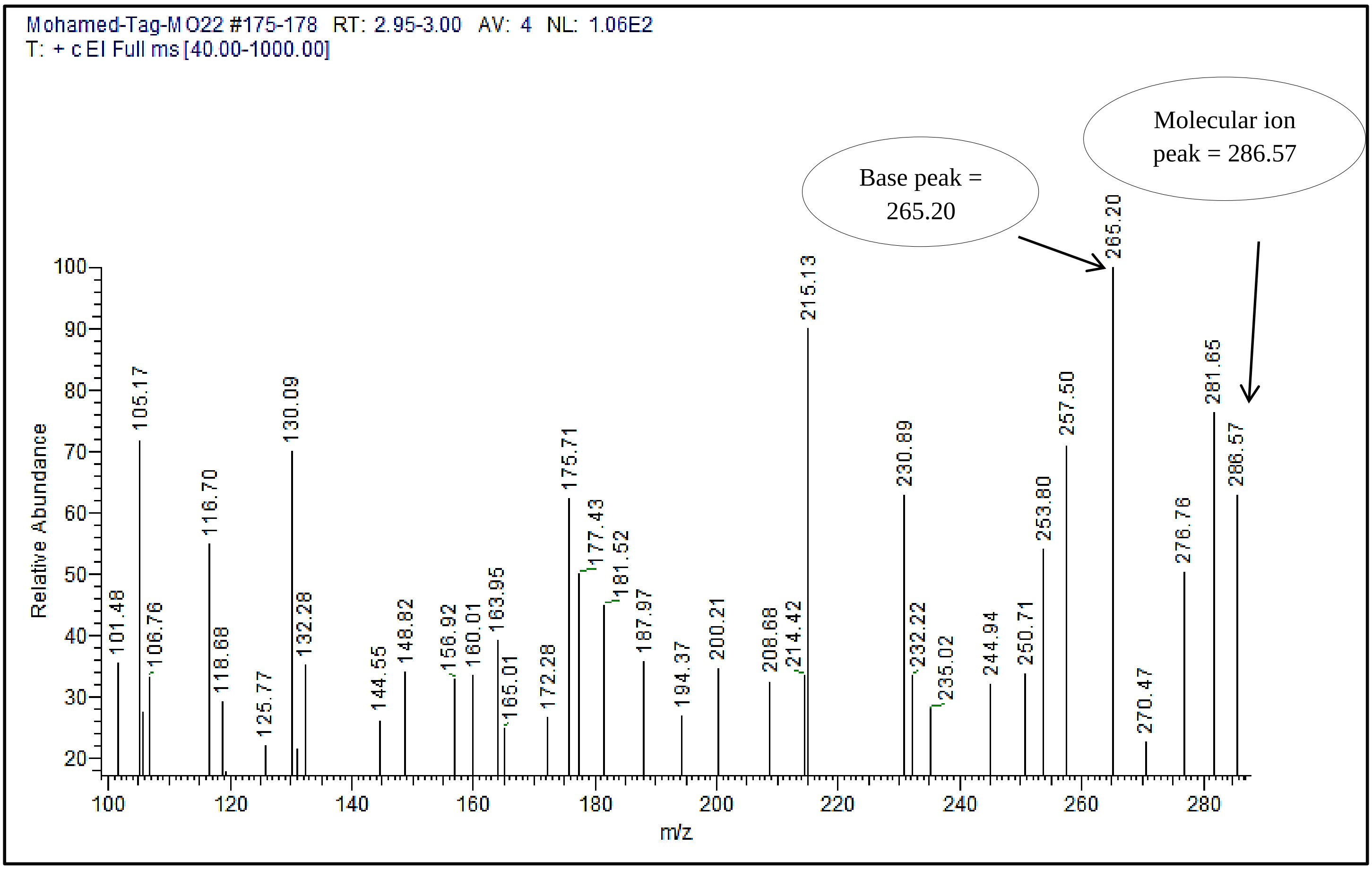

Figure S3: EI-Mass spectrum of compound **5a.**

B. ^{1}H NMR, ^{13}C NMR and EI-mass of compound 5b:

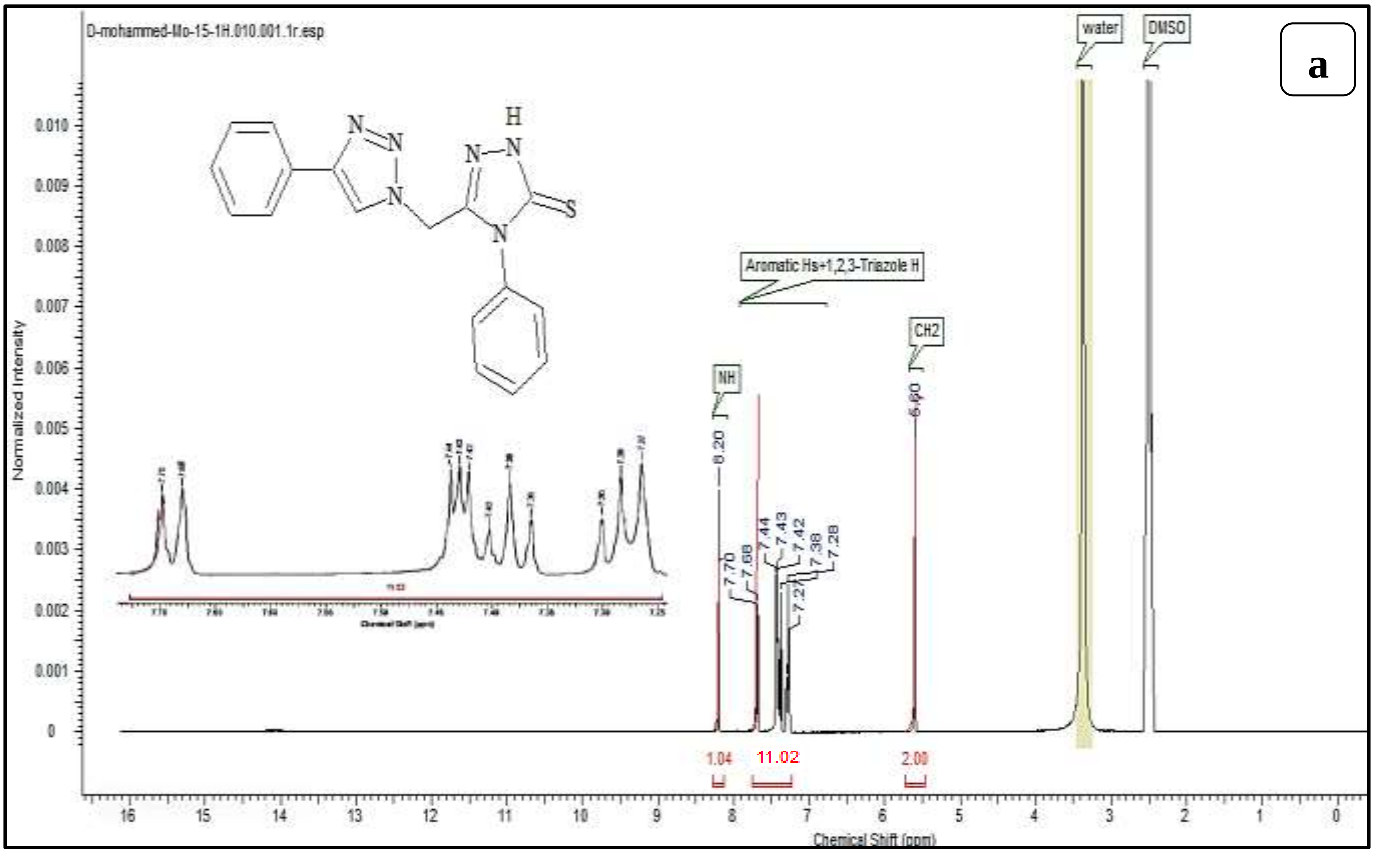

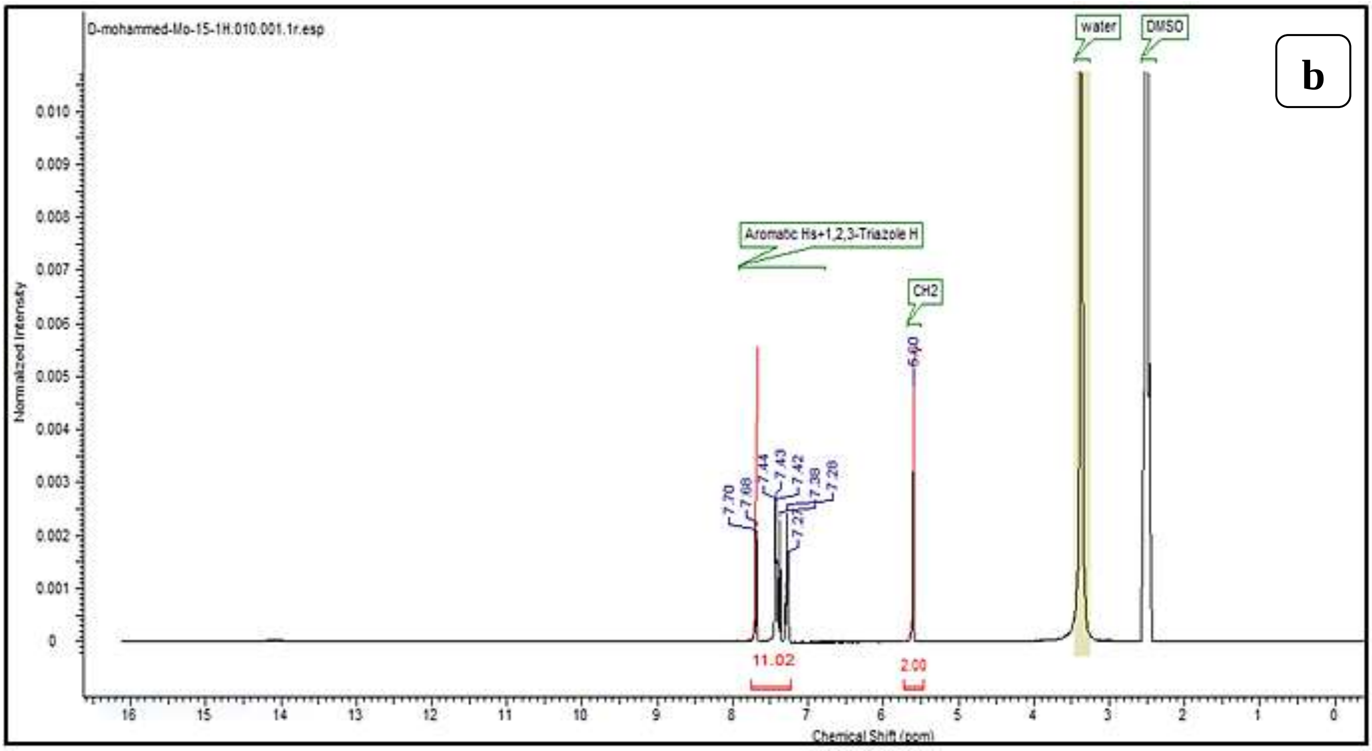

Figure S4: ^{1}H NMR spectrum (400 MHz) of compound **5b** in DMSO-d_6:

(a) before D_2O, (b) after D_2O.

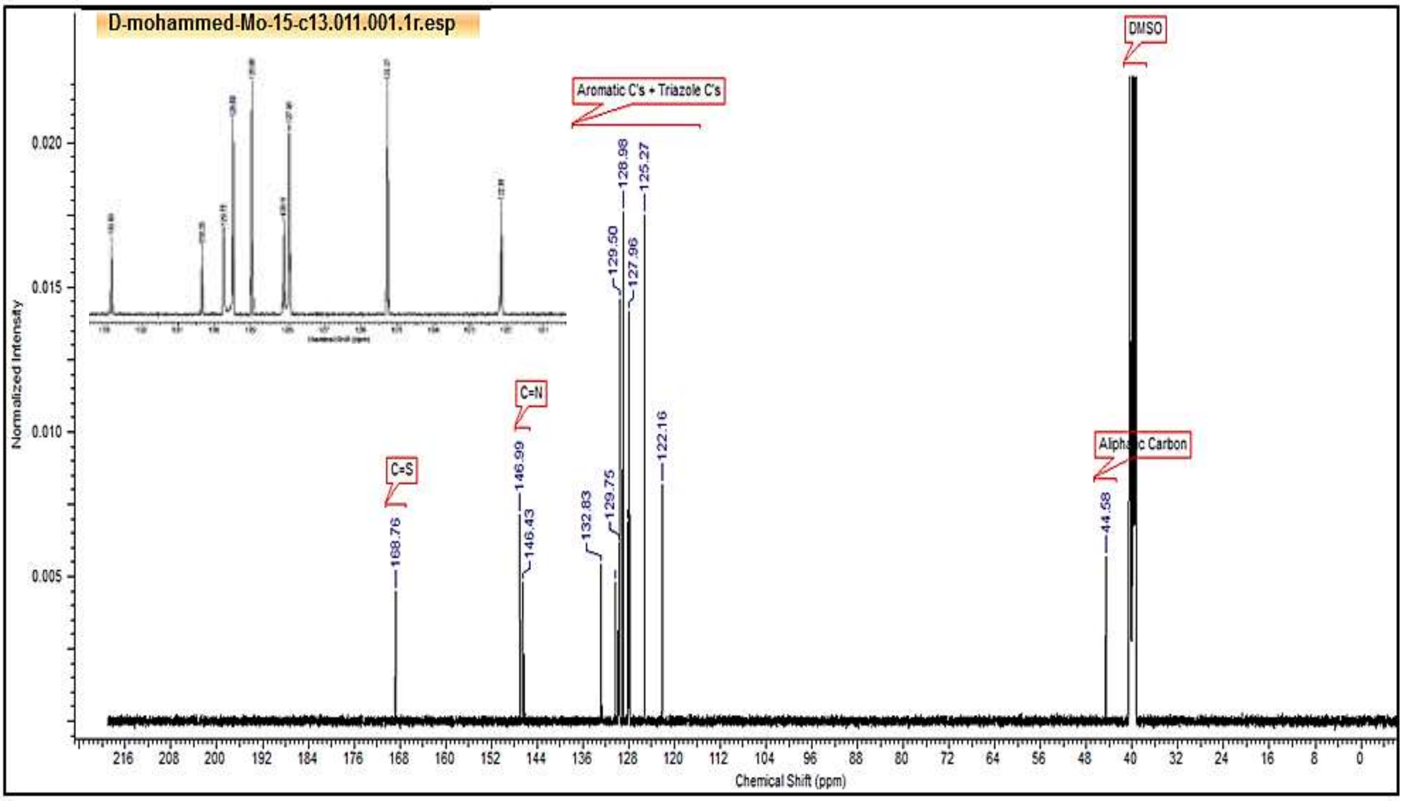

Figure S5: ^{13}C NMR spectrum (100 MHz) of compound **5b** in DMSO-d_6.

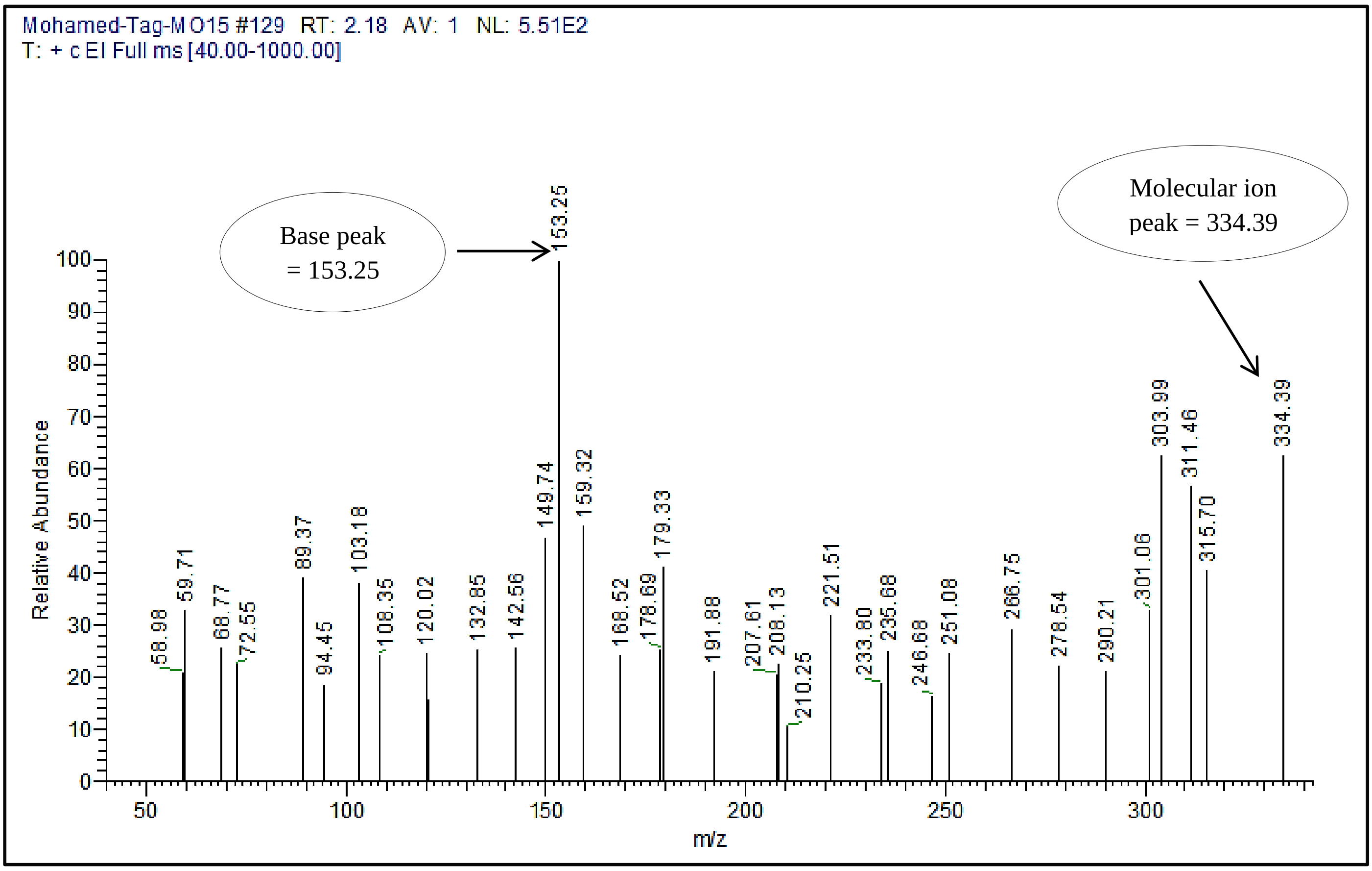

Figure S6: EI-Mass spectrum of compound **5b.**

C. ^{1}H NMR, ^{13}C NMR and EI-mass of compound 6a:

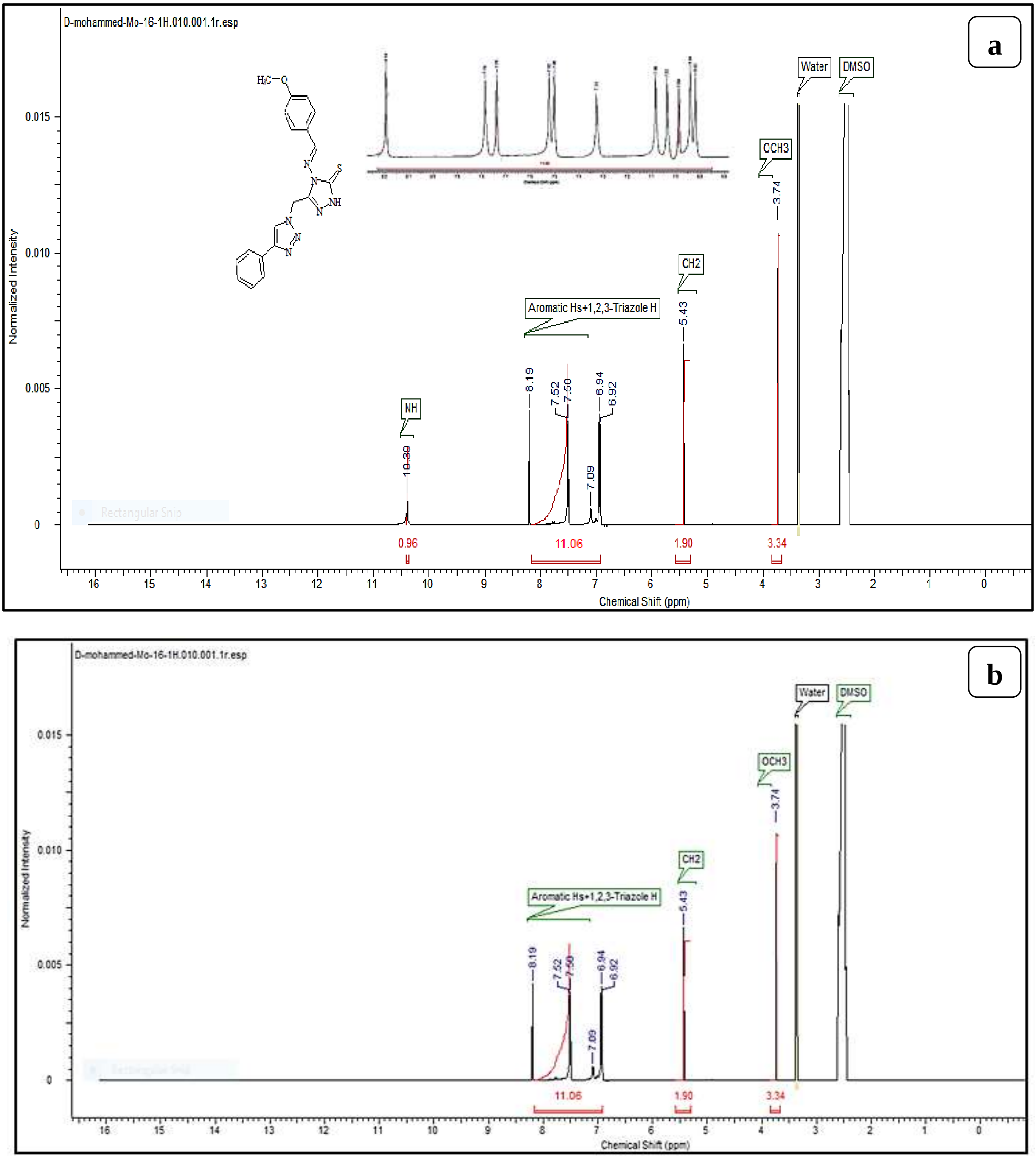

Figure S7: ^{1}H NMR spectrum (400 MHz) of compound **6a** in DMSO-d_6:
(a) before D_2O, (b) after D_2O.

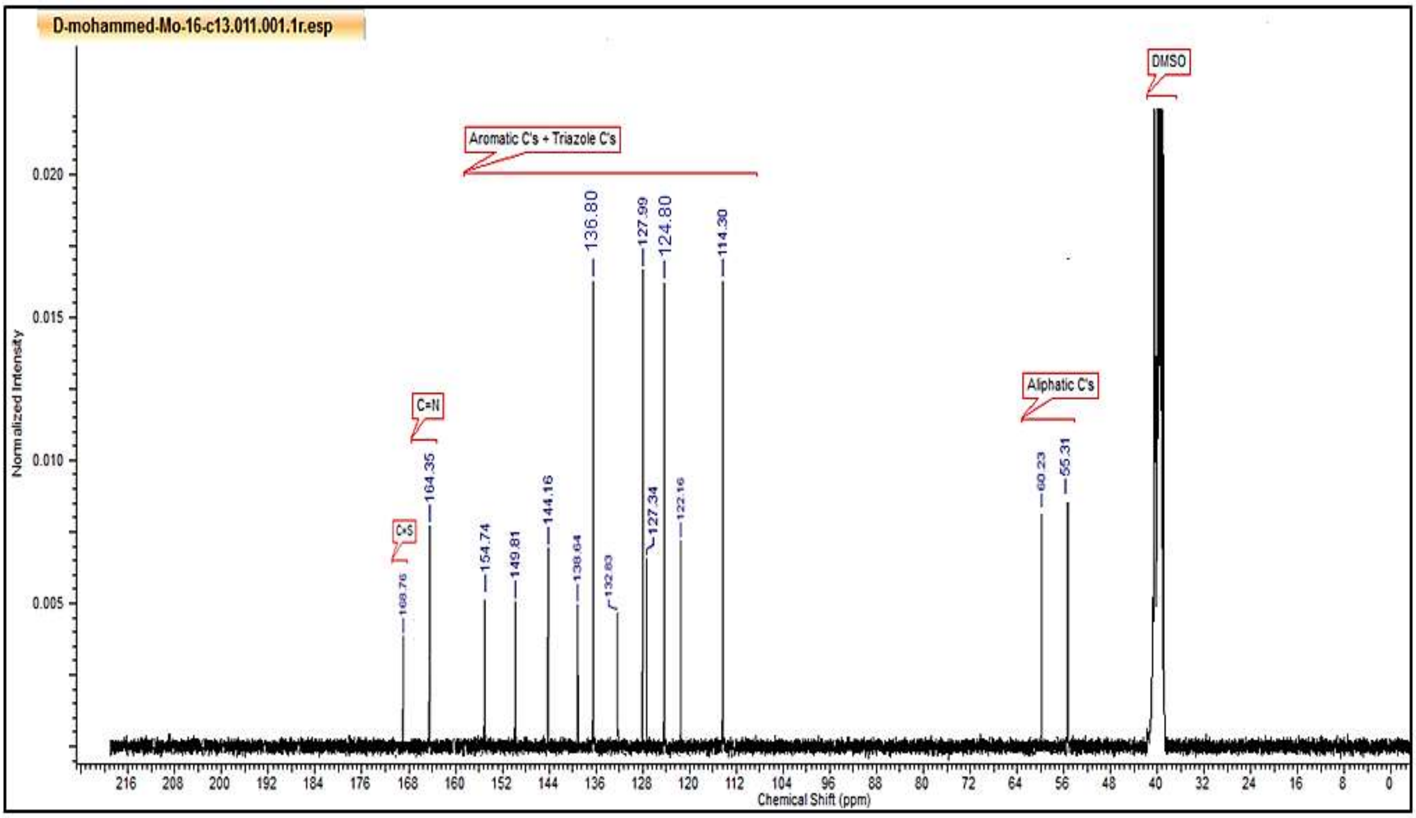

Figure S8: ^{13}C NMR spectrum (100 MHz) of compound **6a** in DMSO-d_6

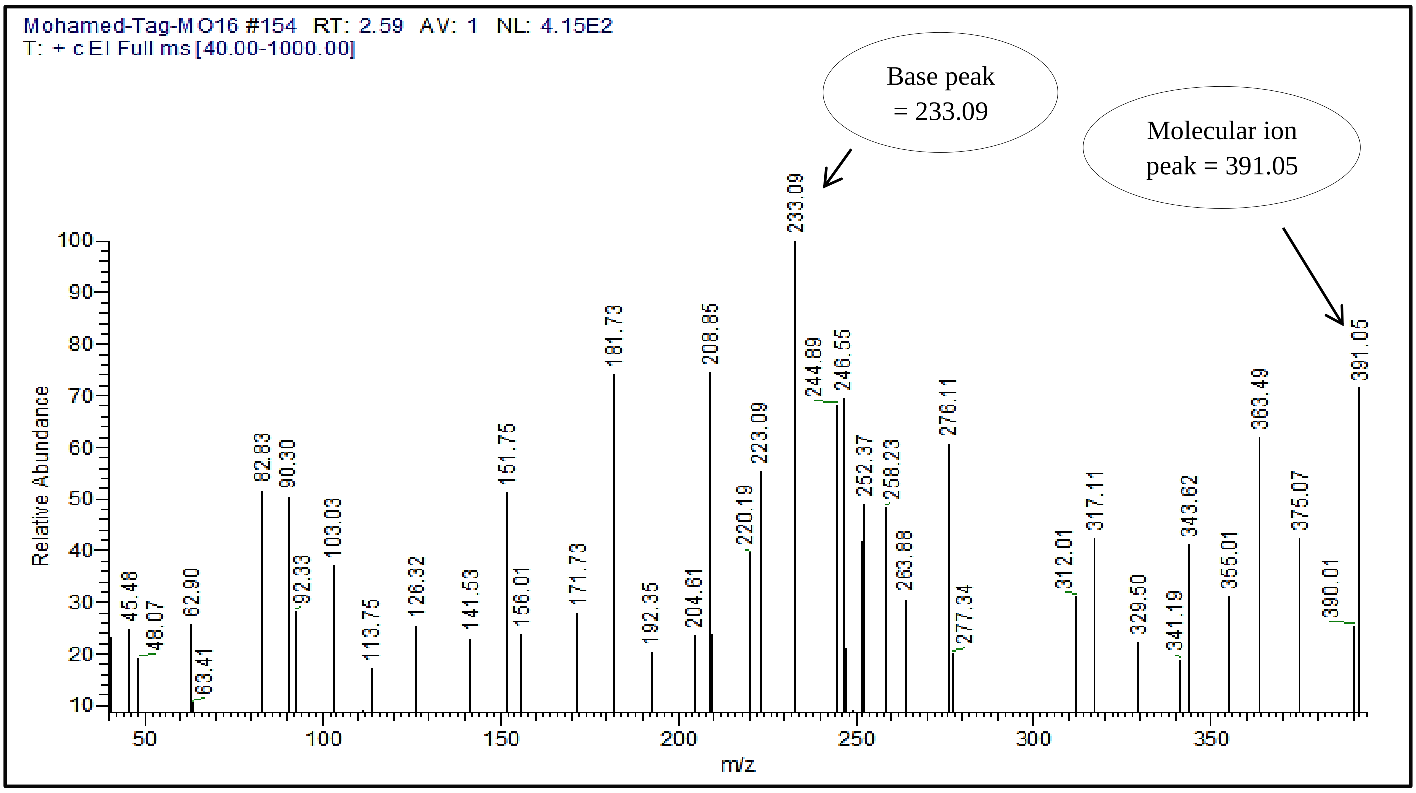

Figure S9: EI-Mass spectrum of compound **6a.**

D. ^{1}H NMR, ^{13}C NMR and EI-mass of compound 6b:

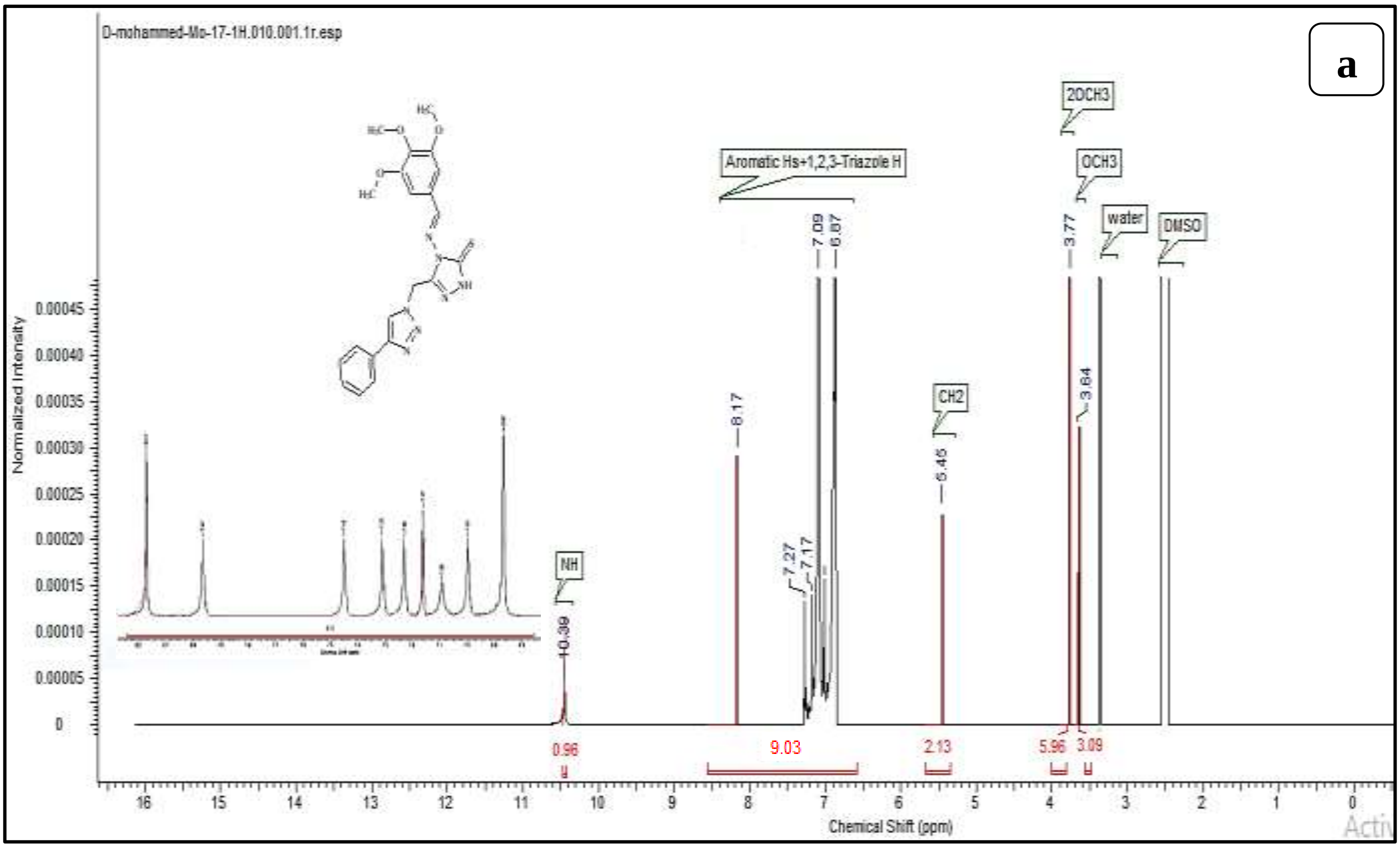

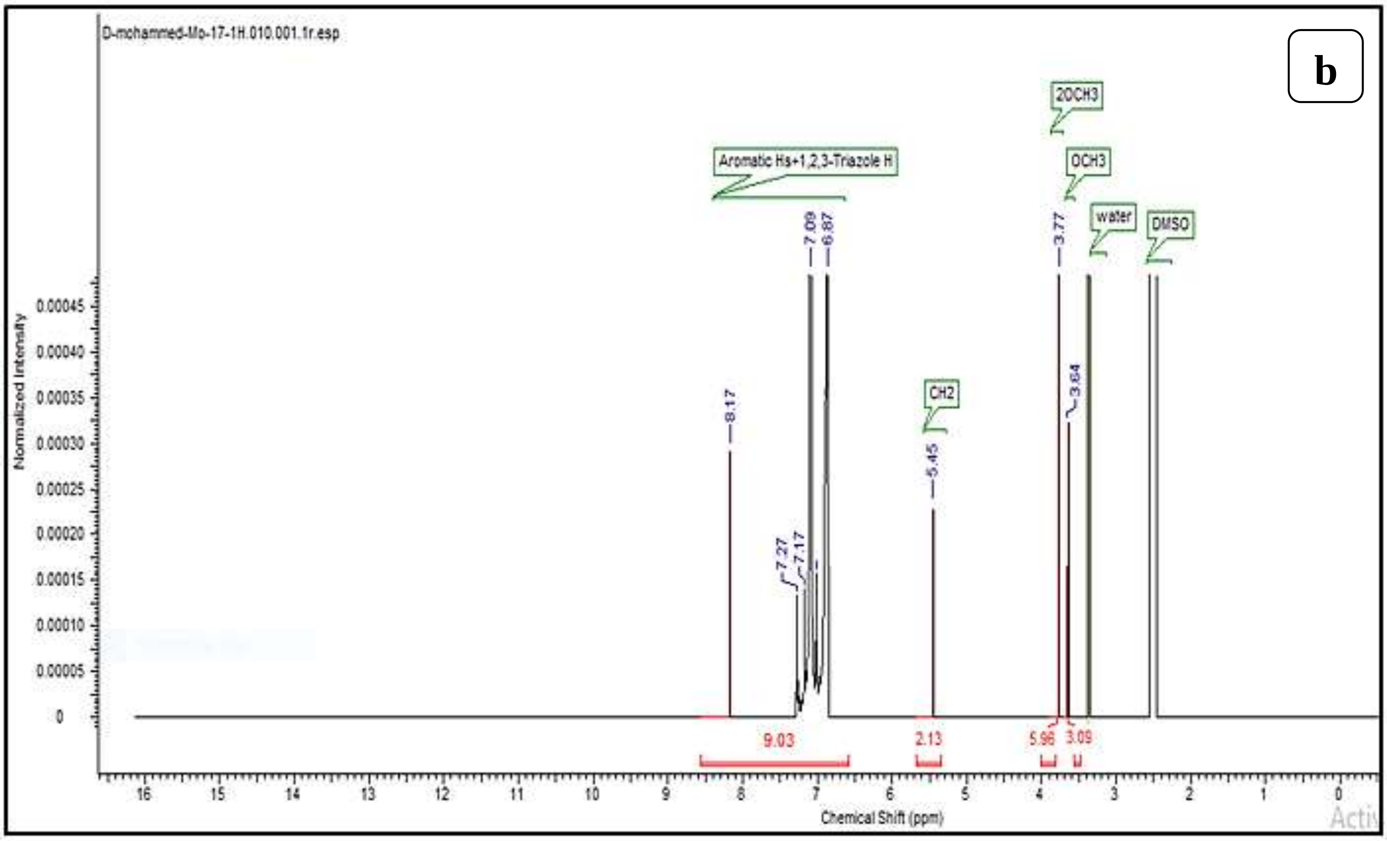

Figure S10: ^{1}H NMR spectrum (400 MHz) of compound **6b** in DMSO-d_6:

(a) before D_2O, (b) after D_2O.

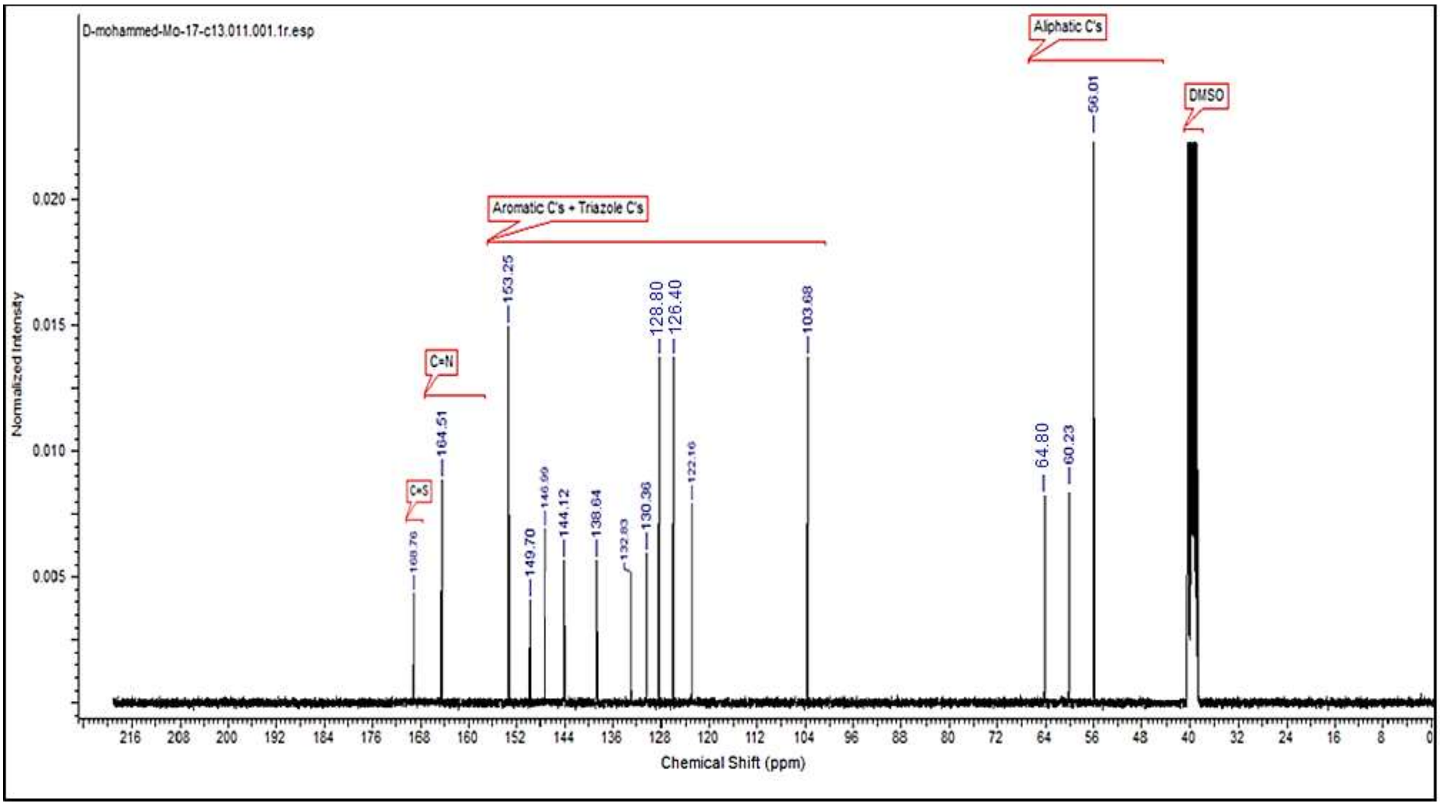

Figure S11: ^{13}C NMR spectrum (100 MHz) of compound **6b** in DMSO-d_6

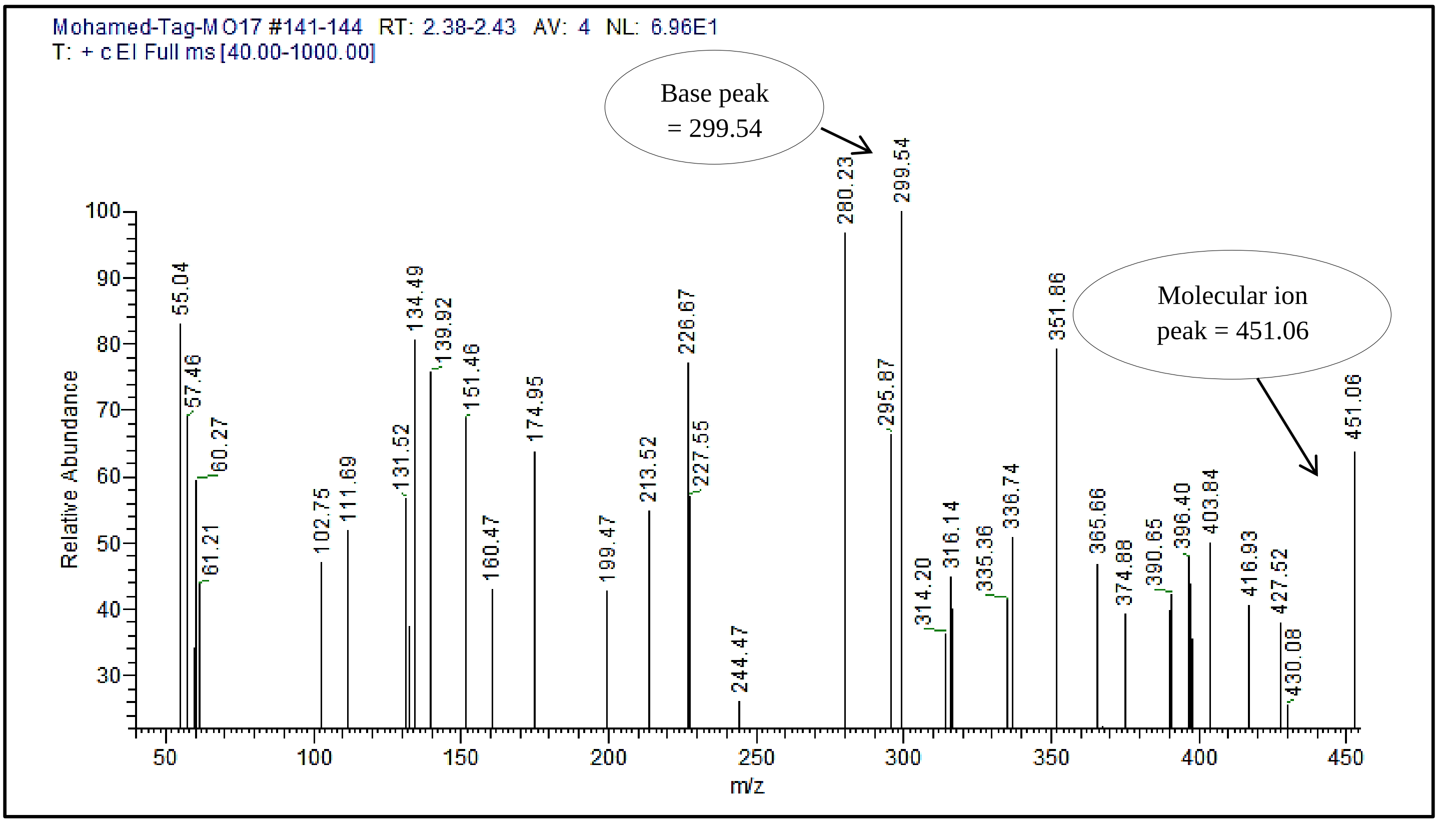

Figure S12: EI-Mass spectrum of compound **6b.**

E. ^{1}H NMR, ^{13}C NMR and EI-mass of compound 6c:

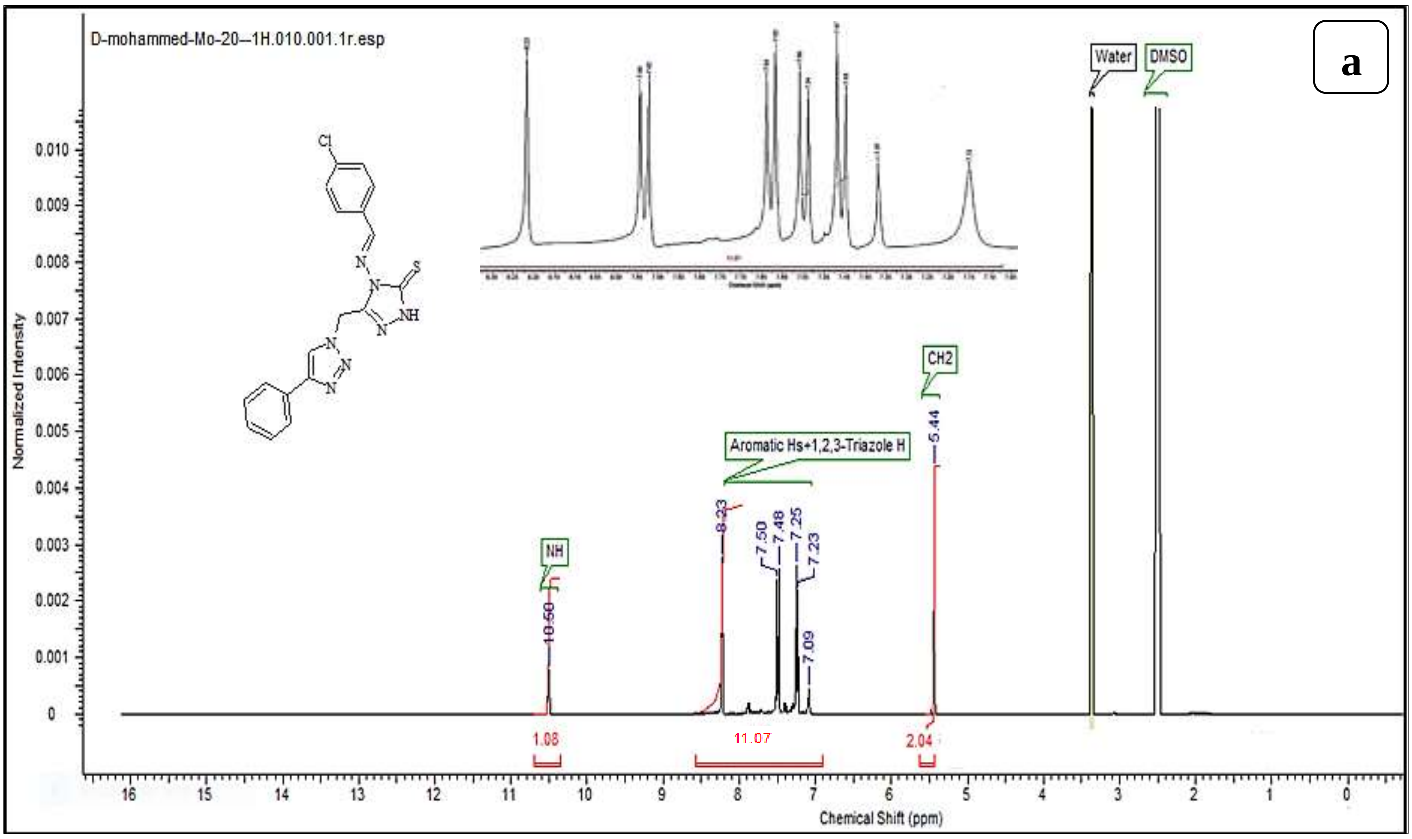

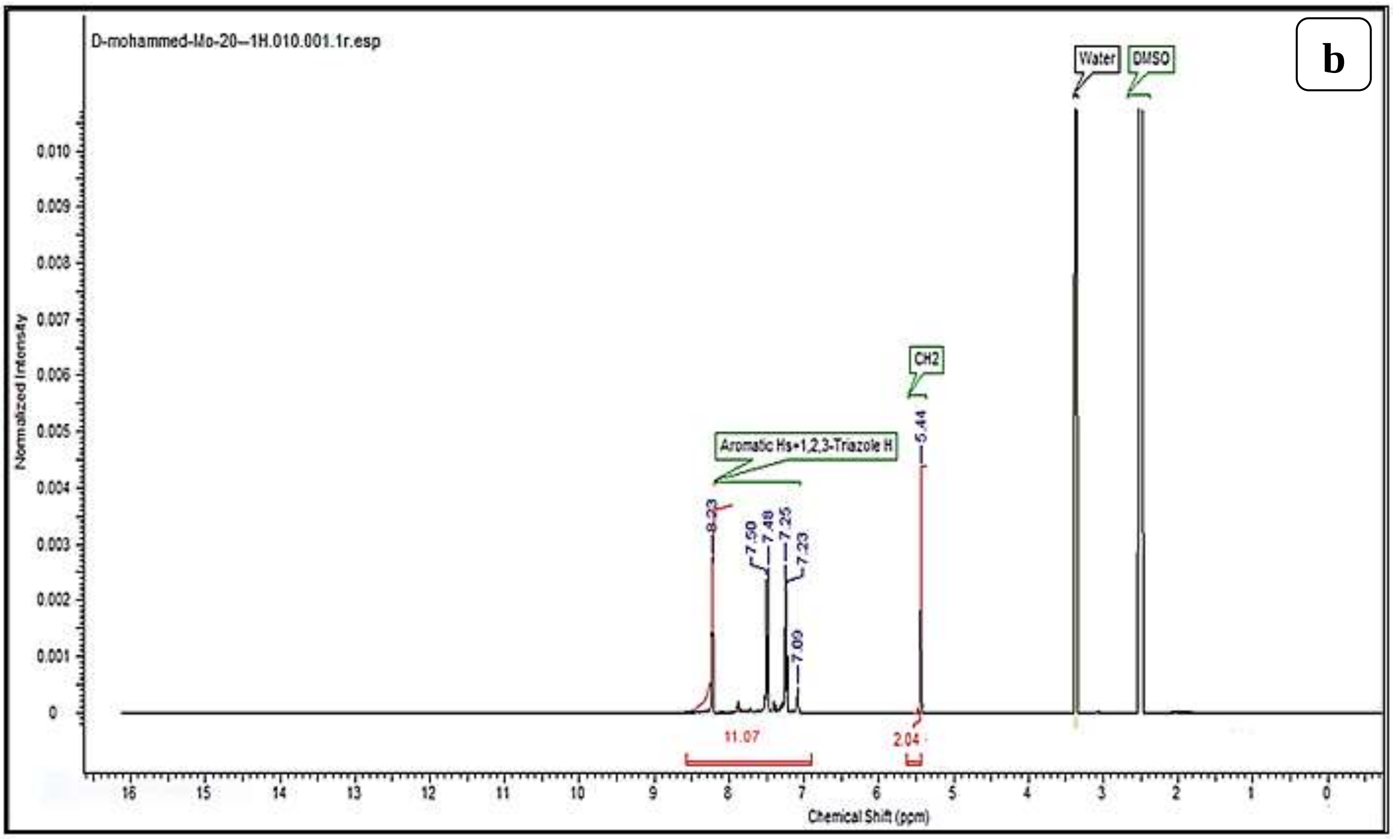

Figure S13: ^{1}H NMR spectrum (400 MHz) of compound **6c** in DMSO-d_6:

(a) before D_2O, (b) after D_2O.

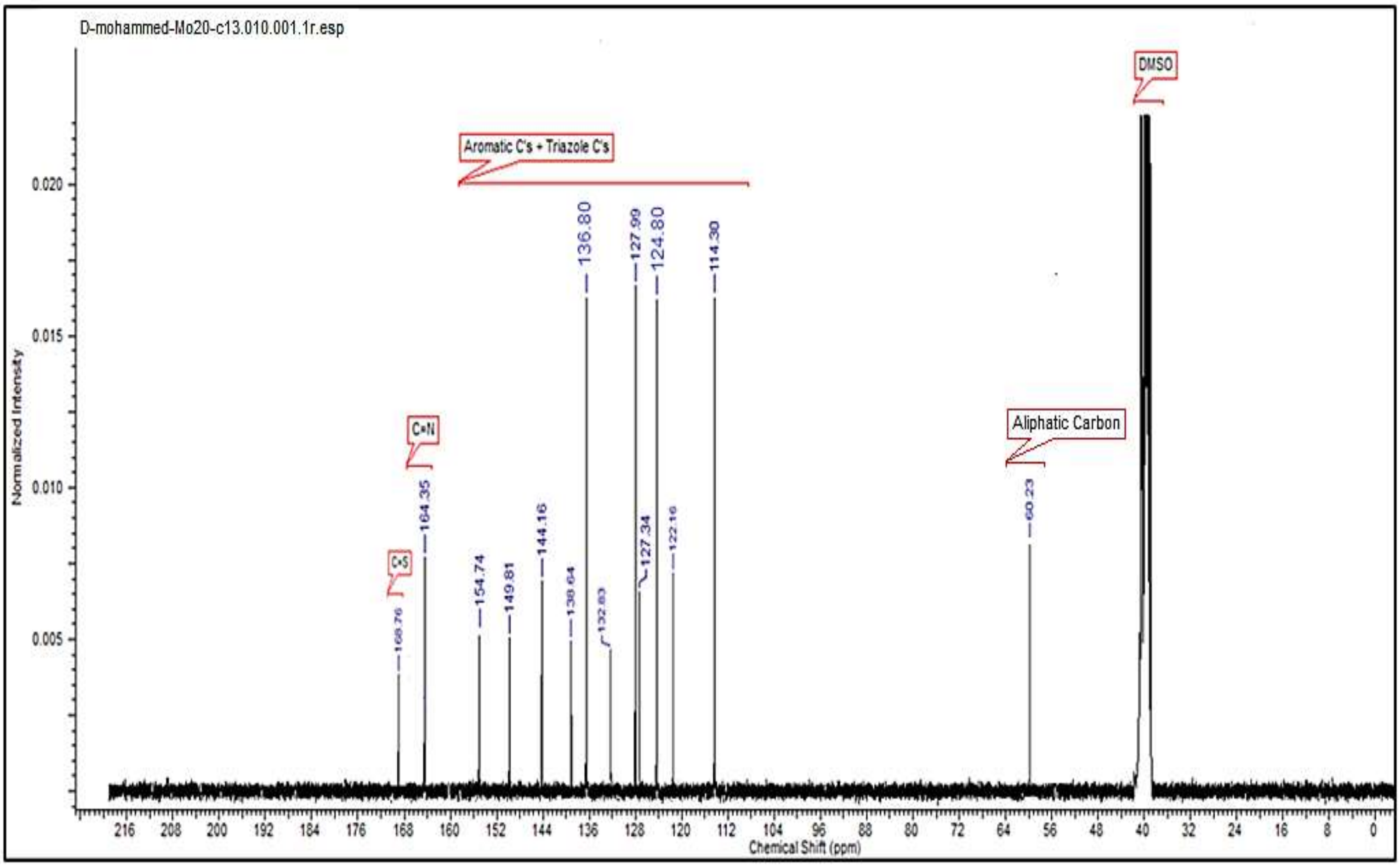

Figure S14: ^{13}C NMR spectrum (100 MHz) of compound **6c** in DMSO-d_6

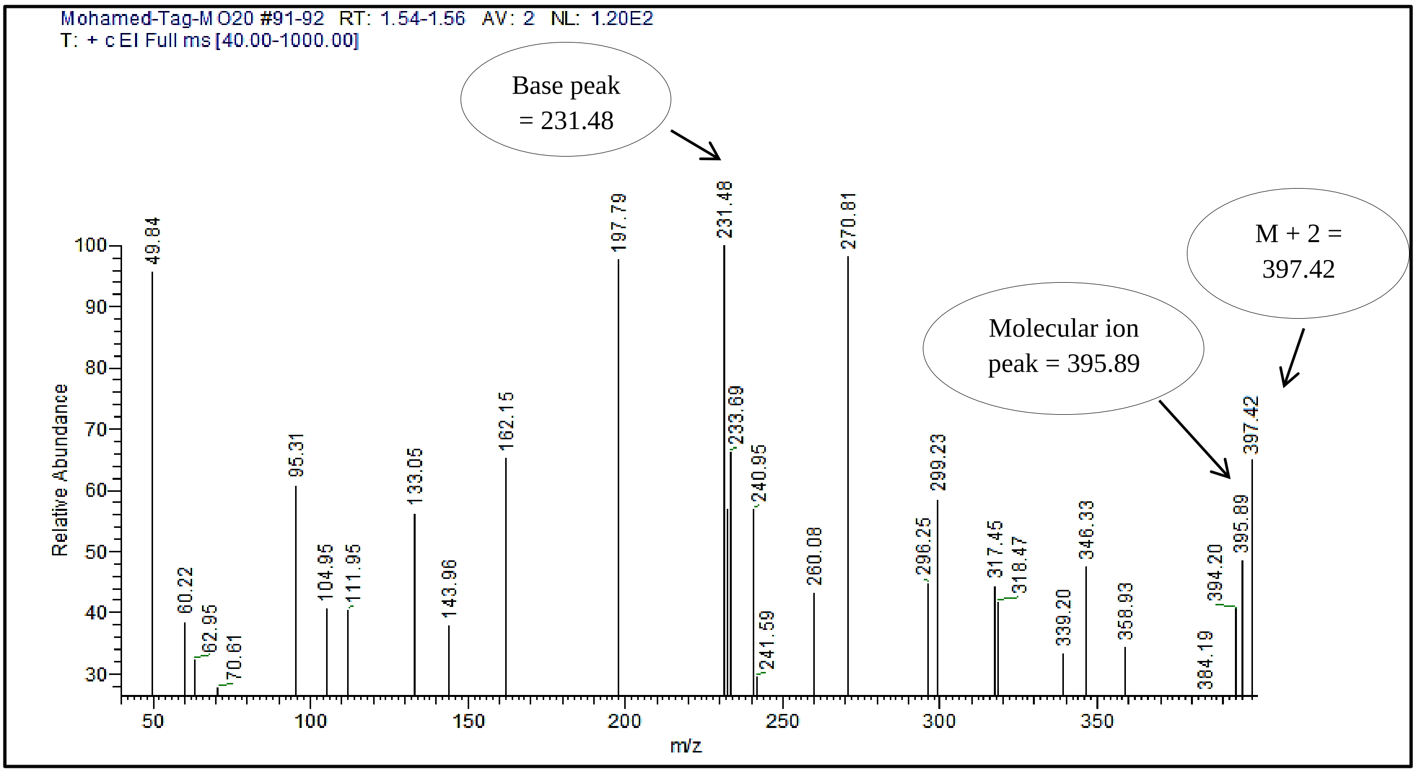

Figure S15: EI-Mass spectrum of compound **6c.**

F. ^{1}H NMR, ^{13}C NMR and EI-mass of compound 6d:

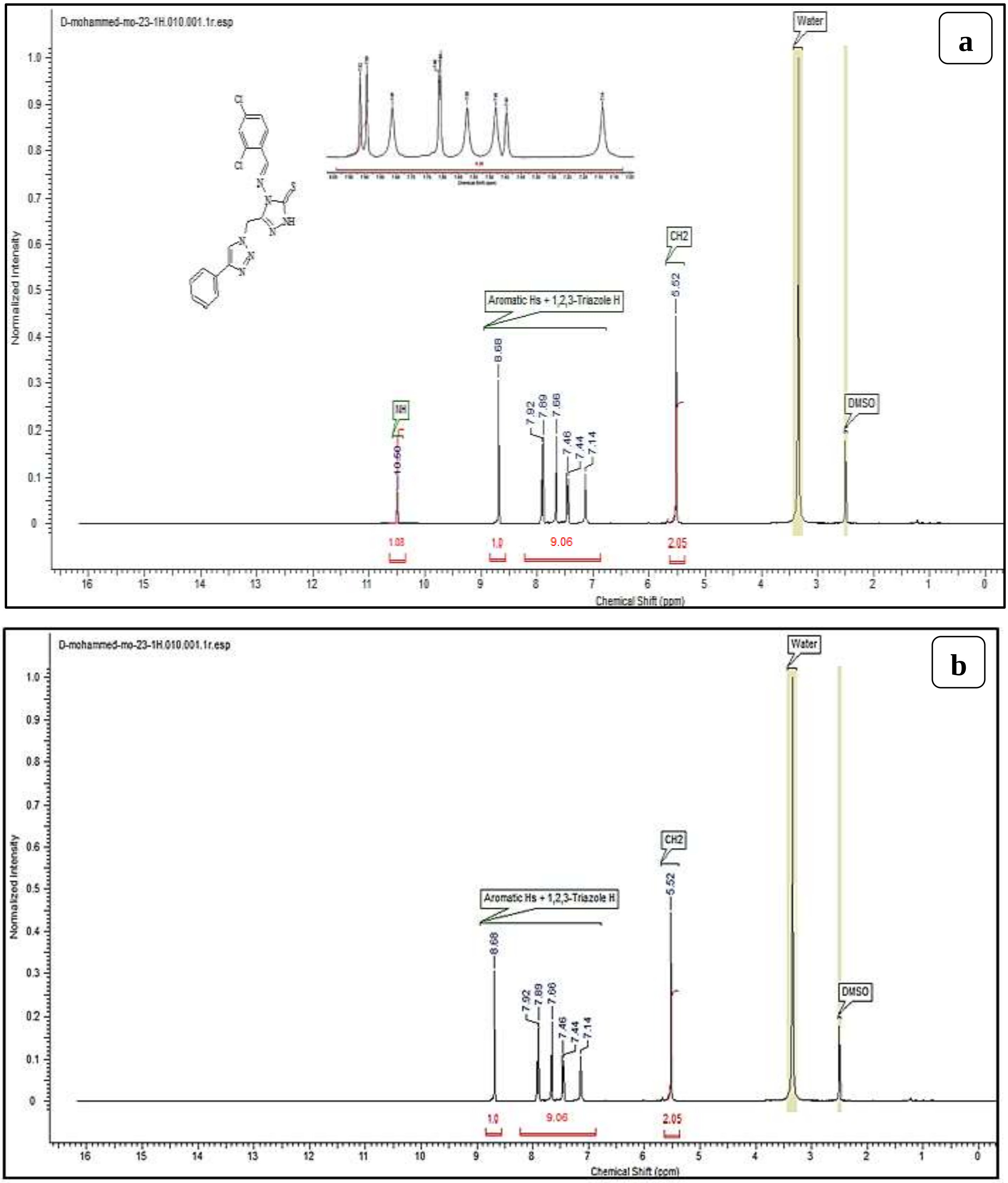

Figure S16: ^{1}H NMR spectrum (400 MHz) of compound **6d** in DMSO-d_6:

(a) before D_2O, (b) after D_2O.

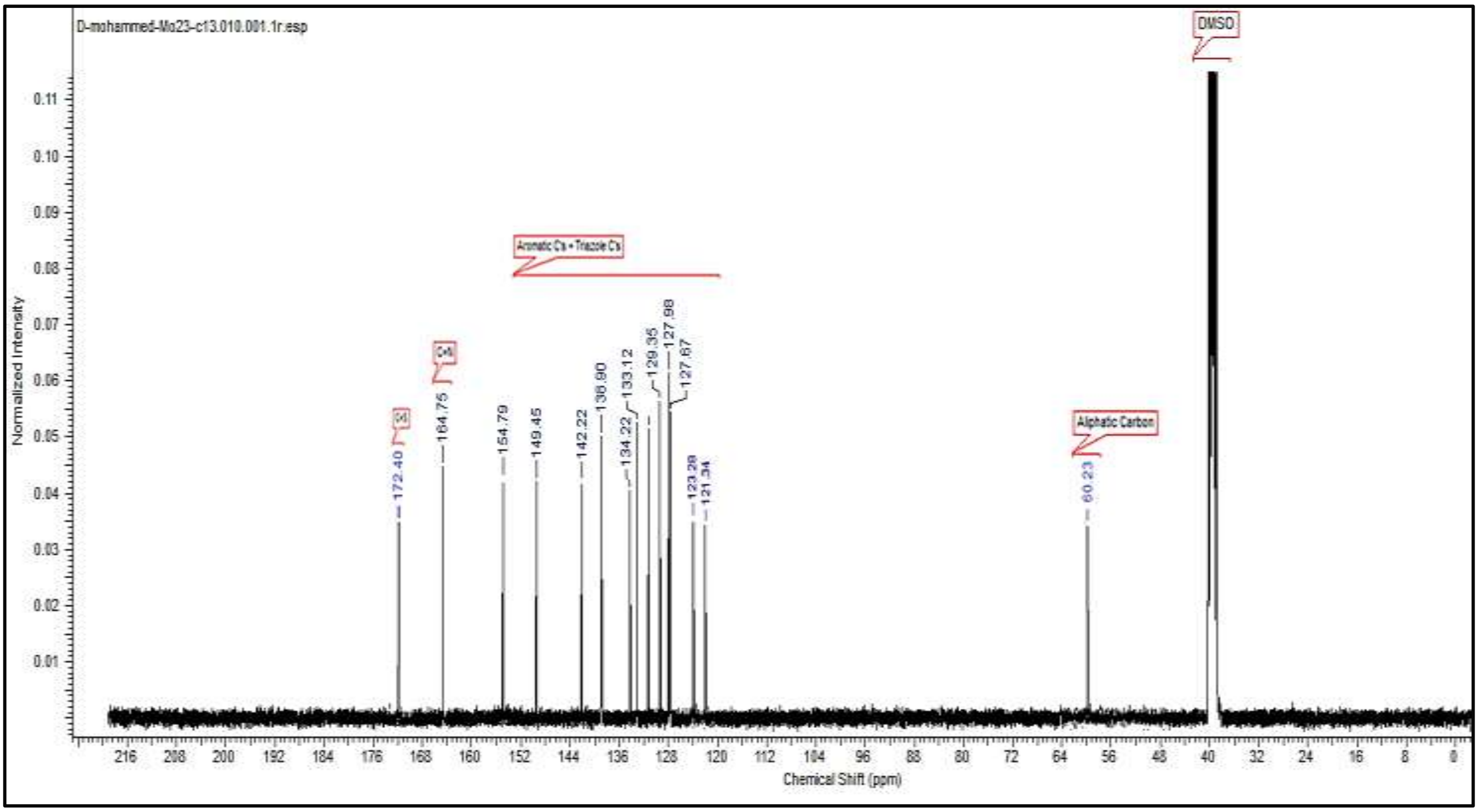

Figure S17: ^{13}C NMR spectrum (100 MHz) of compound **6d** in DMSO-d_6

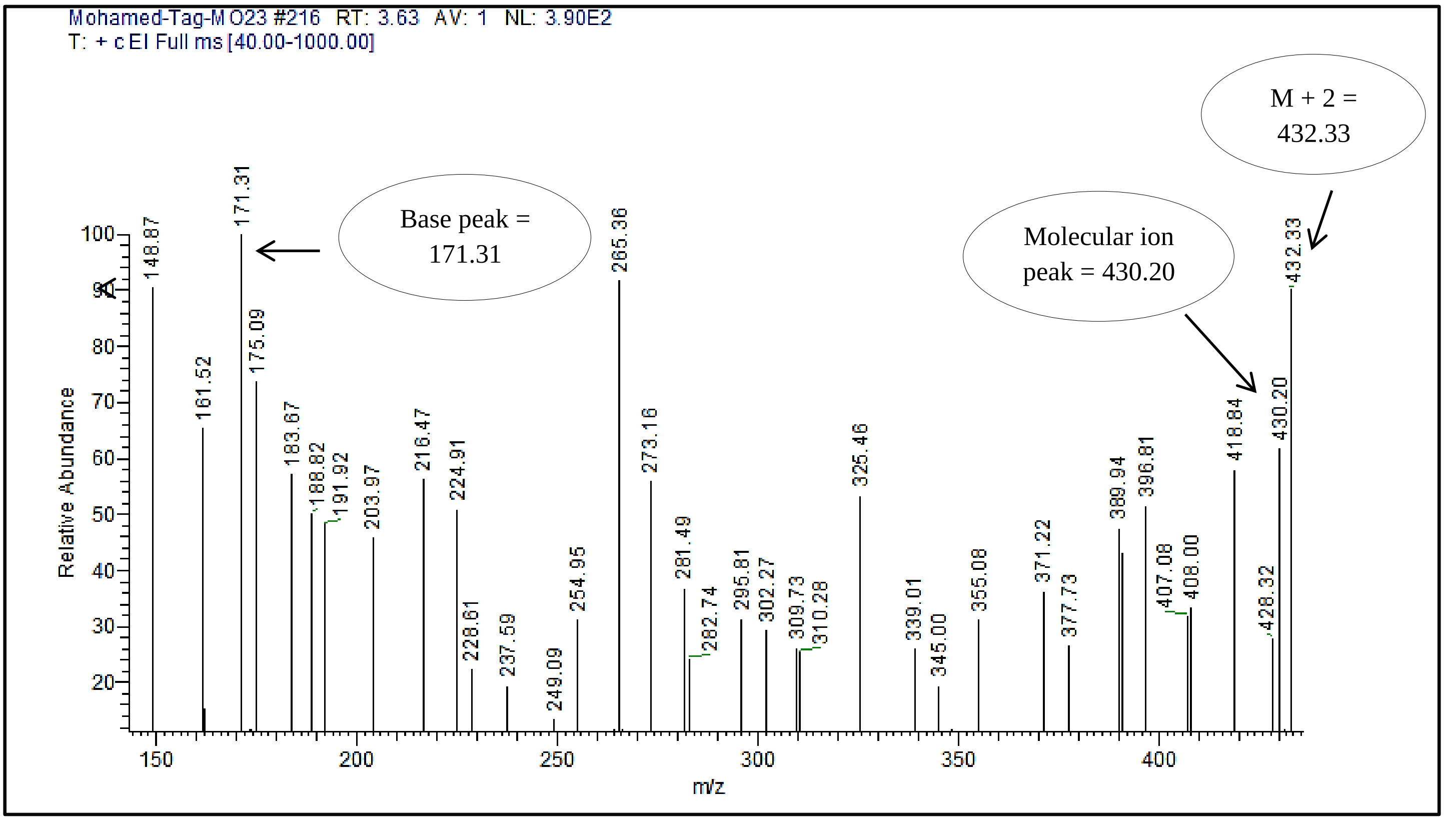

Figure S18: EI-Mass spectrum of compound **6d.**

G. ^{1}H NMR, ^{13}C NMR and EI-mass of compound 6e:

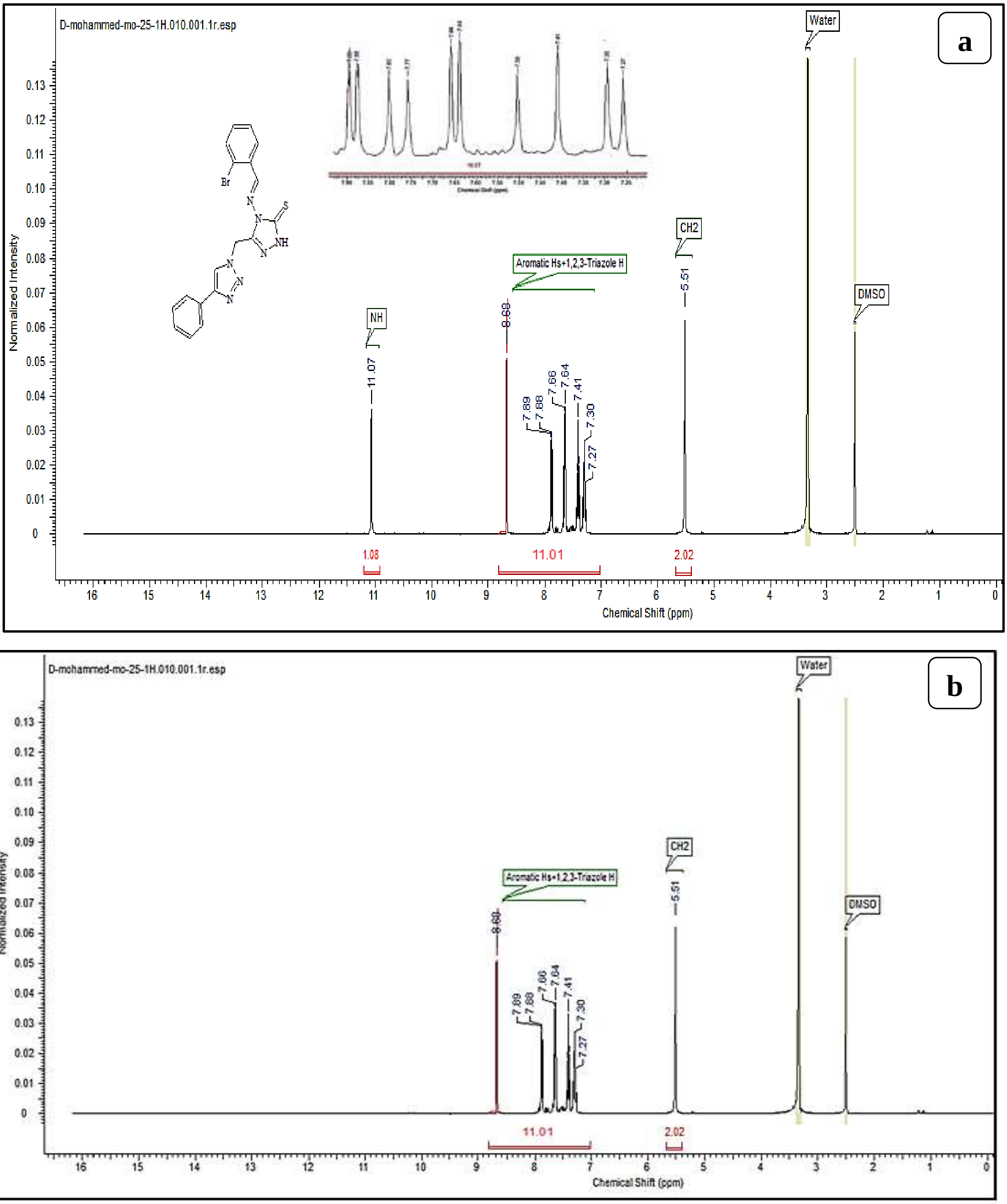

Figure S19: ^{1}H NMR spectrum (400 MHz) of compound **6e** in DMSO-d_6:

(a) before D_2O, (b) after D_2O.

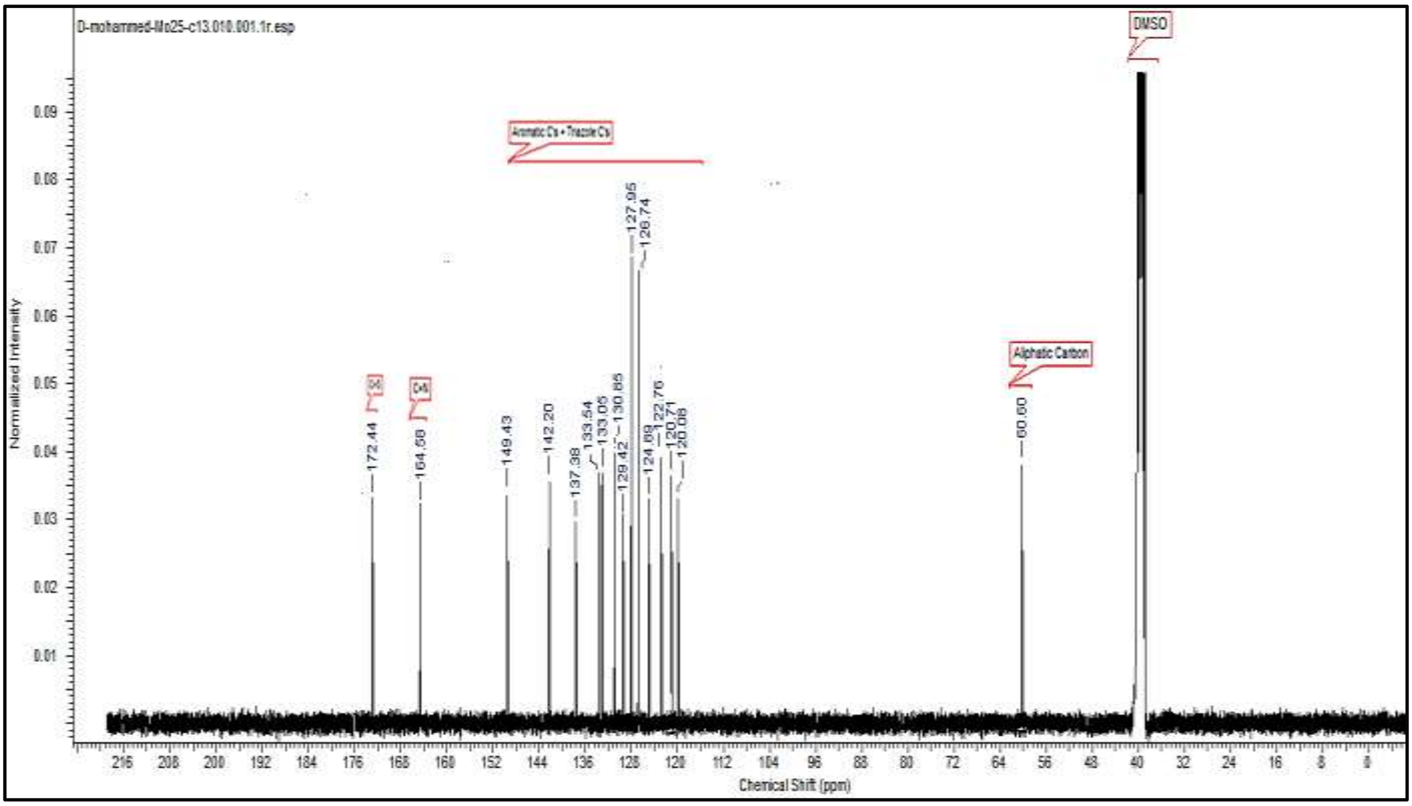

Figure S20: ^{13}C NMR spectrum (100 MHz) of compound **6e** in DMSO-d_6

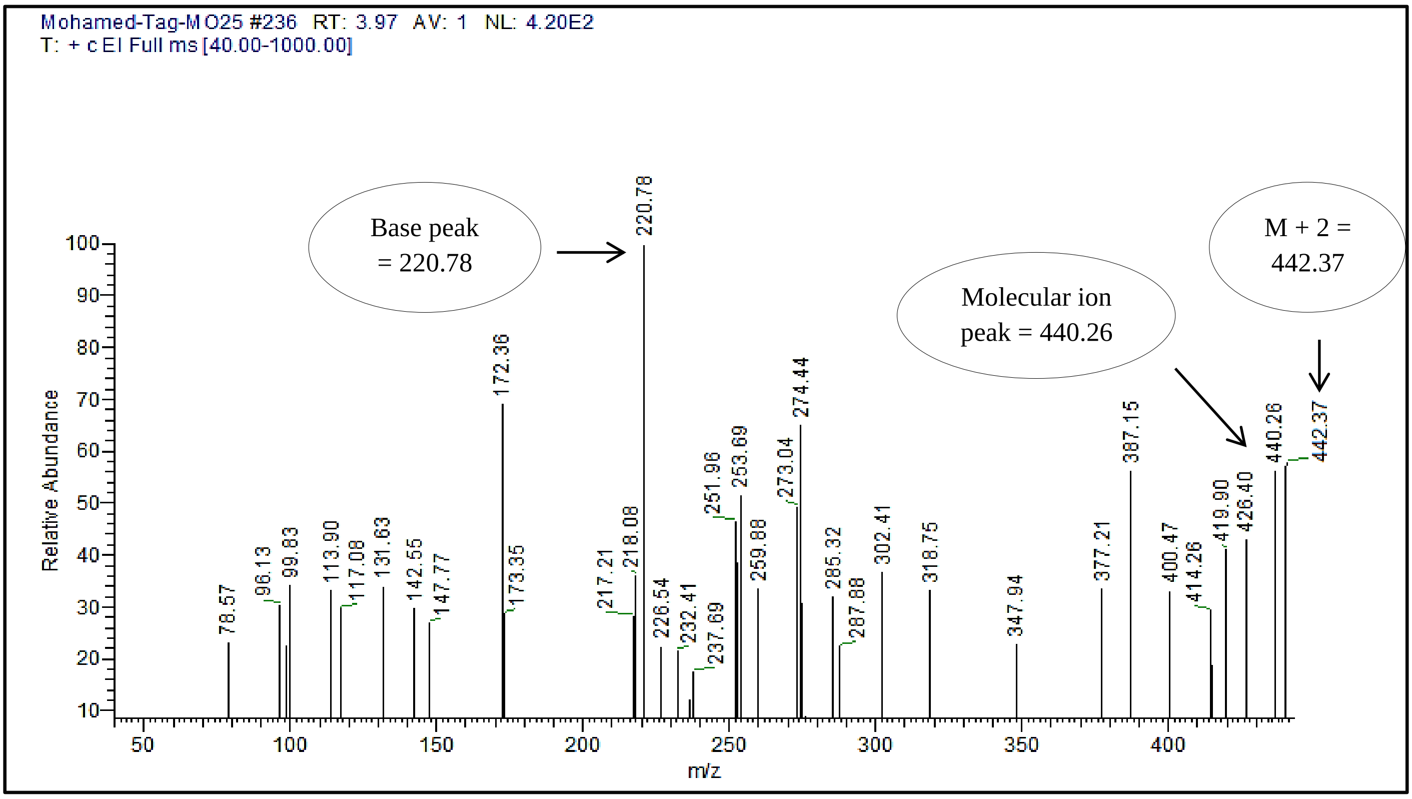

Figure S21: EI-Mass spectrum of compound **6e.**

H. ^{1}H NMR, ^{13}C NMR and EI-mass of compound 6f:

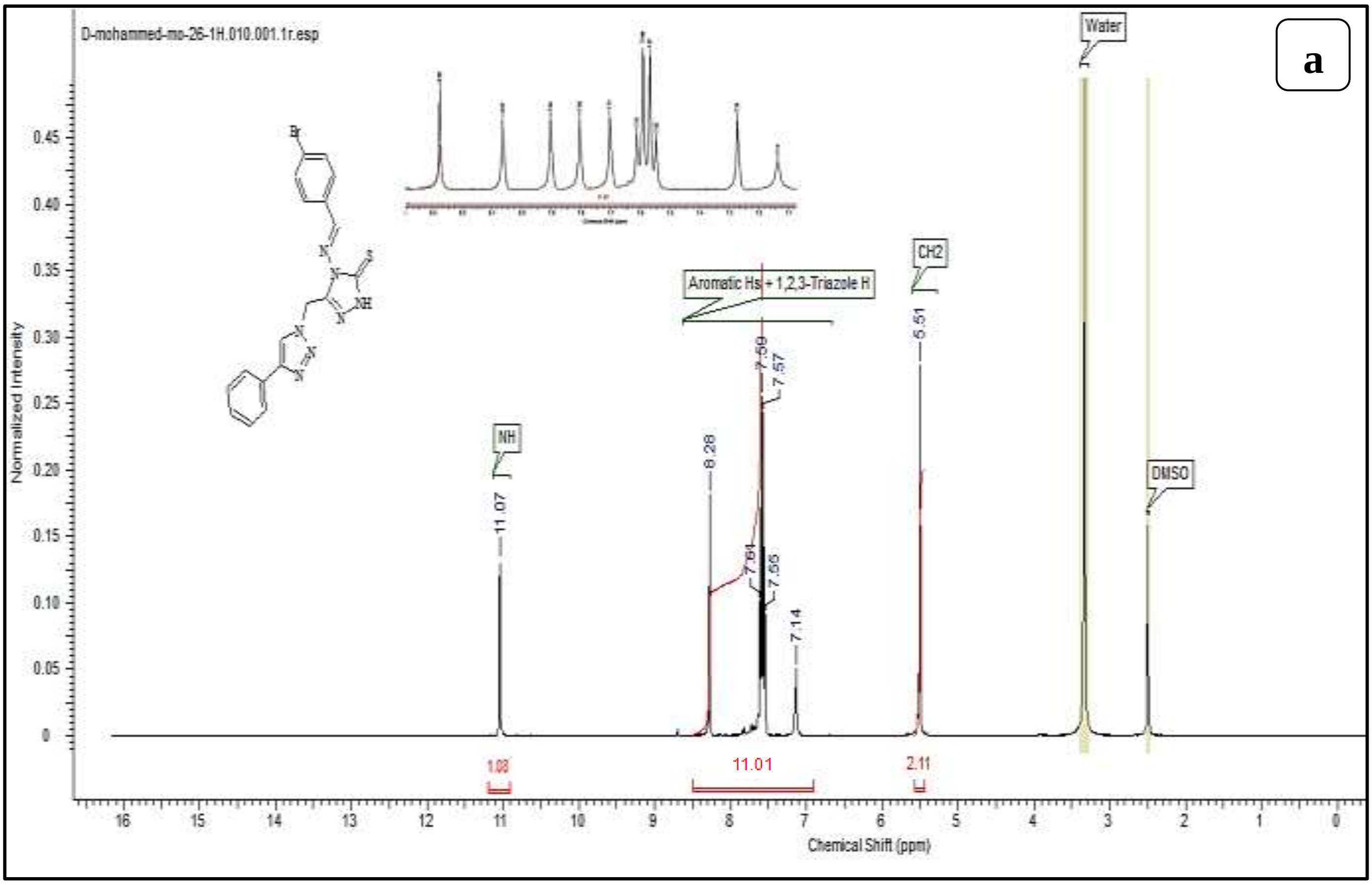

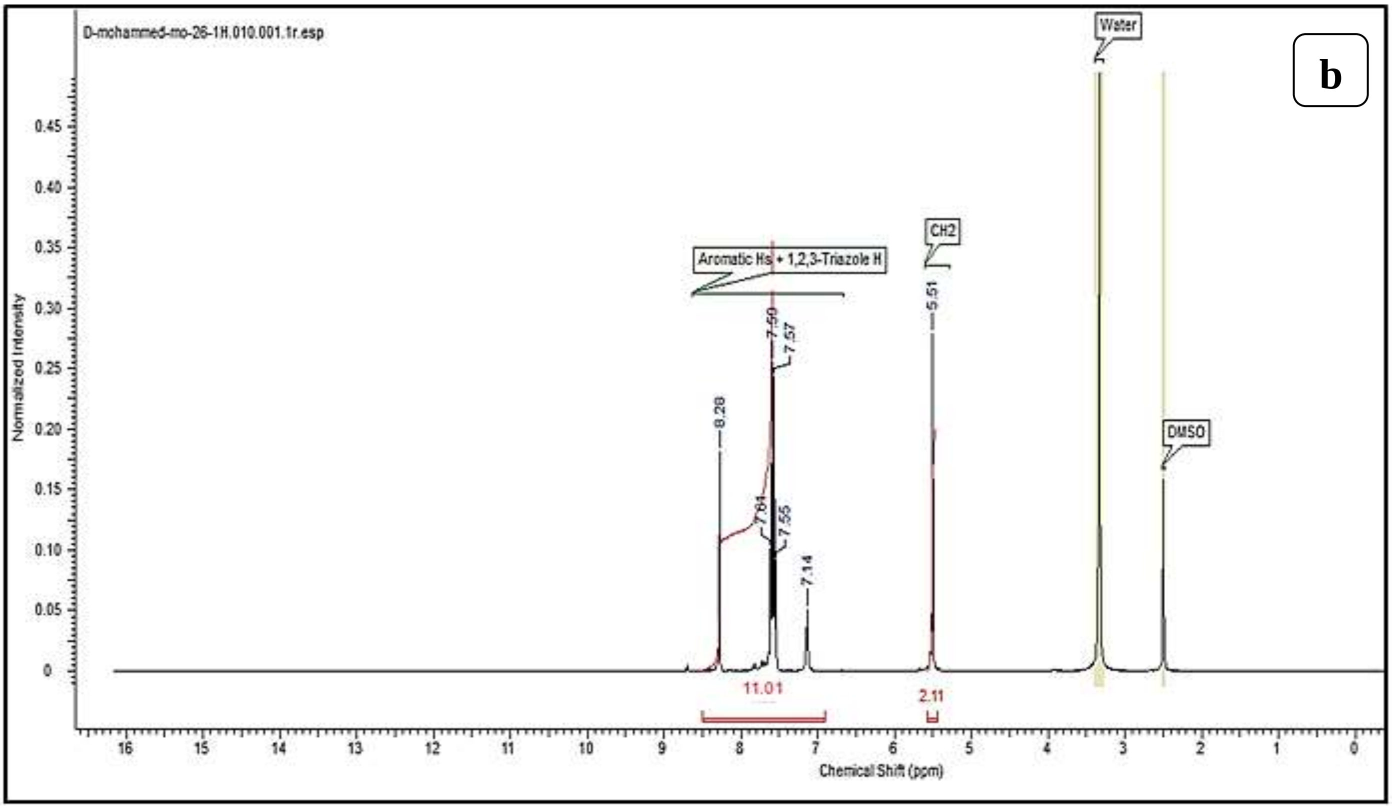

Figure S22: ^{1}H NMR spectrum (400 MHz) of compound **6f** in DMSO-d_6:

(a) before D_2O, (b) after D_2O.

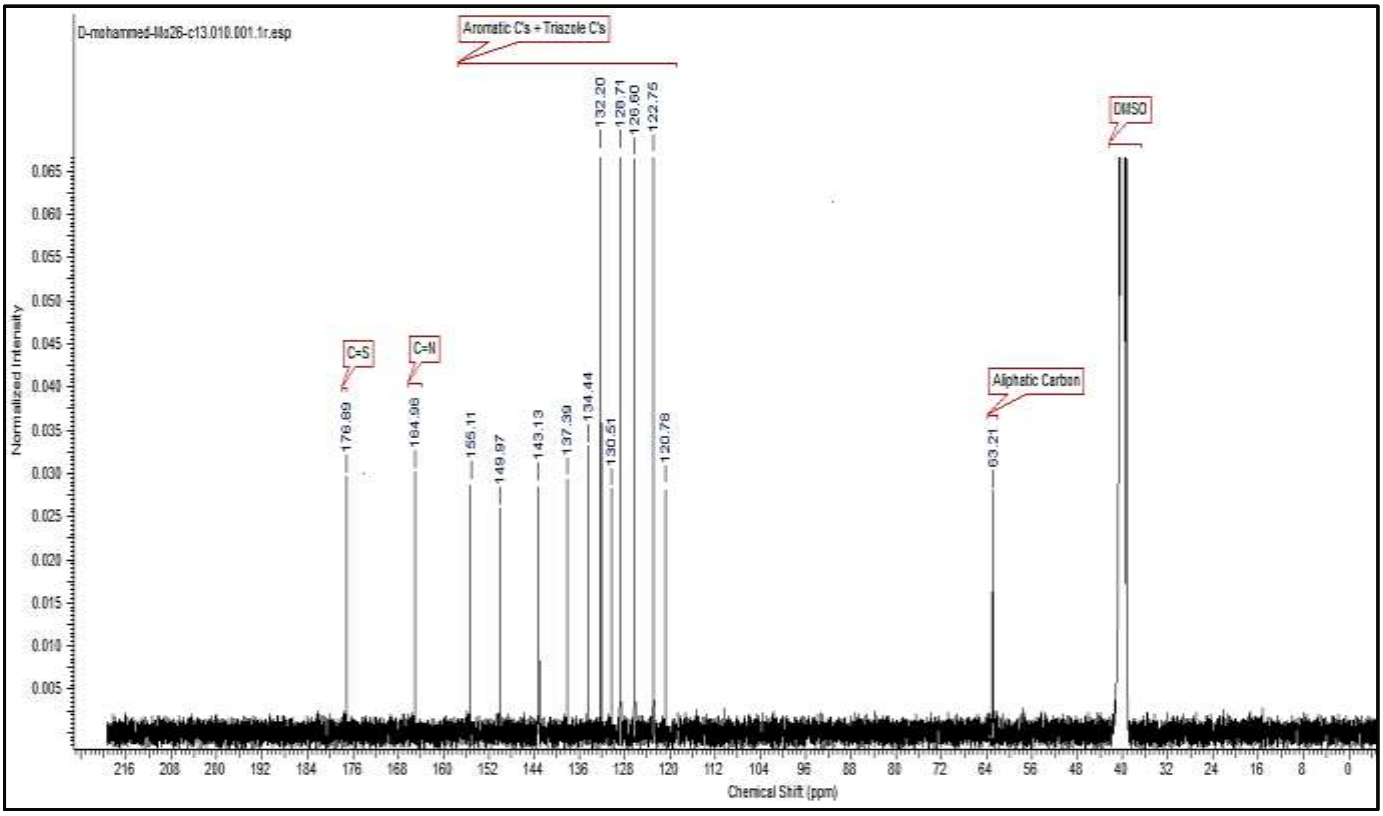

Figure S23: ^{13}C NMR spectrum (100 MHz) of compound **6f** in DMSO-d_6

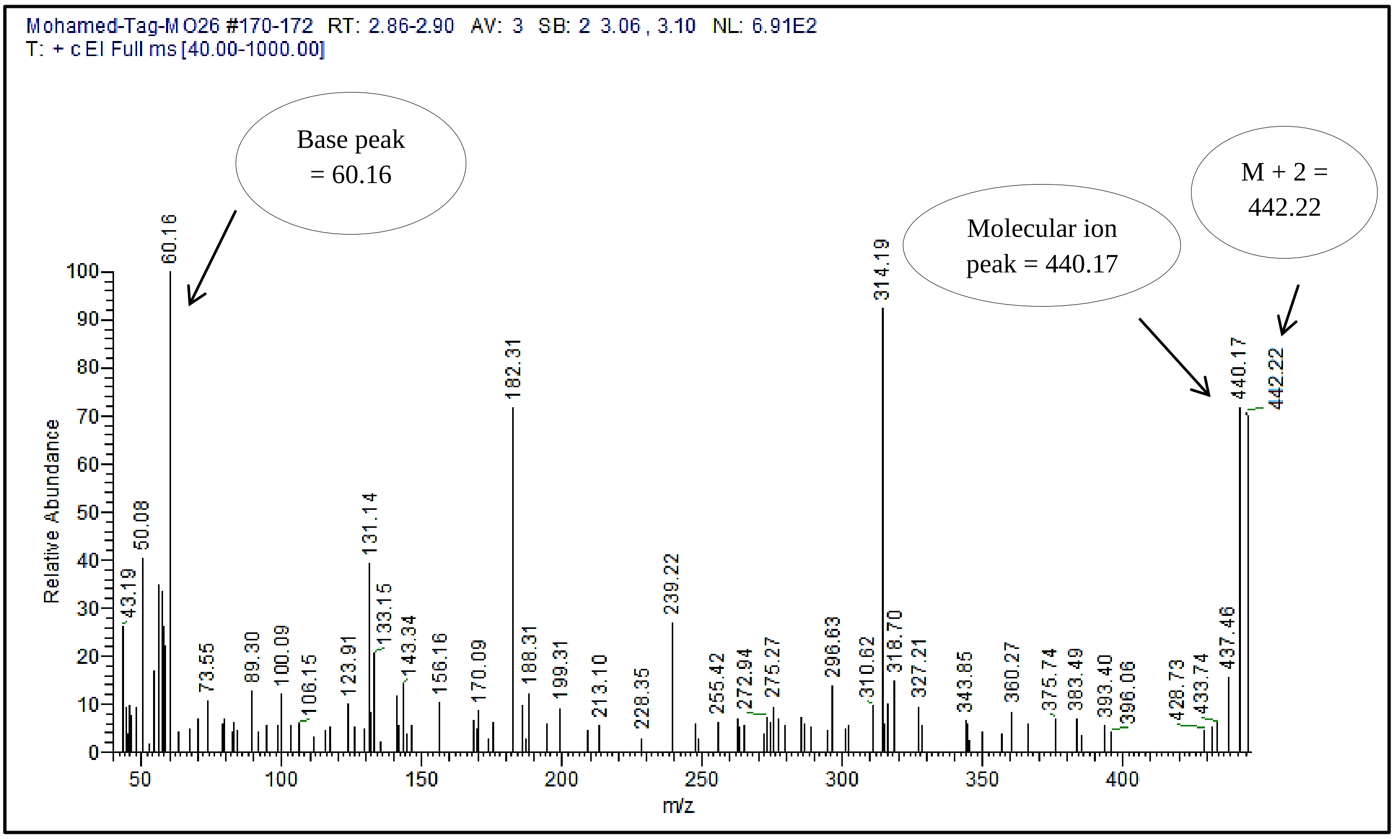

Figure S24: EI-Mass spectrum of compound **6f.**

I. ^{1}H NMR, ^{13}C NMR and EI-mass of compound 6g:

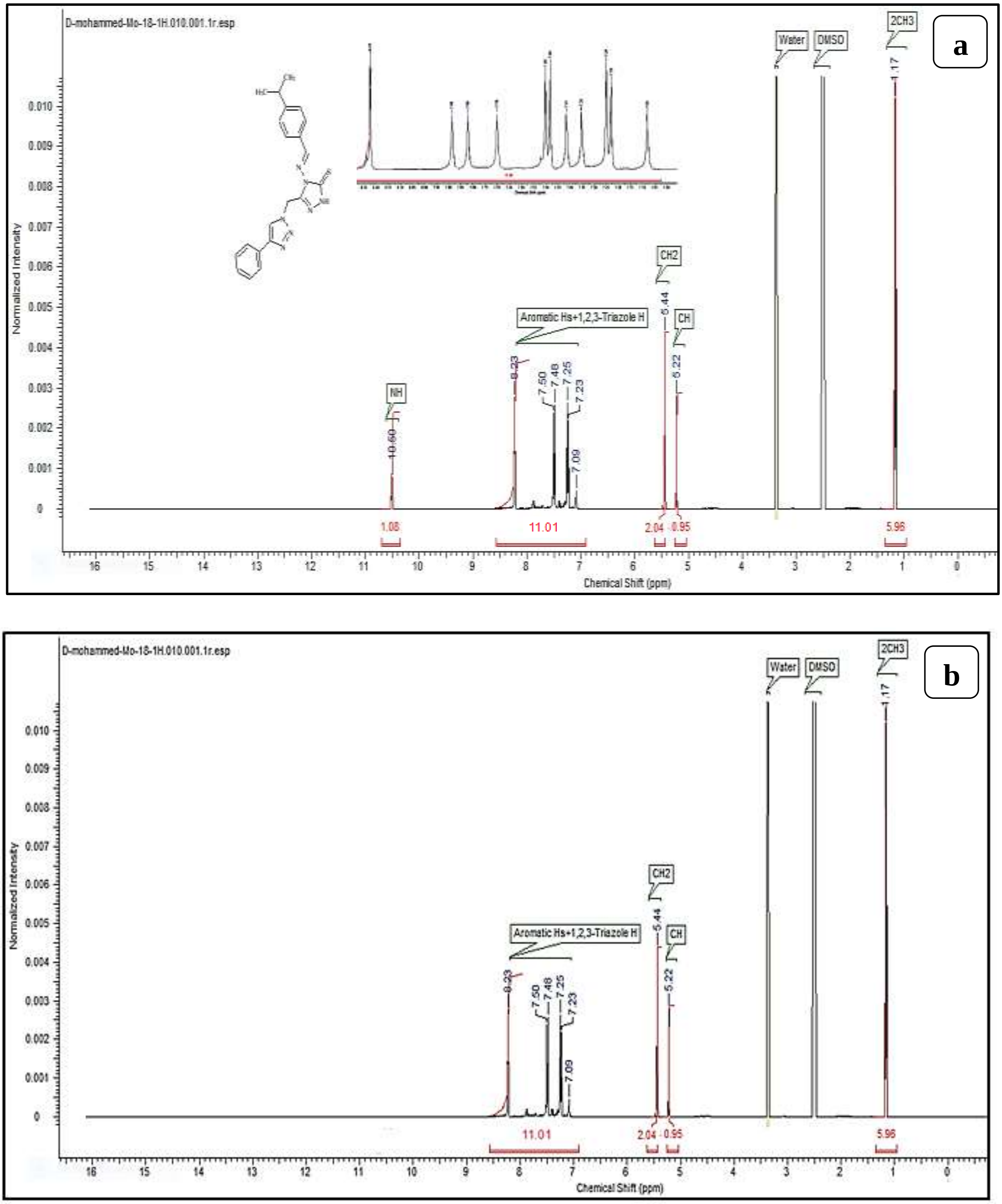

Figure S25: ^{1}H NMR spectrum (400 MHz) of compound **6g** in DMSO-d_6:

(a) before D_2O, (b) after D_2O.

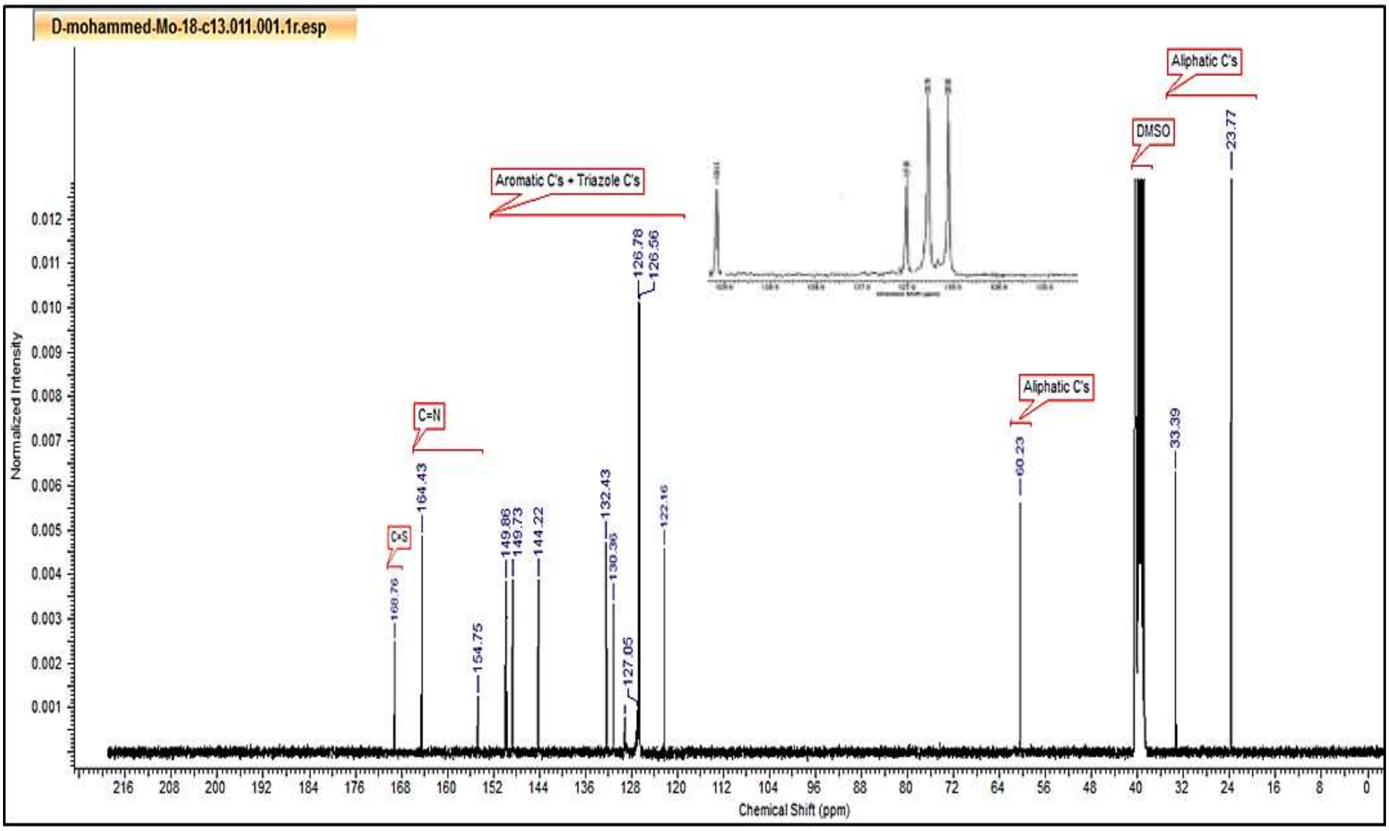

Figure S26: ^{13}C NMR spectrum (100 MHz) of compound **6g** in DMSO-d_6

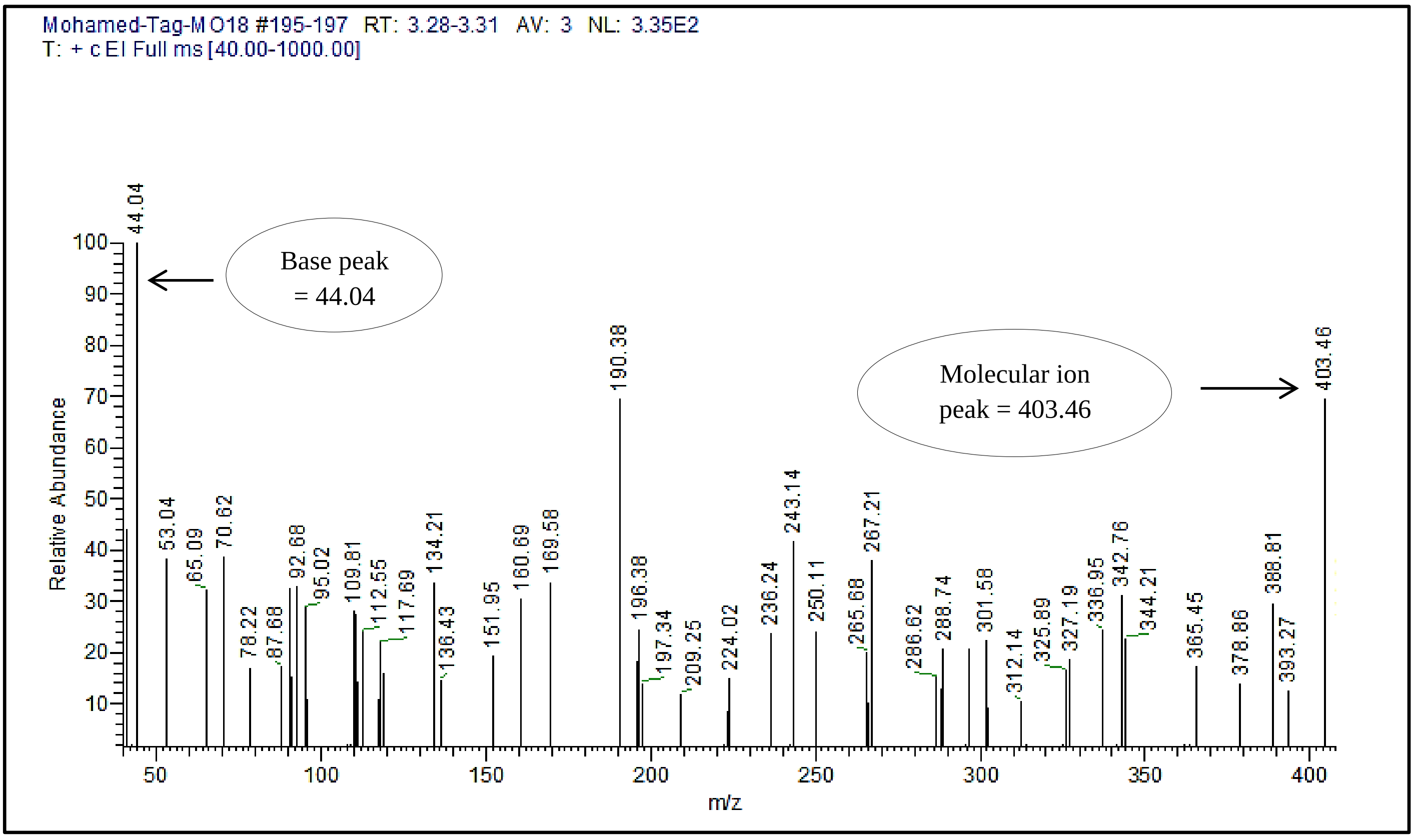

Figure S27: EI-Mass spectrum of compound **6g.**

J. ^{1}H NMR, ^{13}C NMR and EI-mass of compound 6h:

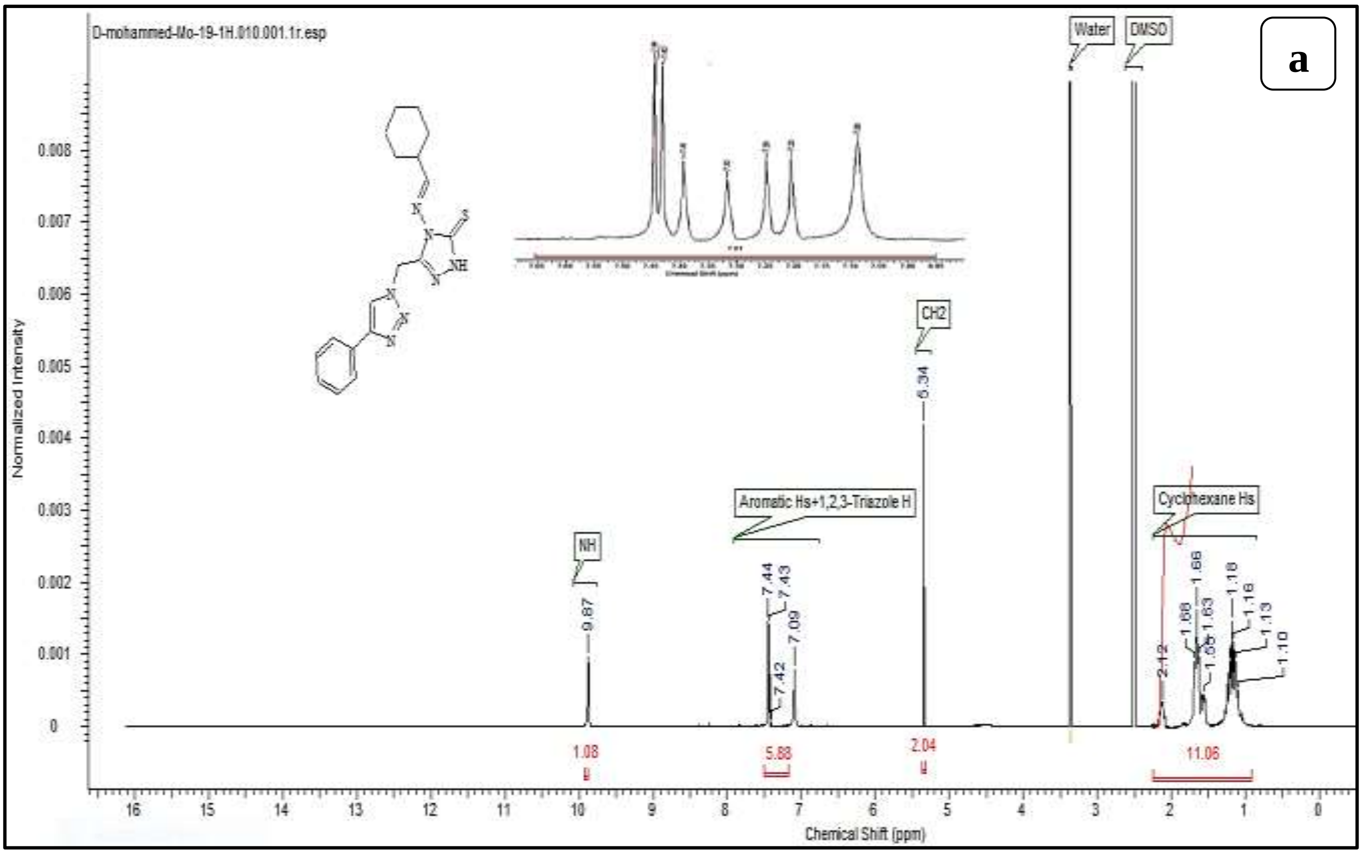

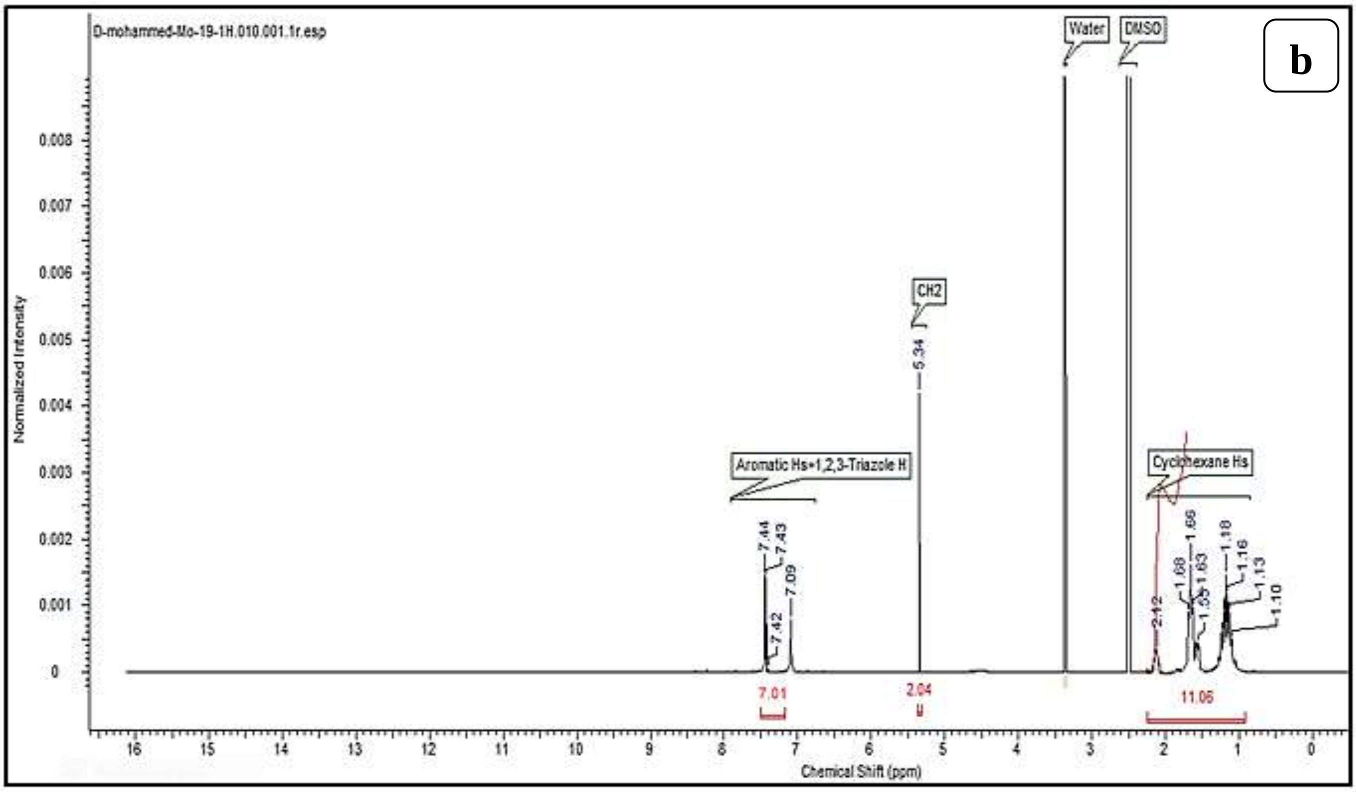

Figure S28: ^{1}H NMR spectrum (400 MHz) of compound **6h** in DMSO-d_6:

(a) before D_2O, (b) after D_2O.

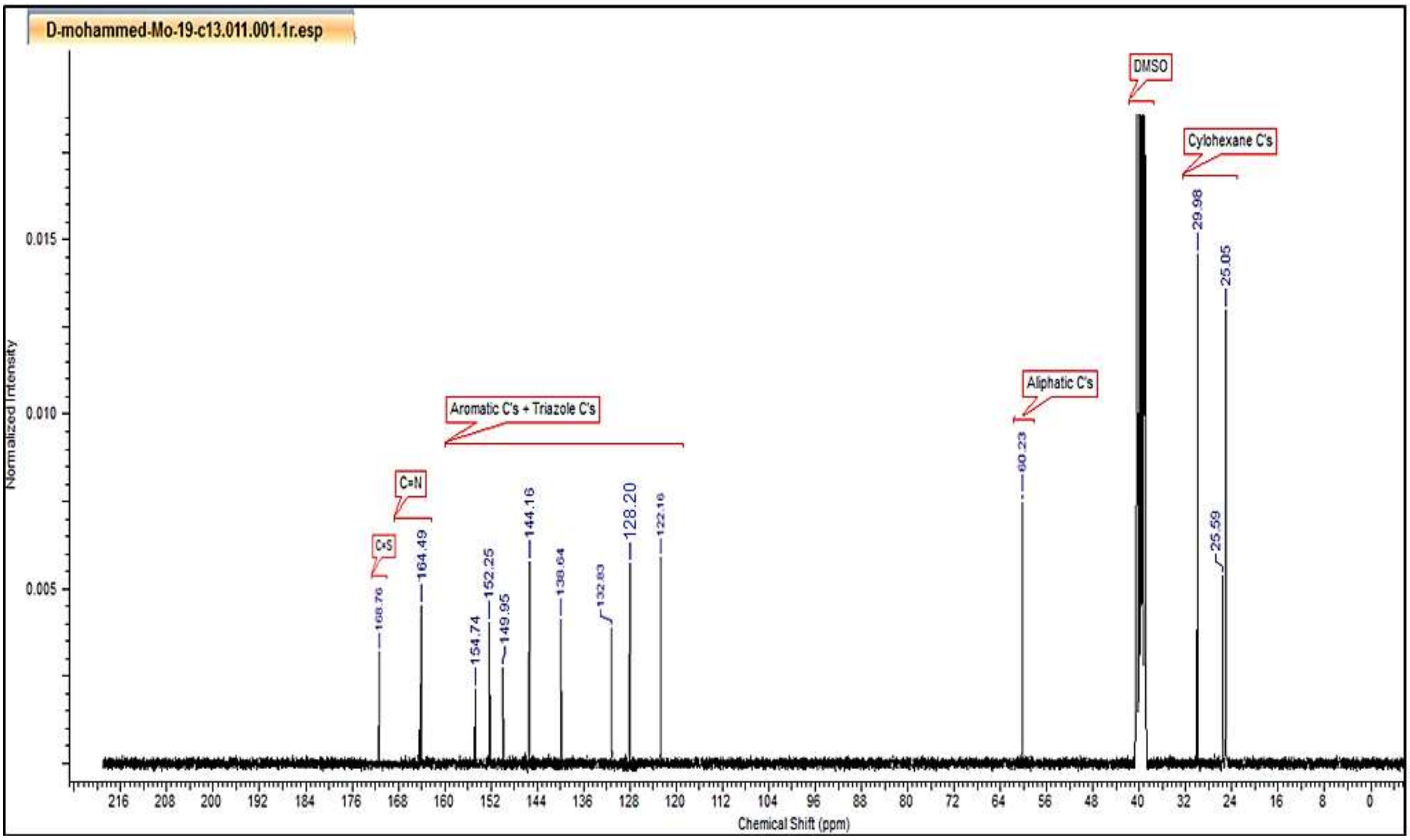

Figure S29: ^{13}C NMR spectrum (100 MHz) of compound **6h** in DMSO-d_6

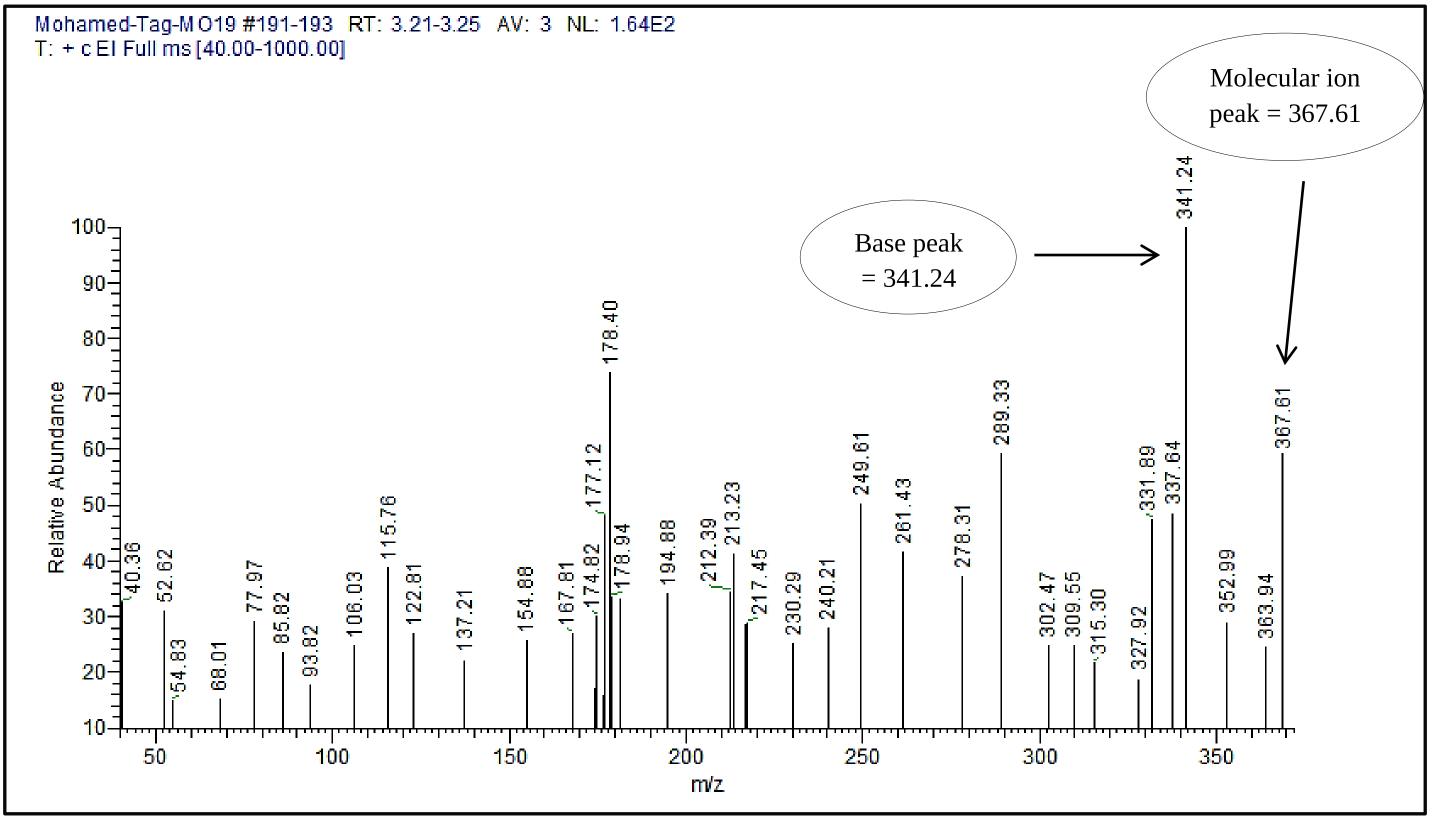

Figure S30: EI-Mass spectrum of compound **6h.**

K. ^{1}H NMR, ^{13}C NMR and EI-mass of compound 6i:

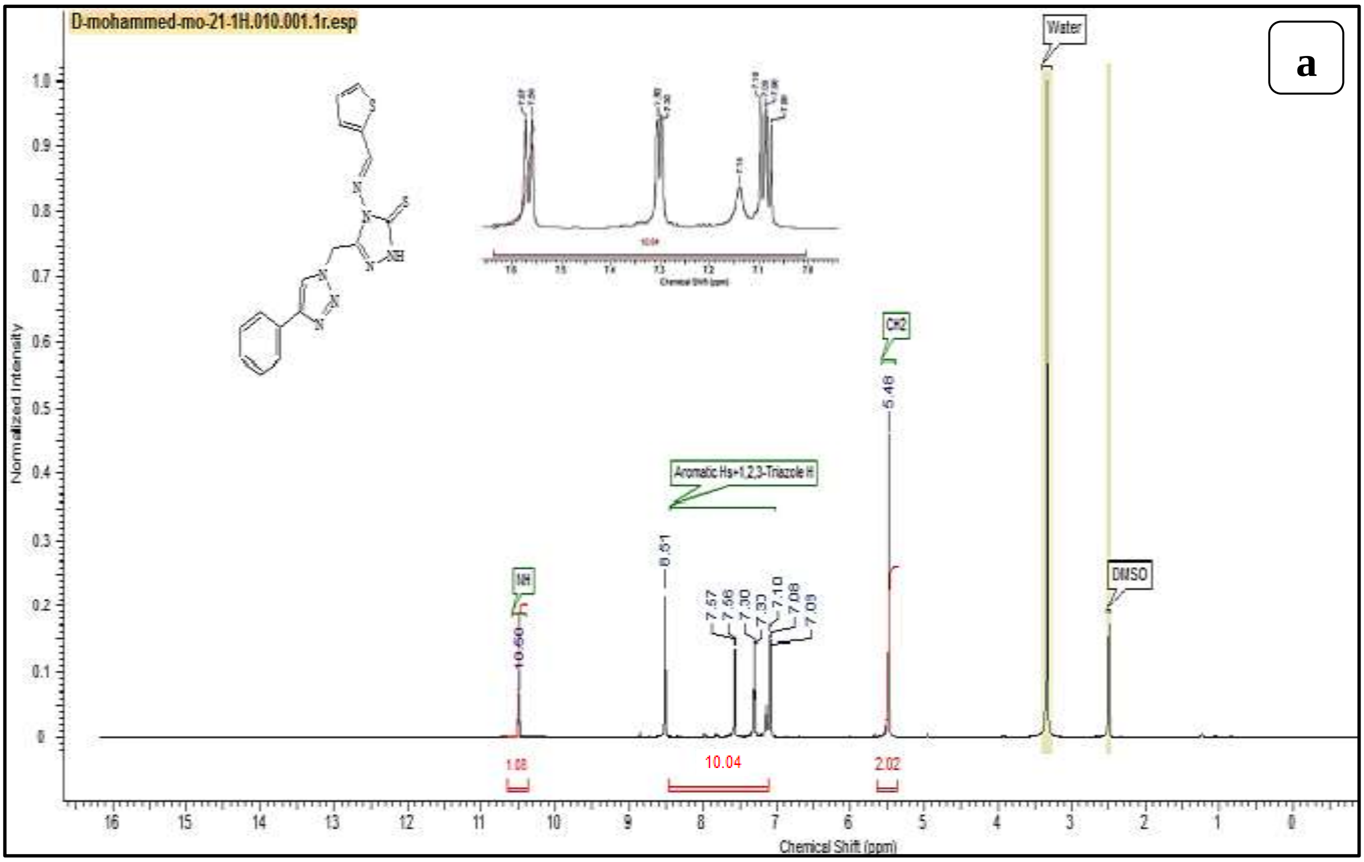

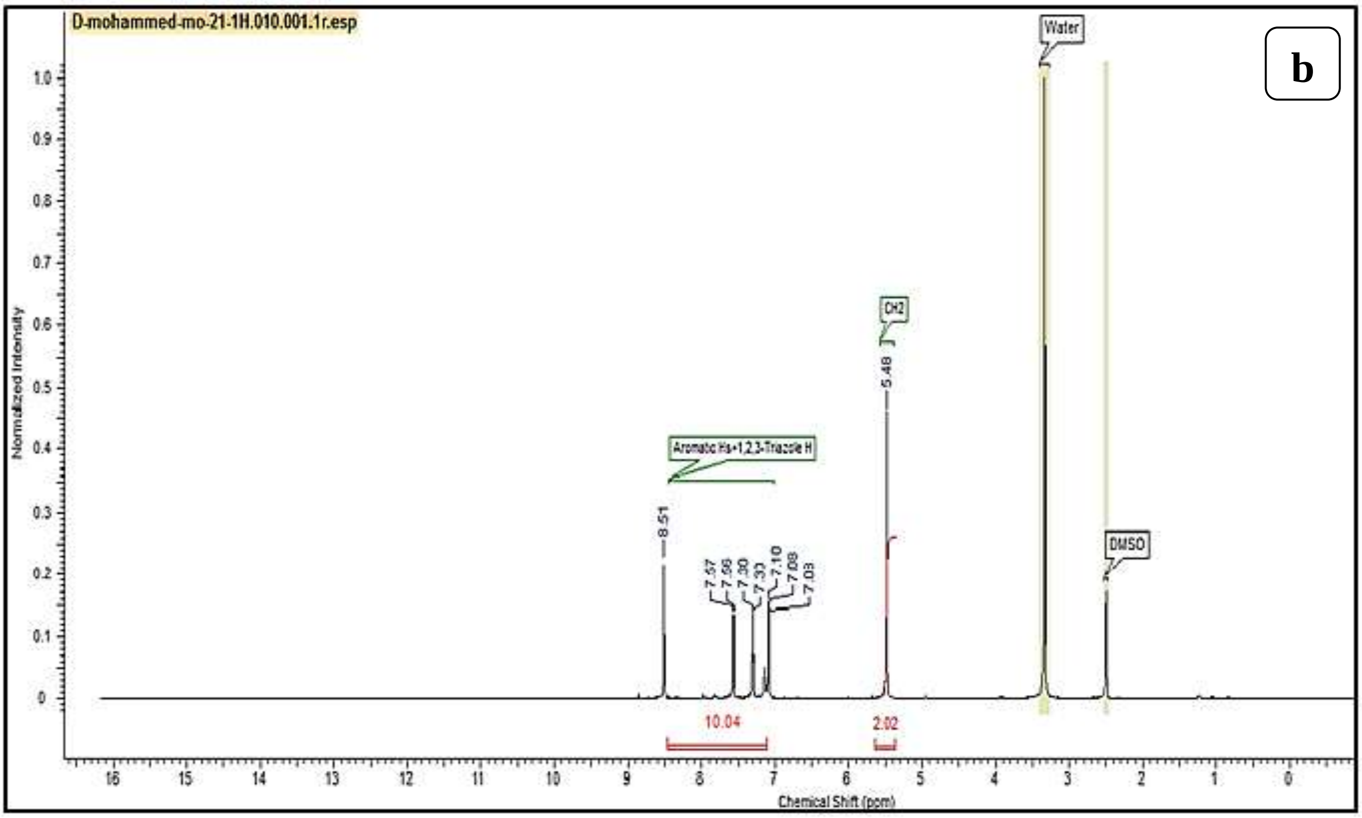

Figure S31: ^{1}H NMR spectrum (400 MHz) of compound **6i** in DMSO-d_6:

(a) before D_2O, (b) after D_2O.

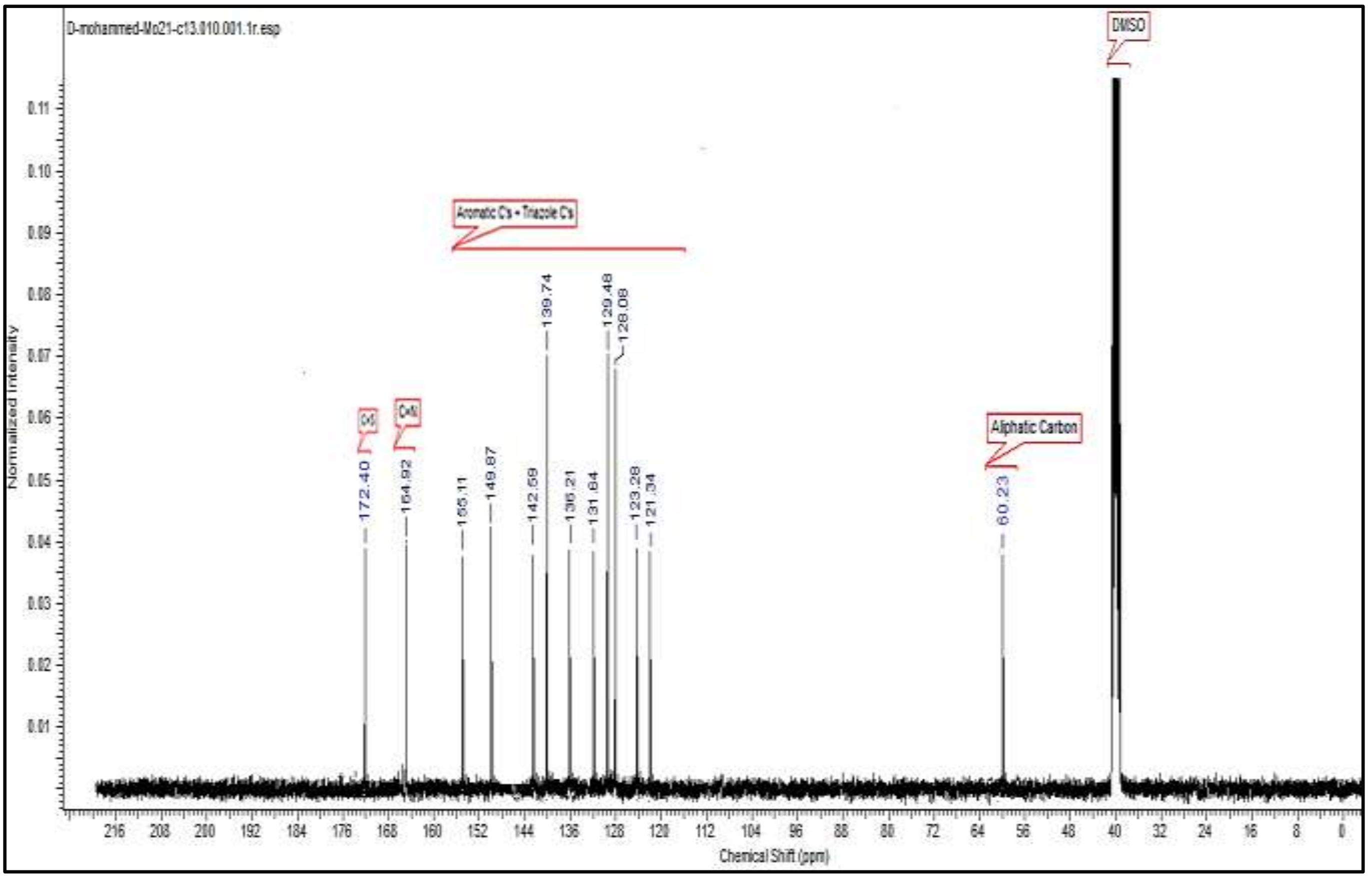

Figure S32: ^{13}C NMR spectrum (100 MHz) of compound **6i** in DMSO-d_6

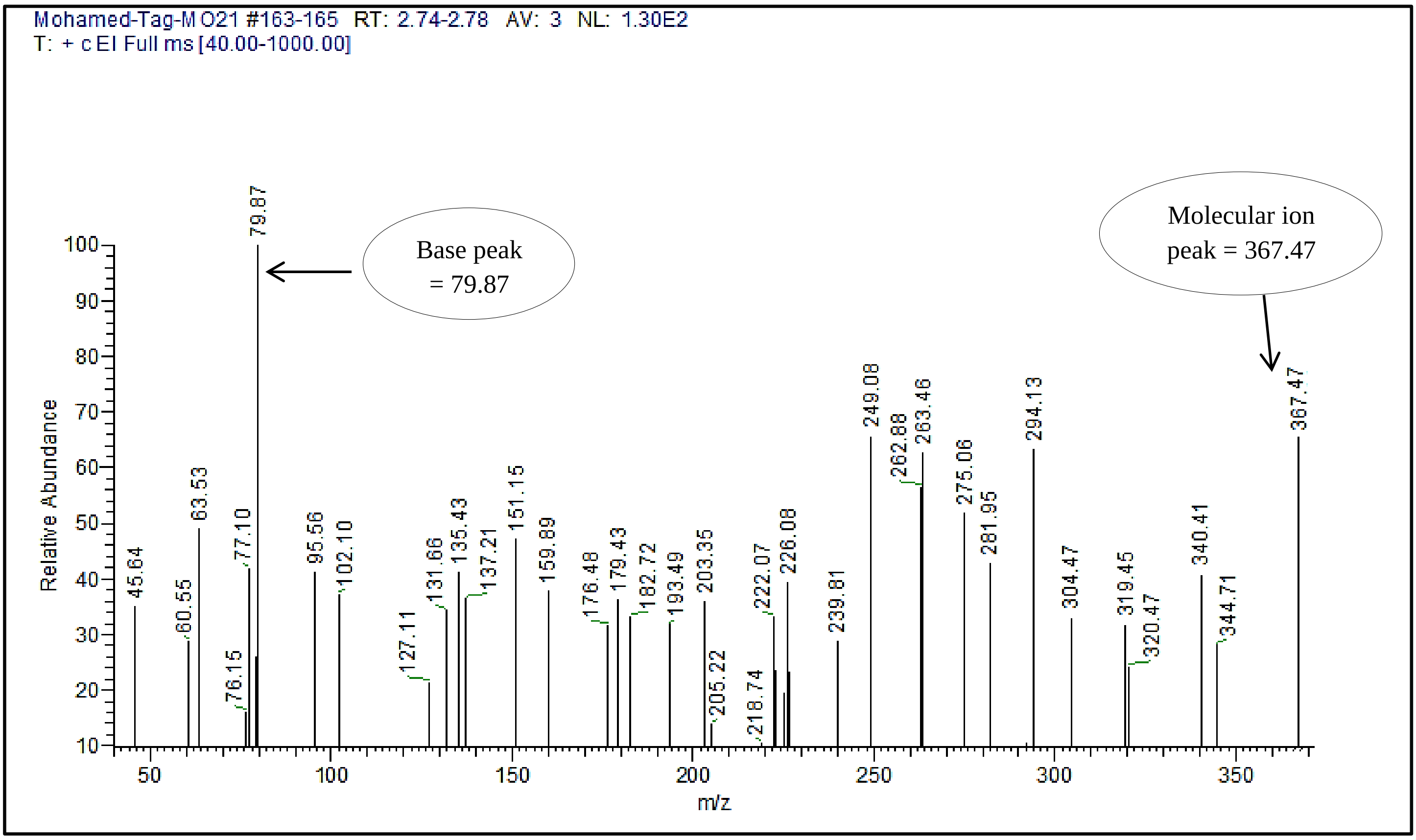

Figure S33: EI-Mass spectrum of compound **6i.**

Appendix SA

3. EXPERIMENTAL

3.1. Chemistry

General Details

Pre-coated silica gel plates (kieselgel 0.25 mm, 60G F_{254}, Merck, Germany) were used for TLC monitoring of the chemical reactions. Hexane: Ethyl acetate (2:1 *v/v*) was used as a developing solvent system, unless otherwise specified and spots were detected by using ultraviolet lamp at 254 nm wavelength (Spectroline, model CM-10, USA). Melting points (M.P.) were determined on an electrothermal melting point apparatus [Stuart Scientific, model SMP3, UK], and were uncorrected. Infra-red (IR) spectra (KBr discs) for compounds **(5a-h)** were recorded on thermo scientific nicolet IS10 FT IR spectrometer (thermo Fischer scientific, USA) at Faculty of science, Assiut University, Assiut, Egypt. Data acquisition was performed on Omnic software. ^{1}H-NMR and ^{13}C-NMR spectra were measured on (Avance -III, 400 MHz, High performance FT-NMR spectrum, Bruker biospin international AG-Switzeraland) at Faculty of Pharmacy, Ain-Shams University, Cairo, Egypt. Chemical shifts are expressed in δ-values (*ppm*) relative to TMS as an internal standard, using the appropriate solvent as specified. Mass spectra for all new compounds were carried out on Direct Inlet part to mass analyzer in Thermo Scientific GCMS model ISQ at the Regional Center for Mycology and Biotechnology (RCMB), Al-Azhar University, Nasr City, Cairo, Egypt. Elemental microanalysis was performed on elemental analyzer model flash 2000 thermo fisher at the regional center for mycology and biotechnology (RCMB), faculty of science, Al-Azhar university, Nasr city, Cairo, Egypt for all new compounds**.**

3.2. Biological evaluation

3.2.1 Cell Viability assay (MTT assay)

MTT assay was performed to investigate the effect of the synthesized compounds on mammary epithelial cells (MCF-10A). The cells were propagated in medium consisting of Ham's F-12 medium/ Dulbecco's modified Eagle's medium (DMEM) (1:1) supplemented with 10% foetal calf serum, 2 mM glutamine, insulin (10 μg/mL), hydrocortisone (500 ng/mL) and epidermal growth factor (20 ng/mL). Trypsin ethylenediamine tetra acetic acid (EDTA) was used to passage the cells after every 2-3 days. 96-well flat-bottomed cell culture plates were used to seed the cells at a density of 104 cells mL^{-1}. The medium was aspirated from all the wells of culture plates after 24 h followed by the addition of synthesized compounds (in 200 μL medium to yield a final concentration of 0.1% (v/v) dimethyl sulfoxide) into individual wells of the plates. Four wells were designated to a single compound. The plates were allowed to incubate at 37°C for 96 h. Afterwards, the medium was aspirated and 3-[4,5-dimethylthiazol-2-yl]-2,5-diphenyltetrazolium bromide (MTT) (0.4 mg/mL) in medium was added to each well and subsequently incubated for 3 h. The medium was aspirated and 150 μL dimethyl sulfoxide (DMSO) was added to each well. The plates were vortexed followed by the measurement of absorbance at 540 nm on a microplate reader. The results were presented as inhibition (%) of proliferation in contrast to controls comprising 0.1% DMSO.

3.2.2. Assay for antiproliferative effect

To explore the antiproliferative potential of compounds propidium iodide fluorescence assay was performed using different cell lines such as Panc-1 (pancreas cancer cell line), MCF-7 (breast cancer cell line), HT-29 (colon cancer cell line) and A-549 (epithelial cancer cell line), respectively. To calculate the total nuclear DNA, a fluorescent dye (propidium iodide, PI) is used

which can attach to the DNA, thus offering a quick and precise technique. PI cannot pass through the cell membrane and its signal intensity can be considered as directly proportional to quantity of cellular DNA. Cells whose cell membranes are damaged or have changed permeability are counted as dead ones. The assay was performed by seeding the cells of different cell lines at a density of 3000-7500 cells/well (in200µl medium) in culture plates followed by incubation for 24h at 37 °C in humidified 5%CO2/95% air atmospheric conditions. The medium was removed; the compounds were added to the plates at 10 µM concentrations (in 0.1% DMSO) in triplicates, followed by incubation for 48h. DMSO (0.1%) was used as control. After incubation, medium was removed followed by the addition of PI (25 µl, 50µg/mL in water/medium) to each well of the plates. At -80 °C, the plates were allowed to freeze for 24 h, followed by thawing at 25 °C. A fluorometer (Polar-Star BMG Tech) was used to record the readings at excitation and emission wavelengths of 530 and 620 nm for each well. The percentage cytotoxicity of compounds was calculated using the following formula:

$$\% \text{ Cytotoxicity} = \frac{A_C - A_{TC}}{A_C} \times 100$$

Where A*TC*= Absorbance of treated cells and AC= Absorbance of control. Erlotinib was used as positive control in the assay.

3.2.2. Aromatase inhibitory assay

The aromatase inhibitory effect was performed using the method reported by Stressor et al.[35]. This method was carried out according to the Gentest kit using CYP19 enzyme and DBF as a fluorometric substrate. DBF was dealkylated by aromatase and then hydrolyzed to give the fluorescein product. Briefly, 100 µL of cofactor, containing 78.4 µL of 50 mM phosphate buffer (pH 7.4); 20 µL of 20x NADPH-generating system (26 mM NADP+, 66 mM glucose-6-phosphate,

and 66 mM $MgCl_2$); and 1.6 μL of 100 U/mL glucose-6-phosphate dehydrogenase, was pipetted into a 96-well plate and preincubated in 37 $_{o}$C (water bath) for 10 min. The reaction was initiated by addition of 100 μL of enzyme/substrate mixture containing 77.3 μL of 50 mM phosphate buffer (pH 7.4); 12.5 μL of 16 pmol/mL CYP19; 0.2 μL of 0.2 mM DBF, and 10 μL of 0.25 mM diluted tested sample or 10% DMSO as a negative control or letrozole as a positive control. Fluorescence signal was recorded using an excitation wavelength of 490 nm and emission wavelength of 530 nm with cutoff 515 nm. Percentage of inhibition (%inhibition) was calculated as shown in Equation (1). Samples with % inhibition greater than 50 were further diluted and assayed in triplicate. Finally, IC_{50} values were determined by plot of concentrations versus % inhibition.

$$\%\ inhibition = 100 - [(sample - blank)/(DMSO - blank) \times 100]$$

The proposed Mechanism for preparation of compound **4**

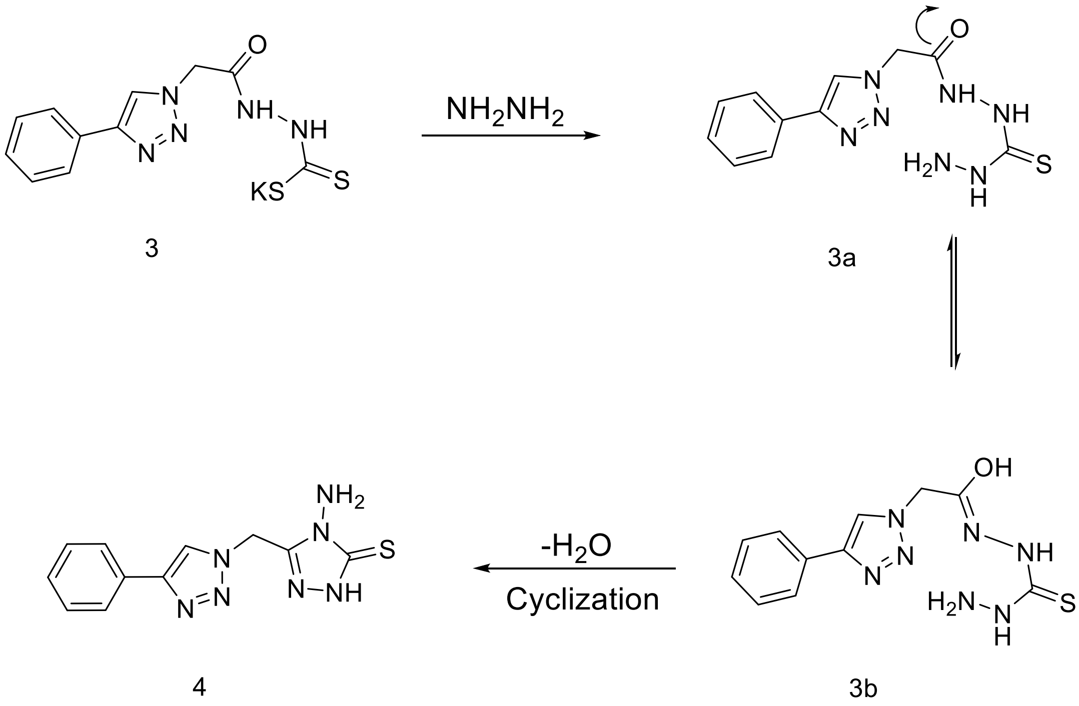

Supplement: Supplementary file 1 [file molecules-28-07092-s001.zip › molecules-2634786-supplementary.pdf]
